# Oxygen-Dependent Interactions between the Ruthenium Cage and the Photoreleased Inhibitor in NAMPT-Targeted Photoactivated Chemotherapy

**DOI:** 10.1021/acs.jmedchem.4c00589

**Published:** 2024-06-26

**Authors:** Selda Abyar, Luojiao Huang, Yurii Husiev, Ludovic Bretin, Bobby Chau, Vadde Ramu, Jacob Hendricus Wildeman, Kimberley Belfor, Lukas S. Wijaya, Vera E. van der Noord, Amy C. Harms, Maxime A. Siegler, Sylvia E. Le Dévédec, Sylvestre Bonnet

**Affiliations:** † Leiden Institute of Chemistry, Leiden University, Gorlaeus Laboratories, PO Box 9502, Leiden 2300 RA, The Netherlands; ‡ Leiden Academic Centre for Drug Research, Leiden University, Gorlaeus Laboratories, PO Box 9502, Leiden 2300 RA, The Netherlands; § Department of Chemistry, Johns Hopkins University, 3400 N Charles St, Baltimore, Maryland 21218, United States

## Abstract

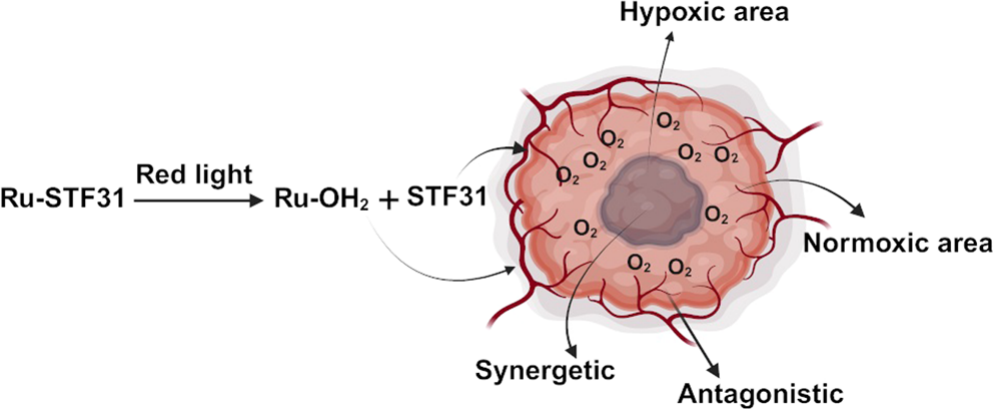

Photoactivated chemotherapy agents form a new branch of physically targeted anticancer agents with potentially lower systemic side effects for patients. On the other hand, limited information exists on the intracellular interactions between the photoreleased metal cage and the photoreleased anticancer inhibitor. In this work, we report a new biological study of the known photoactivated compound **Ru-STF31** in the glioblastoma cancer cell line, U87MG. **Ru-STF31** targets nicotinamide phosphoribosyltransferase (NAMPT), an enzyme overexpressed in U87MG. **Ru-STF31** is activated by red light irradiation and releases two photoproducts: the ruthenium cage and the cytotoxic inhibitor **STF31**. This study shows that **Ru-STF31** can significantly decrease intracellular NAD^+^ levels in both normoxic (21% O_2_) and hypoxic (1% O_2_) U87MG cells. Strikingly, NAD^+^ depletion by light activation of **Ru-STF31** in hypoxic U87MG cells could not be rescued by the addition of extracellular NAD^+^. Our data suggest an oxygen-dependent active role of the ruthenium photocage released by light activation.

## Introduction

According to Global Cancer Statistics, 3 08 102 new cases of brain and central nervous system (CNS) cancers were diagnosed in 2020 and 2 51 329 cancer-related deaths occurred in the same year.^1^ Gliomas represent 40% of all brain tumors, which makes them the most common and deadly human primary brain tumors. About half of all newly diagnosed gliomas correspond to glioblastoma (GBM), which is the most malignant type of brain cancer (grade IV) with a median overall survival of approximately 14–17 months in current clinical trials^2,3^ and around 12 months in population-based studies.^1,3,4^ Because of the heterogeneous nature of GBM, its treatment includes maximally safe surgical resection with subsequently parallel chemotherapy (Temozolomide) and fractionated radiotherapy.^5^ Consistently low tumor oxygenation (<1% O_2_), also known as “chronic hypoxia”, is the main concern for GBM patients since it promotes cancer cells spreading into healthy brain tissues to evade the adverse hypoxic microenvironment.^6^ Although resection can achieve a reduction in the primary tumor burden, the observation that more than 80% of the recurrences are situated adjacent to the resection cavity suggests some utility for therapeutic platforms targeting this region.^7^ Previously, it has been shown that due to the weakly differentiated neoplastic astrocytes that do not release factors vital for the brain-blood barrier (BBB) function, leaky interendothelial tight junctions exist in human glioma. It was also demonstrated that BBB stability in lower-grade gliomas is better than that in GBM.^8^ As the degree of BBB disruption differs with the malignancy of the tumor, treatment of low-grade brain tumors is still a challenging task, because of the presence of almost intact BBB.^9^ Recently, the United States Food and Drug Administration (FDA) approval of 5-aminolevulinic acid (5-ALA) for fluorescence-guided resection (FGR) of tumors rehabilitated interests in leveraging this agent as a means to administer photodynamic therapy (PDT). PDT treatment of the tumor resection cavity can minimize the risk of local reappearance.^10^ PDT involves the photoactivation of a photosensitizer molecule called a photosensitizer, which is selectively incorporated into tumor cells. Light irradiation activates the photosensitizer by transferring energy from the light beam to the sensitizer, resulting after spin flip in the formation of a photosensitizer triplet excited state. This excited state activates nearby dioxygen molecules to produce a massive dose of reactive oxygen species (ROS) that induces cell death.^11^

Though PDT has demonstrated added value for the treatment of GMB patients,^12^ and different forms of PDT are clinically approved for the treatment of tumors such as Barrett’s esophagus or nonmelanoma skin cancer, it has also some limits, including a reduced efficacy in the hypoxic regions of a tumor.^13^ For instance, few studies showed resistance of GBM to PDT when employed in the resection cavity using a cylindrical diffuser fiber after implanting it into the tumor.^13−15^ Another family of molecules called photoactivated chemotherapy (PACT) agents also make use of visible light irradiation to generate high but localized doses of cytotoxic species, leading to lower systemic side effects *in vivo*.^16^ Unlike PDT, PACT activates the prodrug using an O_2_-independent mechanism, including ligand photosubstitution,^17^ covalent bond photocleavage,^18^ or photoisomerization.^19^ Such activation modes lead to changes in the formula of the compound that induces biological damage to irradiated cells, for example, by inhibiting an essential metabolic enzyme. Due to their O_2_-independent mode of activation, a deficiency of dioxygen in the cancer cell does not necessarily affect the activation of PACT compounds, as demonstrated previously.^20,21^ Photocleavable groups based on ruthenium (Ru) are among the most preclinically promising photocages for PACT. While including a second-row transition metal,^22,23^ these prodrugs often show less systemic toxicity than conventional antineoplastic agents based on *e.g.*, platinum.^24^ In addition, their photochemistry is well-understood and finely tunable.^20^ Although new PACT compounds, such as ruthenium-peptide conjugates,^25^ show (photo)toxicity in hypoxic tumor cells and multicellular tumor spheroids (MCTS), PACT has not been applied in the clinic yet.

In principle, ruthenium-based PACT compounds can be used to combat several hallmarks of cancer including uncontrolled proliferation and altered metabolism. Due to their high proliferation rate, cancer cells require a high amount of essential metabolites adenosine triphosphate (ATP) and nicotinamide adenine dinucleotide (NAD^+^). NAD^+^ and its reduced analog NADH are very important electron carriers in cells. Both molecules allow cells to maintain good cellular homeostasis by acting as a substrate for PARP, mono/poly-ADP-ribosylation, and Sirtuin light-activated deacetylation enzymes.^26^ The regulation of NAD^+^ biosynthesis and transport, as well as that of its intermediates, is crucial to sustaining tumor cell growth.^27^ NAD^+^ can be synthesized from various dietary precursor molecules via multiple pathways, but in cancer, NAD^+^ is predominantly produced via the so-called “salvage pathway”.^28^ Since cancer cells require a high amount of NAD^+^ to maintain their functions, multiple researchers have developed therapeutic strategies based on small molecule inhibitors such as FK866 or CHS-828.^29^ These compounds prevent the formation of NAD^+^ by inhibiting nicotinamide phosphoribosyltransferase (NAMPT), an enzyme critical to the NAD^+^ salvage pathway. NAMPT, also called pre-B-cell colony-enhancing factor 1 (PBEF1) or visfatin, is a rate-limiting enzyme for NAD^+^ synthesis that plays an important role in tumor generation and progression.^30,31^ NAMPT is also described as a soluble factor with a cytokine-like activity that regulates cell growth, migration, and gene expression.^32^ Although NAMPT is a promising anticancer drug target, its targeting in patients offers a low therapeutic window, with either a lack of antitumor efficacy at lower doses or too many side effects at higher doses, such as retinal, hematological, or cardiac toxicity.^33^

**Figure 1:**
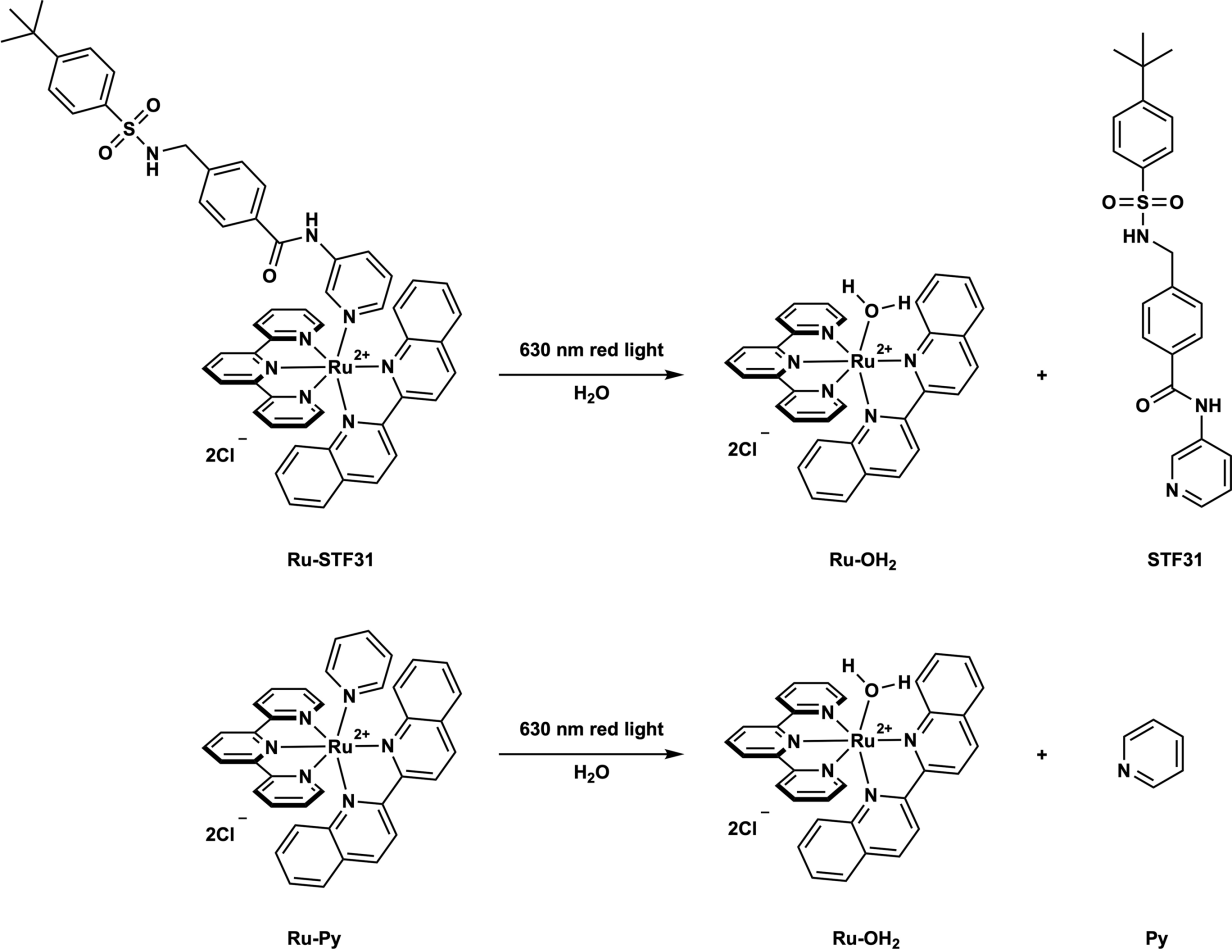
Light activation of the PACT compounds **Ru-STF31** (top) and **Ru-Py** (bottom) by red light. Upon light absorption, both **Ru-STF31** and **Ru-Py** release the activated photocage **Ru-OH**_**2**_, but the former releases the NAMPT inhibitor **STF31**, while the latter releases nontoxic pyridine (**Py**).

To solve this problem, a NAMPT-targeted PACT compound [Ru(tpy)(biq)(**STF31**)]Cl_2_ (**Ru-STF31,** tpy = 2,2’:6′,2″-terpyridine; biq = 2,2′-biquinoline; **STF31** = 4-(((4-(*tert*-butyl)phenyl)sulfonamido)methyl)-*N*-(pyridin-3-yl)benzamide) has been recently developed by our group.^20^ Upon red light irradiation (630 nm), **Ru-STF31** releases both **STF31** and the activated photocage **Ru-OH**_**2**_ (Figure 1, top). **STF31** is a commercially available cytotoxic NAMPT inhibitor that was also shown to influence the glucose transporter GLUT1.^34^**STF31** contains a metal-binding pyridyl group that allows blocking its NAMPT-inhibiting properties by coordination with a ruthenium-based photocaging group. In our original work, **Ru-STF31** was shown to become 2–4 times more cytotoxic upon red light activation *in vitro* in human lung (A549) and nonmelanoma skin (A431) cancer cells, both in normoxia (21% O_2_) and hypoxia (1% O_2_).^20^ Its activity in glioblastoma cells, which forms the core of our new manuscript, had up to now never been reported. In addition, though ruthenium photocages are traditionally believed to play a minor role in the light-triggered activity of PACT compounds, recent works have shown that this assumption may be wrong.^35−37^ In principle, the biological activity of a PACT compound following light irradiation might be a combination of the effect of the released inhibitor and of that of the ruthenium photocage. Given the low amount of information currently available on this question, we addressed it in detail here using the known **Ru-STF31** compound, but in the new context of glioblastoma. We included in this new study the analog compound [Ru(tpy)(biq)(**Py**)]Cl_2_ (**Ru-Py**, Figure 1), a photoactivated ruthenium cage control that releases the same activated cage **Ru-OH**_**2**_ as **Ru-STF31**, together with the biologically benign pyridine but no NAMPT inhibitor (Figure 1, bottom). We first compared NAMPT expression in different cell lines to demonstrate its overexpression in glioblastoma. Second, we measured separately the biological effects of **Ru-STF31** and **Ru-Py** after red light activation and checked how the **STF31** inhibitor may interact, biologically speaking, with the activated cage **Ru-OH**_**2**_. Finally, we compared the antiproliferative effects of these compounds with their ability to modify the NAD^+^ levels in cancer cells using metabolomics, both in a normoxic and hypoxic context, which concluded to a strong influence of the ruthenium caging group on the activity of the photoreleased **STF31** inhibitor, in particular, under hypoxia.

## Results

### Upscaled Synthesis and Crystal Structure of Ru-STF31

**Figure 2:**
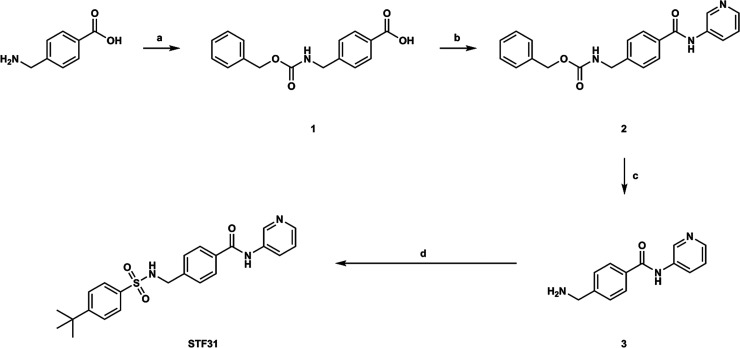
Synthesis of **STF31**. Reaction conditions: (**a**) benzyl chloroformate, NaHCO_3_, H_2_O/dioxane = 6/1, 5 °C to r.t., 18 h, 84%; (**b**) (i) (COCl)_2_, cat. DMF, THF, r.t to 50 °C, 3 h; (ii) 3-aminopyridine, pyridine, r.t., 16 h, 89% overall; (**c**) 33% HBr/AcOH, r.t., 2.5 h, 79%; (**d**) 4-*tert*-butylbenzenesulfonyl chloride, TEA, cat. DMAP, ACN, r.t., 16 h, 83%.

**Figure 3:**
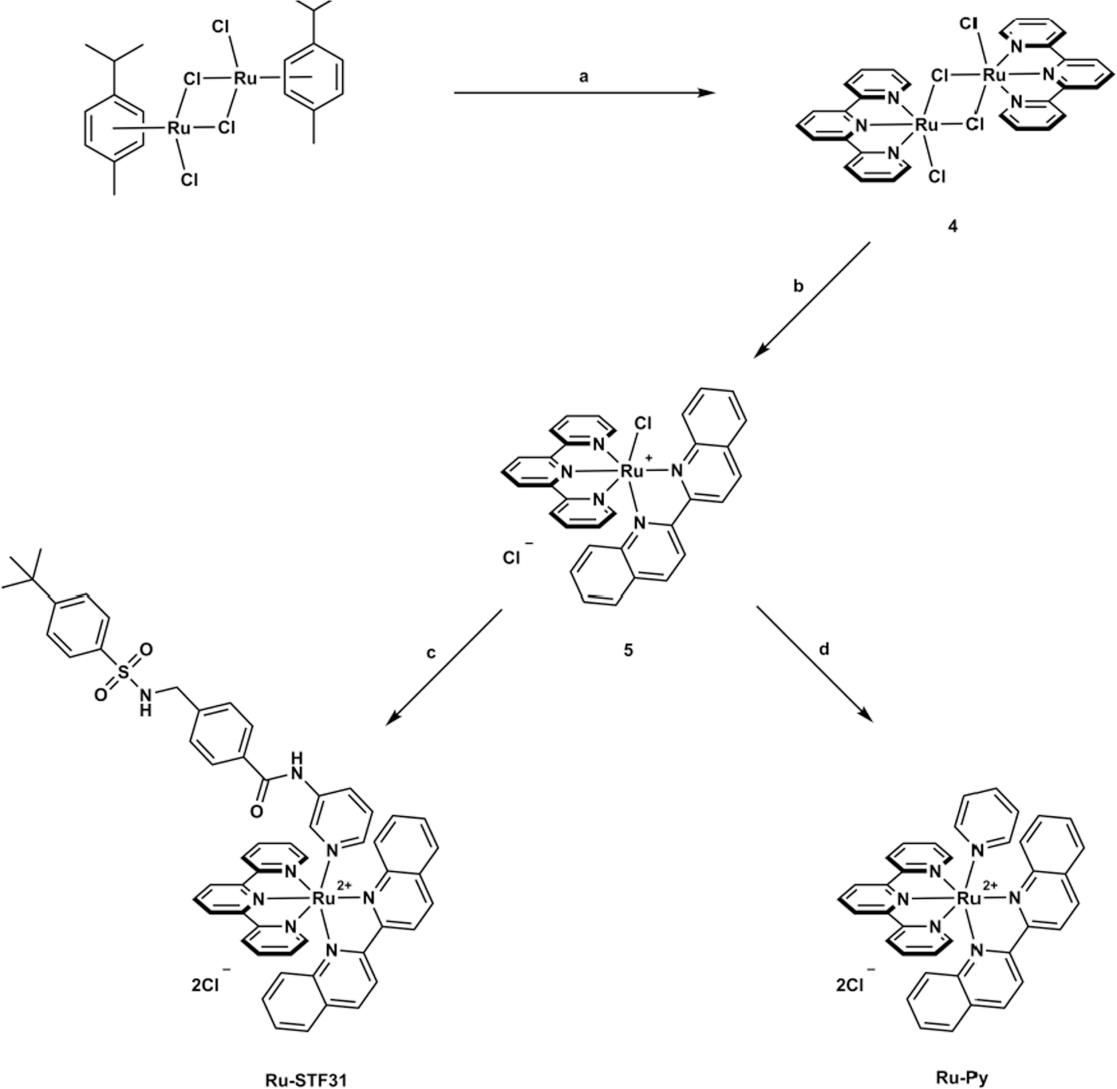
Upscaled synthesis of [Ru(tpy)(biq)(**STF31**)]Cl_2_. Reaction conditions: (**a**) 2,2':6',2''-terpyridine, DCM, r.t., 1.5 h, under N_2_, 79%; (**b**) 2,2′-biquinoline, ethylene glycol, 180 °C, 1 h, under N_2_, 92%; (**c**) (i) **STF31**, AgPF_6_, acetone/H_2_O = 1/1, 50 °C, 16 h, under N_2_; (ii) TBAC, acetone, r.t., 0.5 h, under N_2_, 58% overall; (**d**) pyridine, ethanol/H_2_O = 1/1, 100 °C, 16 h, under N_2_, 89%.

**Figure 4:**
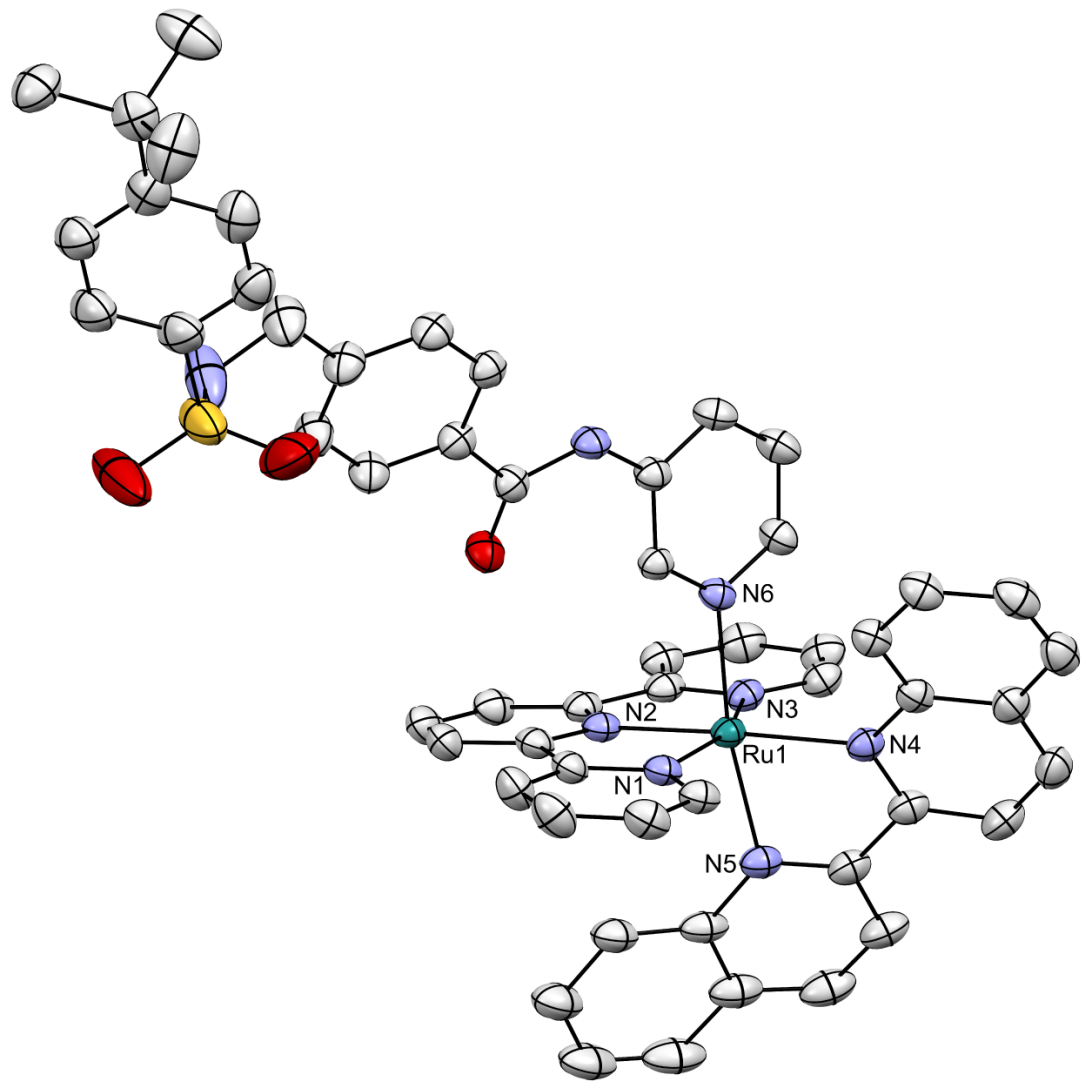
Displacement ellipsoid plot (50% probability level) for the crystal structure of **Ru-STF31** at 110 (2) K. All hydrogen atoms, solvent molecules, and counterions have been omitted for clarity.

As the crystal structure of **Ru-STF31** was unknown, the compound was resynthesized at a larger scale (380 mg, Figures 2,3), and single crystals of [Ru(tpy)(biq)(**STF31**)](BF_4_)_2_ suitable for crystal structure determination (Table S1) were grown by dissolving **Ru-STF31** in MeOH, adding a few drops of the HBF_4_ diethyl ether complex, and waiting for crystallization to occur. The cationic part of the crystal structure is shown in Figure 4, and a selection of bond distances and angles is given in Table S2. This structure unequivocally demonstrated the binding of **STF31** via pyridine to the ruthenium center in the photocaged prodrug **Ru-STF31**. In addition, the biq ligand in the structure was found tilted with respect to the plane perpendicular to the terpyridine ligand, thus generating two enantiomers in the crystal structure. Such a tilt is known to be the result of the steric clash between the pyridyl group of **STF31** and the *ortho* proton of the quinoline fragment situated on the same side. Such steric distortion is also known to generate low-lying triplet metal-centered (^3^MC) excited states that are responsible for the efficient photosubstitution of **STF31** from the ruthenium coordination sphere.^20^

### U87MG Is the Most Suitable Cell Line for Assessing the Efficacy of Ru-Caged NAMPT Inhibitors

**Figure 5:**
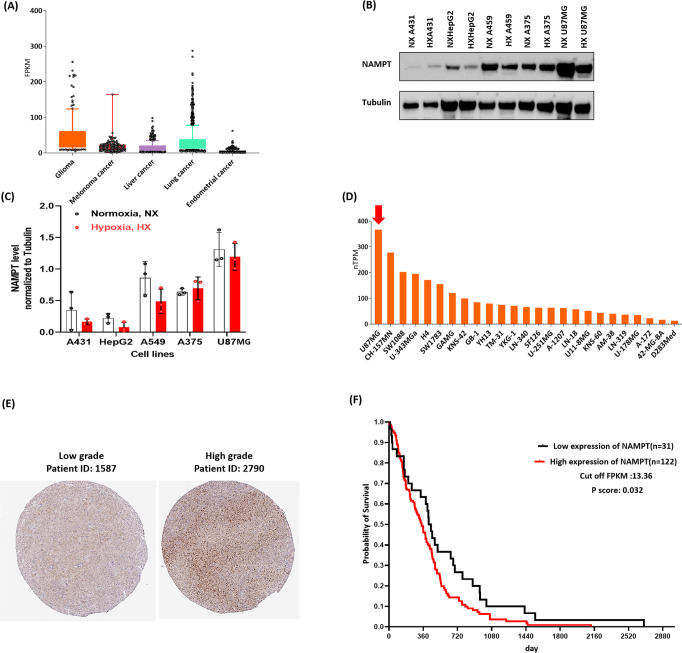
(A) NAMPT RNA-seq data for 5 cancer types from TCGA were analyzed and reported as the median number of fragments per kilobase of exon per million reads (FPKM), reported as median FPKM (https://www.proteinatlas.org/ version 22.0). Normal distribution across the data set was visualized by box plots, shown as median and 25th and 75th percentiles. Points are shown as outliers if they are above or below 1.5 times the interquartile range. (B) Western blotting of NAMPT expression among five different cancer cell lines cultured in both hypoxic (1% O_2_) and normoxic (21% O_2_) conditions showing the highest and lowest NAMPT expression in U87MG and HepG2, respectively. (C) Bar graph of the Western blotting assay, each bar represents a value for the NAMPT protein. (D) Bar graph provided by the Human Protein Atlas version 22.0 and Ensemble version 103.38, showing the highest expressions of NAMPT in U87MG among different brain cell lines [*n* = 25]. (E) Expression of NAMPT detected with the HPA047776 antibody in brain tissue of a patient with high-grade (right tissue) and low-grade (left tissue) GBM provided by Human Protein Atlas (https://www.proteinatlas.org/ version 22.0) showing relatively higher expression of NAMPT in high-grade GBM. (F) A Kaplan–Meier curve was plotted for 153 GBM patients, stratified by high (*n* = 122) and low (*n* = 31) expression levels of NAMPT, using a cutoff of 13.62 FPKM for RNA expression.

NAMPT overexpression was repeatedly reported in many human malignant tumors.^40^ The Human Protein Atlas database (https://www.proteinatlas.org/^200^) (Human Protein Atlas.org, version 22.0 reusing RNA-seq data from The Cancer Genome Atlas (TCGA)) was queried to assess the expression of NAMPT. Figure 5A shows a box plot of pathology data extracted and selected from the Human Protein Atlas. We chose to highlight 5 out of 17 cancer types with a median expression level of 32.6 FPKM in our study (fragments per kilobase of exon per million mapped fragments). According to this analysis, glioma [*n* = 153] turned out as the cancer type with the highest expression of NAMPT at the mRNA level. By contrast, with a median of 4.8 FPKM, endometrial cancer was found as the cancer having the lowest expression of NAMPT [*n* = 541].

### Western Blot Analysis

To validate this clinical observation *in vitro*, we measured NAMPT protein levels by Western blotting in a selection of 5 cell lines representative of different cancers including nonmelanoma skin (A431), liver (HepG2), lung (A549), skin melanoma (A375), and glioblastoma (U87MG). These measurements were performed at two O_2_ concentrations, 21% (hereafter called normoxia) and 1% (hereafter called hypoxia), to assess the influence of oxygen concentration on NAMPT expression. As shown in Figure 5C, within these 5 cancer cell lines, U87MG displayed the highest level of NAMPT protein, independently from the oxygen level. Supposedly, U87MG mostly produces NAD^+^ via the NAD^+^ salvage pathway and hence needs a higher expression of NAMPT as it is a crucial enzyme in this pathway (Sharma *et al.*, 2021). Both HepG2 and A431 cell lines were found to have the lowest NAMPT protein level of this series; the HepG2 cell line was selected in further studies as an NAMPT-negative, low-expression control cell line.

U87MG cells showed high NAMPT expression at both the mRNA and protein levels, but it is not the only brain cancer cell line. To investigate how U87MG expressed NAMPT within other known brain cancer cell lines, we plotted the NAMPT mRNA levels of a large compendium of 25 brain cancer cell lines retrieved from the Human Protein Atlas. Of the 25 brain cancer cell lines panel, we display in Figure 5D the average value of NAMPT mRNA level of 25 cell lines with high, medium, and low expression. Surprisingly, with a value of 362 nTPM (normalized transcripts per million), the U87MG cancer cell line expressed the highest NAMPT mRNA level of the whole series. NAMPT is not only highly expressed in glioblastoma cell lines but also associated in clinics with a worse prognosis for brain cancer patients: a high NAMPT protein level is associated with a higher grade of GBM. From the Human Protein Atlas database, the differential NAMPT expression in high-grade and low-grade GBM from 2 different patients was retrieved (Figure 5E). The higher expression of NAMPT was observed in patients with the highest-grade GBM, which may indicate a possible association between NAMPT levels and the aggressiveness of the disease. Finally, Figure 5F illustrates a Kaplan–Meier curve based on the TCGA database analysis of 153 patients, indicating that higher NAMPT expression is associated with increased mortality among glioblastoma patients compared to those with lower NAMPT expression. Overall, NAMPT appeared clinically as a good target for NAMPT-targeted PACT drugs such as **Ru-STF31**, and U87MG cells were one of the best *in vitro* models tested to test the activity of this PACT agent.

## The Hypoxic Phenotype of U87MG Cells

### Immunohistochemistry of HIF1-α

**Figure 6:**
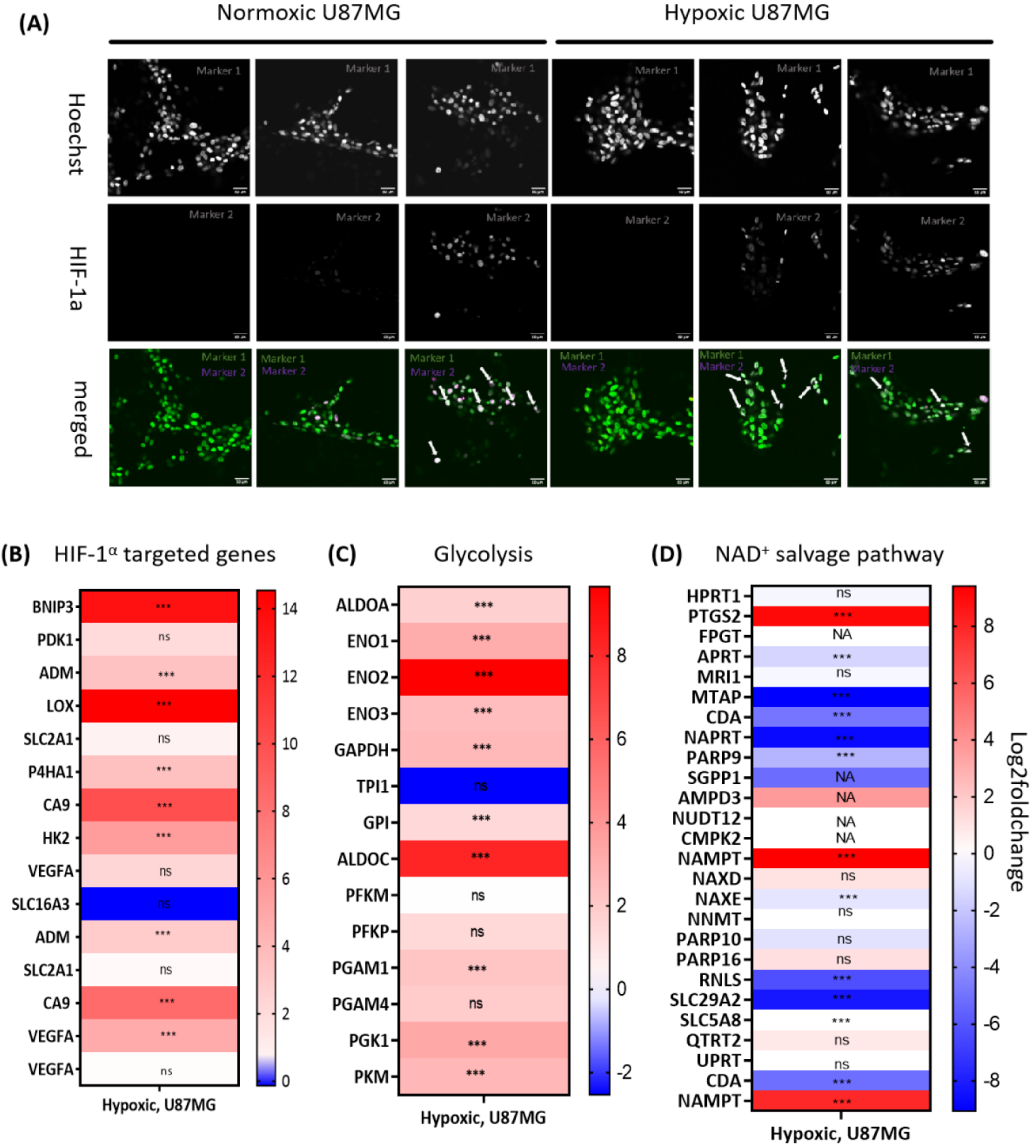
(A) Immunostaining of HIF1-α in U87MG cells cultured in normoxia (21% O_2_) and hypoxia (1% O_2_). Normoxic U87MG cells (left panel) and hypoxic U87MG cells (right panel) were imaged using confocal microscopy. Immunofluorescent staining of HIF1-α is shown in green (middle panel), cell nuclei are stained in blue with Hoechst dye (upper panel), and the merged images are shown in the bottom panel. The control (−) group represents cells with secondary and without primary HIF1-α staining (negative control). The (+) groups represent cells with both primary and secondary HIF1-α staining. Cells treated with CoCl_2_ (100 μM) for 24 h were included as positive control for the expression of HIF1-α. Overexpression and translocation of HIF1-α were observed in both hypoxic U87MG cells and normoxic cells treated with CoCl_2_. Heat map of differentially expressed (B) HIF1-α targeted genes, (C) genes involved in glycolysis, and (D) genes in NAD^+^ salvage pathway, NA: not available values; significant *p*-values are indicated with (***): *p*_adj_ < 0.01, (ns): nonsignificant.

To validate our hypoxic cell culture conditions, we first immunostained HIF1-α in both normoxic (21% O_2_) and hypoxic (1% O_2_) U87MG cells (Figure 6A). As a positive control, we treated normoxic cells with CoCl_2_ (100 μM), a chemical inducer of HIF1-α protein stabilization.^41^ As reported, a low level of HIF1-α protein was observed in normoxic U87MG cells, which was increased upon treatment of the cells with CoCl_2_ (Figure 6A). Furthermore, by incubating the U87MG cells for at least 10 days in hypoxic conditions (1% O_2_), a comparable increased level of HIF1-α was also observed (Figure 6A). The merged images (Figure 6A, bottom) also showed the translocation of HIF1-α into the nucleus, indicated by white arrows. These results validated the stabilization of HIF1-α and its translocation to the nucleus in hypoxic U87MG cells, in response to low oxygen levels.

### Transcriptomics Analysis

We investigated the impact of HIF1-α nuclear colocalization on the hypoxic response in U87MG cells cultured in low oxygen conditions by conducting transcriptomics analysis (Figure 6B–D). Our observations revealed a substantial upregulation not only of the direct target genes of the transcription factor HIF1-α, such as 2 isomers of CA9 (carbonic anhydrase 9) and 1 isomer of VEGFA (vascular endothelial growth factor), showing log2Fold changes of 4.78 and 8.45, respectively, but also in genes contributing to glycolysis, including GAPDH, ALDOA, and ENO1 and 2, with log2Fold changes of 2.63, 1.76, and 3.13, 9.66, respectively (Figure 3B,C). Altogether, these transcriptomics data supported our immunostaining imaging and concluded on activation of the HIF1-α pathway in U87MG cells in our hypoxic (1% O_2_) conditions. Although NAMPT upregulation under hypoxia was not evident at the protein level (Figure 6B), transcriptomics data indicated an increase in NAMPT mRNA levels under low oxygen conditions in U87MG cells. Intriguingly, the analysis revealed a downregulation of numerous genes associated with the NAD^+^ salvage pathway as SLC29A2, and NAPRT in hypoxia (Figure 6D). Overall, hypoxic U87MG cells appear as a more suitable *in vitro* model for testing **Ru-STF31**, notably compared to our previous study using A549 and A431 cells.^20^

### Cellular Toxicity of Ru-STF31 by SRB

**Figure 7:**
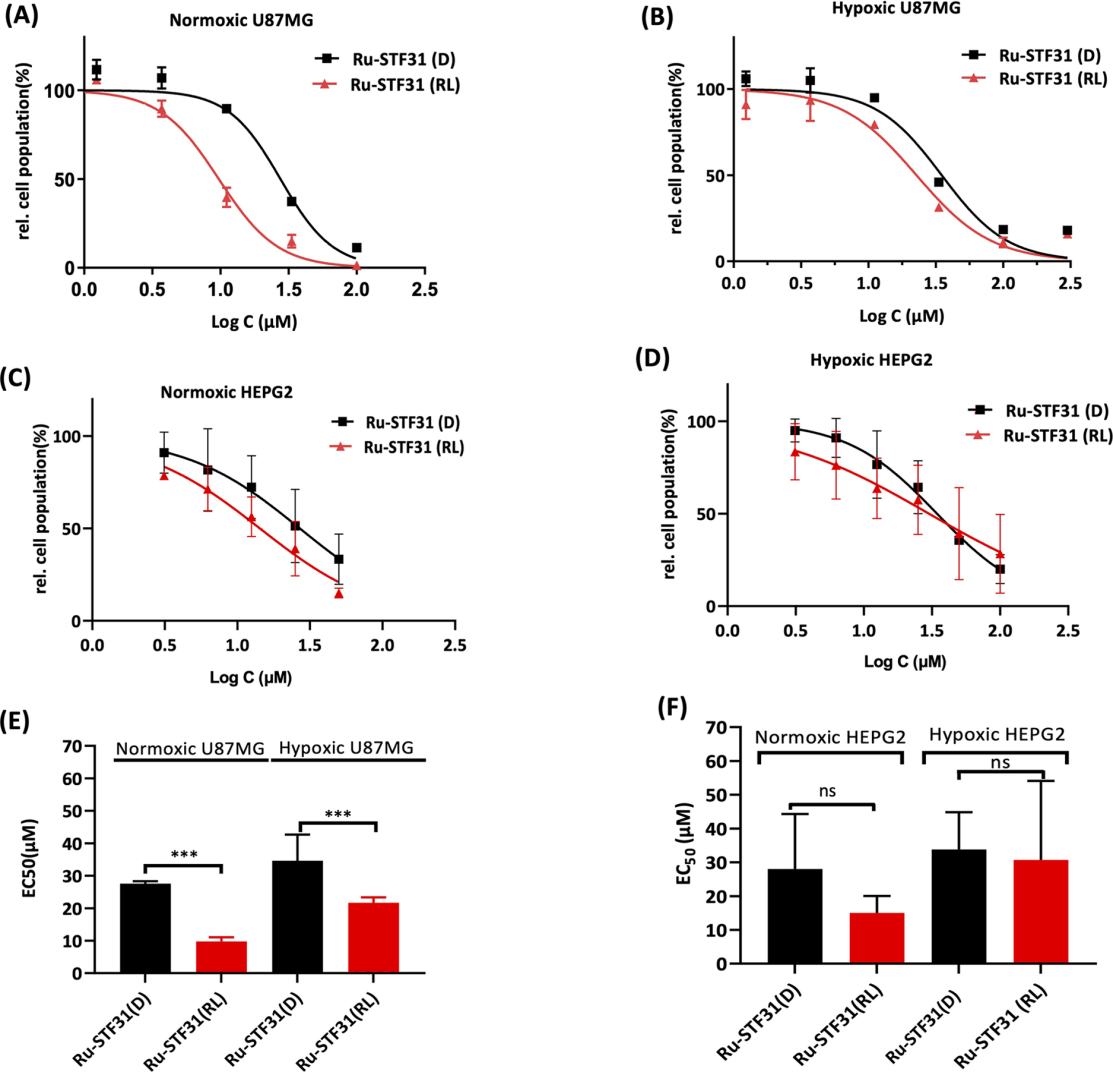
Dose-response curves of **Ru-STF31** for (A) normoxic U87MG cells, (B) hypoxic U87MG cells, (C) normoxic HepG2 cells, (D) or hypoxic HepG2 cells. All cells were treated with **Ru-STF31** and kept in the dark (black data points) or irradiated by red light (630 nm, 21 J/cm^2^, red data points. Data points are averages of three biological replicates (*n* = 3) with 95% confidence intervals (in μM). (E) and (F) Corresponding bar graphs showing cell viability of **Ru-STF31** (D and RL) in U87MG and HepG2 cells, respectively. Statistical significance was assessed via a one-way ANOVA test, (ns): *p* > 0.05, (*): *p* ≤ 0.05, (**): *p* ≤ 0.01, (***): *p* ≤ 0.001, (****): *p* ≤ 0.0001.

Having identified U87MG as a high NAMPT-expressing cell line and HepG2 as a low NAMPT-expressing cell line, we measured the cytotoxicity of the photoactivatable NAMPT inhibitor **Ru-STF31** both in the dark and following red light irradiation in both cell lines (Figure 7). Following exposure to red light irradiation (RL, 630 nm, 21 J/cm^2^), **Ru-STF31** exhibited increased toxicity with EC_50_ values of 9.7 and 22.7 μM in normoxic and hypoxic U87MG cells, respectively, compared to dark conditions (27.8 and 35 μM, Figure 7). The photo index (PI) value of **Ru-STF31**, defined as EC_50_(D)/EC_50_(R), was approximately 2.86 and 1.53, respectively, in normoxic (NX) and hypoxic (HX) U87MG cells, indicating significantly enhanced cytotoxicity under red light irradiation. Under normoxia, lower cytotoxicity was observed for the free inhibitor **STF31** (EC_50_ = 30.2 μM) in U87MG cells in comparison with that of red light-activated **Ru-STF31** (9.7 μM, Table 1). In HepG2 cells, the toxicity of **Ru-STF31** under red light conditions in both NX and HX cells (EC_50_ = 15.0 μM and 30.7 μM, respectively) was slightly lower than that in U87MG cells, but not significantly different from the dark values (EC_50_ =28.0 μM and 33.8 μM, respectively). No significant PI values were observed for **Ru-STF31** in normoxic (1.73) and hypoxic (1.1) HepG2 cells.

**Table 1:** EC_50_ Values of **Ru-STF31** (D, RL), **Ru-Py** (D, RL), and **STF31** in Normoxic and Hypoxic U87MG Human Glioblastoma Cells

compound	parameters	conditions	
		normoxia (21% O_2_)	hypoxia (1% O_2_)
**STF31**	EC_50_(D), μM	30.2	130
	95%CI, μM	+15.3/–9.50	+86.7/–43.8
	EC_50_(RL), μM	-	-
	95%CI, μM	-	-
**Ru-Py**	EC_50_(D), μM	124	133
	95%CI, μM	+31.2/–23.6	+29.8/–23.9
	EC_50_(RL), μM	32.9	44.9
	95%CI, μM	+10.5/–7.58	+22.6/–14.1
	PI(EC_50_(D)/EC_50_(RL))	3.77	2.97
**Ru-STF31**	EC_50_(D), μM	27.8	35.0
	95%CI, μM	+5.91/–4.83	+12.5/–8.35
	EC_50_(RL), μM	9.7	22.7
	95%CI, μM	+1.56/–1.29	+7.35/–5.45
	PI(EC_50_(D)/EC_50_(RL))	2.86	1.53

To investigate the biological effect of the **Ru-OH**_**2**_ cage alone, we also tested the cytotoxicity of **Ru-Py** in the same conditions. Interestingly, cell viability indicated that **Ru-Py** like **Ru-STF31** showed light-dependent cytotoxicity, both under normoxic and hypoxic conditions (Table 1 and Figure 7). Clearly, although the phototoxicity and dark toxicity of **Ru-Py** was lower than that of **Ru-STF31** in both normoxic and hypoxic U87MG cells, the ruthenium photocage showed a biological activity upon light activation that had been overlooked before. In addition, the EC_50_(D) and EC_50_(RL) values of **Ru-Py** were 32.9 and 44.9 μM under normoxia and hypoxia, respectively, which was higher than that of **Ru-STF31** in the same conditions. Overall, according to these results, light-activated **Ru-STF31** had a lower EC_50_ value, both under normoxia and hypoxia, than free **STF31** and light-activated **Ru-Py**, which suggested that both photoreleased fragments **STF31** and **Ru-OH**_**2**_ (Figure 1) might exert a synergistic cytotoxic (or cytostatic) action.

## Impact of Red Light-Activated Ru-Py on the Cytotoxicity of STF31

### Combination Therapy

**Figure 8:**
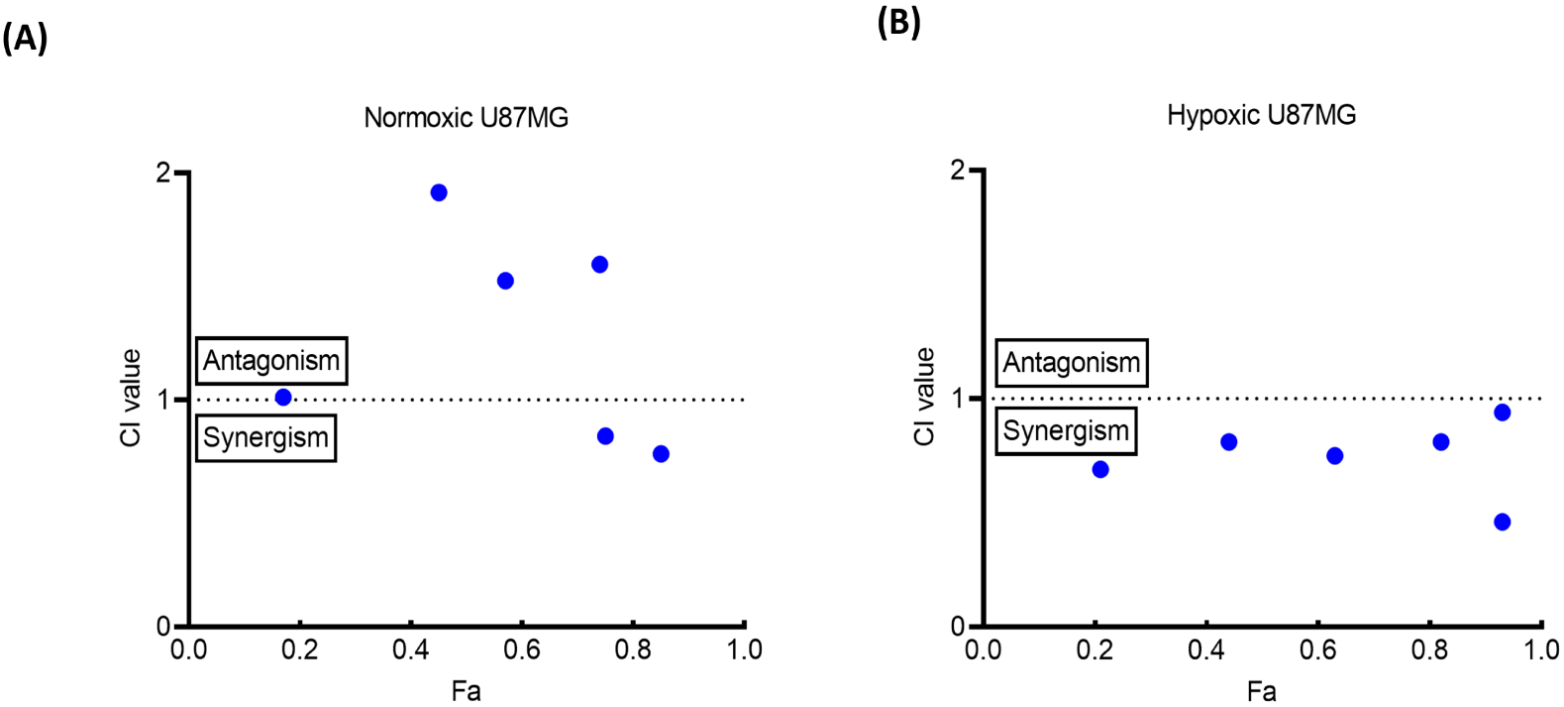
Combination index plot for combination therapy of **Ru-Py** (RL) and **STF31**, in 1:1 concentration ratio in both (A) normoxic (21% O_2_) and (B) hypoxic (1% O_2_) U87MG cells. The *X*-axis represents the fraction affected by the drug (*F*_*a*_), while the *Y*-axis shows the combination index value at each value of *F*_*a*_.

Considering the biological activity of **Ru-Py** upon red light activation, one could wonder whether the ruthenium photocage after light activation of **Ru-STF31** interacts with the photoreleased **STF31** inhibitor. To address this question, we investigated the cytotoxicity of a combination of red light-activated **Ru-Py** (*i.e.*, **Ru-OH**_2_) and free **STF31**, both under normoxia and hypoxia (Figure 8). The combination index (CI) values were computed based on the Chou-Talalay method^42^ using the CompuSyn software (www.combosyn.com). This method is based on the median-effect equation (MEE) of the mass-action law (MAL) expressed in eq 1:
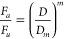
1

where *F*_*a*_ is the fraction affected by the drug (here, cell growth inhibition), *F_u_* is the unaffected fraction (*F*_u_ = 1 – *F_a_*), *D* is the dose of the drug (in μM), *D*_*m*_ is the median-effect dose (in μM) or potency of the drug, and *m* is the slope of the dose–response S-shaped curve. In this formalism, the CI values for each combination and each effect *F*_*a*_ can be calculated by eq 2:
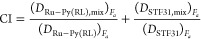
2where  represents the concentration (in μM) of drug *x* in combination to achieve a specific effect *F*_*a*_, and  represents the concentration (in μM) of drug *x* taken alone to achieve the same effect *F*_*a*_ as the combination. The CI provides information about the effect of the combination, compared with an additive effect. CI values lower than 1 indicate synergism, CI = 1 indicates additive effects, and CI values higher than 1 indicate antagonism. All details and the CompuSyn report of **Ru-Py** (RL) and **STF31** including the media effect plot are given in Figure S18.

Strikingly, different CI values were found under normoxia and hypoxia. Under normoxia, at the highest concentrations used where both compounds individually achieved a 75% and 85% effect (*F*_*a*_) on cell death (Figure 8B), CI values lower than 1 were found, corresponding to synergies. However, at lower concentrations, an unexpected antagonistic effect was observed with CI values either close to 1 or higher than 1. In contrast, in hypoxic (HX) cells, a consistent synergy pattern emerged from the data irrespective of the drug concentrations, with all CI values lower than 1 in a 1:1 mixture of **Ru-Py** (RL) and free **STF31**.^43^ Hence, in hypoxic U87MG cells, the photoreleased ruthenium cage **Ru-OH**_**2**_ was found to exert a synergistic influence on the effect of the **STF31** inhibitor itself. To our knowledge, this observation is the first report on the one hand of the fact that the ruthenium cage may influence the biological effects of the photoreleased inhibitor (here **STF31**). On the other hand, these interactions were found dependent on the oxygenation level of the cells, which has been largely overlooked in the past.

## Reduction in Intracellular NAD^+^ Level in Hypoxic (HX) U87MG Cells Treated with Ru-STF31

### Metabolomic Study

**Figure 9:**
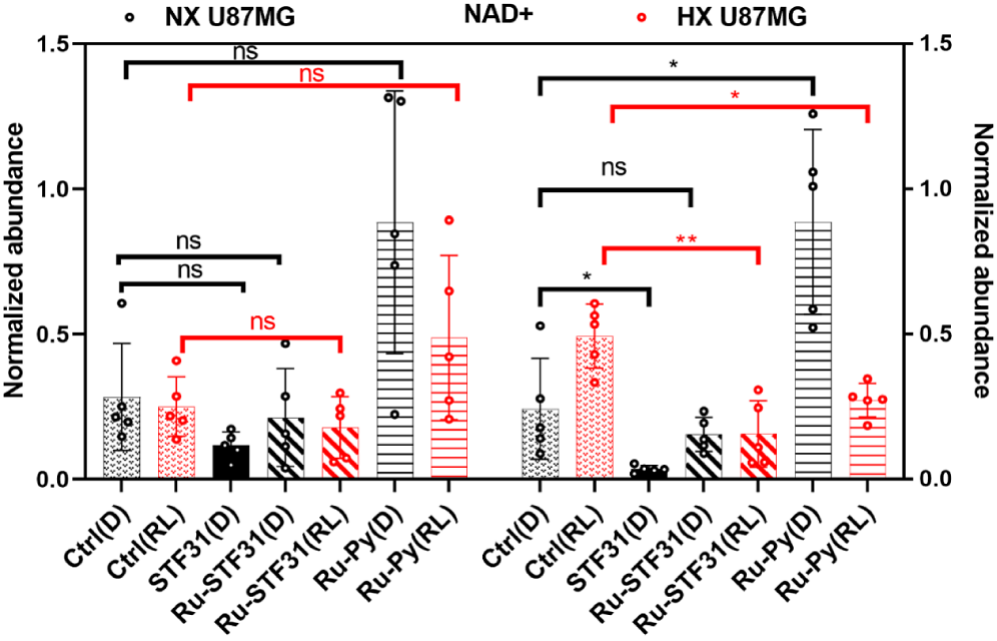
Metabolomics analysis of NAD^+^ level in U87MG cells treated with vehicle control (Ctrl), **STF31**, **Ru-STF31**, or **Ru-Py**, and kept in the dark (D) or irradiated with red light (RL, 630 nm, 21 J/cm^2^). Data points are averages of 5 biologically independent experiments (*n* = 5), and error bars represent 95% confidence intervals. Statistics: paired Student’s *t* test, (ns): *p* > 0.05, (*): *p* ≤ 0.05, (**): *p* ≤ 0.01, (***): *p* ≤ 0.001, (****): *p* ≤ 0.0001.

Based on the cell-free NAMPT enzyme activity assay used in our previous report,^20^ we expected that NAMPT inhibition with light-activated **Ru-STF31** would reduce the intracellular levels of NAD^+^. However, this hypothesis had never been tested in a cellular context. Therefore, we conducted a metabolomic analysis to measure the real intracellular level of NAD^+^ in U87MG cells cultured in different conditions (Figure 9): either untreated (Ctrl) or treated with **Ru-STF31**, **Ru-Py**, or **STF31**, and either kept in the dark (D) or following activation with red light (RL, 21 J/cm^2^).^44,45^ According to this analysis, free **STF31** significantly decreased NAD^+^ levels in hypoxic cells but the change compared with untreated cells was not statistically significant in normoxic cells. In normoxia, **Ru-STF31** in both dark and red light-activated conditions did not affect the amount of NAD^+^; however, in hypoxic cells, light-activated **Ru-STF31** significantly decreased NAD^+^ levels. This observation was consistent with our findings regarding the free inhibitor **STF31**, indicating that treatment with the free NAMPT inhibitor or with its light-activated analog **Ru-STF31** did lead to a reduction in NAD^+^ levels in U87MG cells, but only in hypoxic conditions.

Of course, **Ru-Py** was originally designed as a negative control for NAMPT inhibition. Consequently, it was expected not to have any effect on intracellular NAD^+^ levels. This usual assumption appeared to be correct for normoxic U87MG cells, where **Ru-Py**, whether light-activated or not, did not induce statistically significant differences in the NAD^+^ levels concerning untreated cells. However, it appeared incorrect in hypoxic U87MG cells: **Ru-Py** before activation by red light did increase NAD^+^, and following red light activation and release of **Ru-OH**_**2**_, unexpectedly decreased intracellular NAD^+^ levels. Although it was unclear, at this stage, if **Ru-Py** had any NAMPT inhibitory effect, in hypoxic cells, the ruthenium cage was found to increase NAD^+^ levels whether activated or not. Maybe as a defense mechanism against the toxicity of **Ru-Py**, which seems to disturb cellular hemostasis.^46^

## Rescue Experiments of STF31 and Ru-STF31 with NAD^+^

### Combination Therapy with NAD^+^

**Figure 10:**
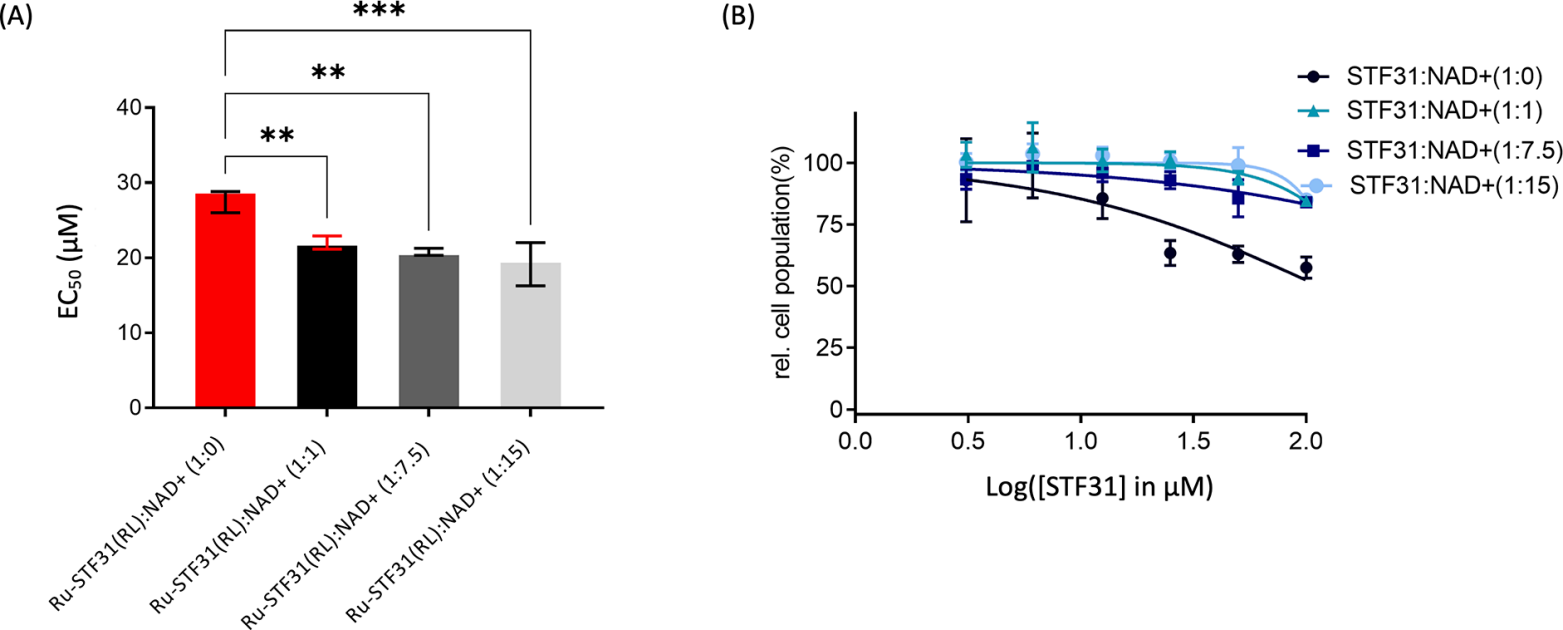
Combination treatment of extracellular NAD^+^ with (A) **Ru-STF31** activated by red light (630 nm, 21 J/cm^2^) or (B) **STF31** in hypoxic U87MG cells. Both drugs were introduced in a concentration range of 100–3.1 μM, and NAD^+^ was introduced in 4 concentration ratios with the drug, *i.e.*, **Ru-STF31**: NAD^+^ or **STF31**:NAD^+^, which were 1:0, 1:1, 1:7.5, or 1:15. Statistical significance was assessed through ordinary one-way ANOVA, (ns): *p* > 0.05, (*): *p* ≤ 0.05, (**): *p* ≤ 0.01, (***): *p* ≤ 0.001, (****): *p* ≤ 0.0001.

In principle, decreasing the intracellular levels of NAD^+^ in U87MG cells by NAMPT inhibitors may be effective and harm the cells if no extracellular NAD^+^ can be taken up by the cancer cells. However, *in vivo*, the tumor microenvironment has the potential to provide critical metabolites like NAD^+^ to promote tumor growth. Therefore, we decided to investigate if adding NAD^+^ in the culture medium of hypoxic U87MG cells treated with red light-activated **Ru-STF31** or **STF31** would rescue them or not, hence lower toxicity of the inhibitor. To this aim, three different ratios between the ruthenium drug and NAD^+^ were investigated, *i*.*e.*, 1:1, 1:7.5, and 1:15. For instance, for the 1:1 ratio, hypoxic U87MG cells were treated with **Ru-STF31** or **STF31** in a concentration range of 100–3.1 μM. Following photoactivation of **Ru-STF31**, which released **STF31**, or in the dark for **STF31**, NAD^+^ was added to the cells at the same concentration as the drug (concentration range of 100–3.1 μM). For free **STF31**, the toxicity of the drug toward U87MG cells was as expected reduced at all drug:NAD^+^ ratios (Figure 10B). This result supported the hypothesis that the presence of additional NAD^+^ in the culture medium “rescued” the toxic effects of the NAMPT inhibitor **STF31**. In other words, free **STF31** and NAD^+^ acted antagonistically for cell killing. For light-activated **Ru-STF31**, however, a reverse effect was obtained, *i.e.*, the EC_50_ value of the ruthenium PACT compound after red light activation was decreased when the treatment was combined with various concentration ratios of NAD^+^ (1:1, 1:7.5, or 1:15, Figure 10A). This unexpected result suggested that a synergistic effect occurred, in toxicity terms, when red light-activated **Ru-STF31** was combined with NAD^+^. In conclusion, in hypoxic U87MG cells, the toxicity resulting from red light-activated **Ru-STF31** could not be rescued by adding additional NAD^+^. On the contrary, synergistic cytotoxic effects were observed at all ratios of the combined treatment, suggesting that extracellular NAD^+^ provided by the tumor microenvironment may even increase the effect of the PACT prodrug in hypoxic regions after light activation. Though it is unclear, at this stage, where this phenomenon comes from, our data highlight that the simple picture of light-activated inhibitors targeted to NAMPT is, in fact, more complicated than initially expected.

## Discussion

In our study, it first appeared that U87MG glioblastoma cells serve as a highly appealing *in vitro* model for testing photoactivated NAMPT inhibitors such as **Ru-STF31**. Our results suggested that the correlation between NAMPT expression and glioma grading aligned with previous studies^47,48^ and supported the idea that NAMPT may play a role in glioma aggressiveness and progression. This insight also highlighted that NAMPT-targeted light-activated therapies are promising strategies for managing high-grade gliomas more effectively. Moreover, we showed that when culturing U87MG cells in hypoxic conditions (1% O_2_) for at least 10 days, stabilization of HIF1-α and nuclear translocation occurs, leading to substantial upregulation of genes associated with the HIF1-α pathway. Hypoxic U87MG cells may hence represent a good *in vitro* model for hypoxic regions of glioma tumors. Following this hypothesis, we assessed the impact of NAMPT expression levels on the efficacy of the photoactivated NAMPT inhibitor **Ru-STF31** using U87MG cells as a model of the cell line with high NAMPT expression, and HepG2 as a control cell line with low NAMPT expression. Despite expecting higher toxicity in U87MG, we were aware of the potential development of resistance mechanisms in cells with elevated expression of the target protein,^49^ or in cells with low O_2_ concentrations.^6^ These adaptive responses could both diminish the effectiveness of **Ru-STF31**. We observed significantly increased toxicity of **Ru-STF31** in red light conditions in U87MG (9.7 μM) compared to HepG2 (21.6 μM), highlighting its more potent impact on high NAMPT-expressing cells while comparable toxicity was found in the dark in both cell lines. On paper, the expression level of NAMPT should be crucial for the effectiveness of **Ru-STF31** because it targets NAMPT following light activation: higher expression levels of NAMPT in U87MG cells should likely result in greater binding and inhibition by **Ru-STF31**, leading to increased cytotoxicity upon light activation. In addition, the absence of significant PI values in low NAMPT-expressing HepG2 cells suggested that the absence of effectiveness for **Ru-STF31** in this cell line may be directly related to its low NAMPT expression level. According to these results, the biological results seemed to follow the design of the molecule.

On the other hand, we realized that the lower toxicity of our compound in the hypoxic U87MG cell line compared to normoxic ones did not follow the expected pattern. STF31 was also described as a GLUT1 inhibitor, and we wondered whether the influence of O_2_ on the activity of the activated drug under hypoxia may be attributed to other factors like differential glycolysis^50^ which we observed in our transcriptomics analysis for glycolysis differentially expressed genes like GAPDH, ALDOA, and ENO1 and 2. These genes play a crucial role in glycolysis and could contribute to the observed changes in the cellular toxicity of our compounds. These doubts prompted further study on the mechanisms underlying cell death in U87MG cells.

The observed patterns of synergy or antagonism in the combination study of red light-activated **Ru-Py** and free **STF31** in hypoxic and normoxic U87MG cells present important insight into the effects of oxygen on the biological effects of PACT prodrugs. The synergy, which was observed in hypoxia at a 1:1 concentration ratio, suggested that the presence of red light-activated **Ru-Py** (**Ru-OH_2_** in Figure 1) in hypoxic cells also containing free **SFT31** enhanced the toxic effects of the organic NAMPT inhibitor. Such enhancement may come from complementary mechanisms or pathways that are more pronounced under low oxygen levels. For instance, our metabolomics data indicate an unexpected decrease in NAD^+^ levels in hypoxia in the presence of light-activated **Ru-Py**, which is quite similar to the NAD^+^-lowering effects of the free inhibitor **STF31** in the same conditions. Such effects of the ruthenium cage were certainly not expected when designing **Ru-STF31**, but they fit well with the observed synergistic cytotoxicity between light-activated **Ru-Py** and free **STF31**. The significant reduction in NAD^+^ levels under hypoxia indicated a shared therapeutic effect of both fragments obtained after light activation of **Ru-STF31**, specifically in lowering intracellular NAD^+^ levels. Such a decrease in NAD^+^ levels in a hypoxic microenvironment is noteworthy as NAD^+^ is a crucial coenzyme involved in various cellular processes. Overall, the observed synergy between **Ru-OH_2_** and **STF31** in reducing NAD^+^ levels could indicate a promising way for therapeutic mediation, particularly in hypoxic tumor regions where single treatments may be less effective.^51^

On the other hand, the unexpected antagonistic effect observed at lower concentrations in normoxic U87MG cells, and the overall concentration-dependent synergies observed in our combination study, indicated a more complex interaction between **Ru-OH_2_** and **STF31** in an oxygen-rich environment. This second main result of our study suggested that the interplay between both photoreleased fragments of **Ru-STF31** following light irradiation may vary based on the cellular context. It also highlights the importance of seriously probing oxygen^52^ levels in tumor tissues when designing combination therapies or PACT treatment with compounds such as **Ru-STF31**. Recent studies have reported that NAMPT inhibitors may synergize with DNA damage-inducing chemotherapeutics,^40^ but these studies did not consider the potential influence of dioxygen. Usually, investigations on the synergies between different drugs focus on the molecular and cellular mechanisms targeted by these drugs. Our observations on the important role of O_2_ in the activation of **Ru-STF31** may prompt a revision of existing combination therapies involving NAMPT inhibitors, urging researchers and clinicians to consider the oxygen status of a tumor as an additional environmental variable influencing drug–drug interactions or interactions between drug fragments, here those obtained by photochemical cleavage of the coordination bond between ruthenium and the NAMPT inhibitor **STF31**.

While **STF31** appears toxic to cells, according to metabolomics, it did not significantly affect NAD^+^ levels in normoxic conditions, suggesting that its toxicity may be due to effects other than NAMPT inhibition. It is worth noting that **STF31** has been reported as an inhibitor of the glucose transporter 1 (GLUT1).^53^ GLUT1 inhibition by **STF31** and DNA methylation by Temozolomide showed synergistic effects in glioblastoma.^54^ Considering the known DNA-binding abilities of ruthenium complexes with open positions in their coordination sphere,^55^ the higher PI value in normoxia might result from off-targets, such as GLUT1 inhibition and/or DNA binding of **Ru-OH_2_**. In other terms, the phototoxicity of **Ru-STF31** under normoxia might be unrelated to NAMPT inhibition. Under hypoxia, GLUT1 overexpression might take place, rendering this mechanism irrelevant, while DNA damage may be modified by the different conditions, overall leading to lower phototoxicity after red light activation, and hence lower photo index. In such conditions, the selective reduction in NAD^+^ levels by **Ru-STF31** after light activation may explain the remaining effect of the activated drug. Combining **Ru-STF31** (RL) with other drugs that act specifically in hypoxic conditions, such as autophagy inhibitors like chloroquine or hydroxychloroquine, holds promise for enhancing hypoxic cancer therapy. Overall, the unexpected effects of **Ru-Py** challenge our initial assumption that it would not impact NAD^+^ levels. The observed increase in NAD^+^ levels before activation and the subsequent decrease after activation by red light suggest complex cellular responses to simple ruthenium complexes such as **Ru-Py.** These responses may involve adaptive mechanisms or compensatory pathways.

Though synergies were identified by comparing one vs mixtures of two drugs **Ru-OH**_**2**_ and **STF31** in hypoxic cells, which is usually interpreted as a good thing as it results in low EC_50,RL_ for light-activated **Ru-STF31**, one should not ignore the disappointingly low photo index found for this compound under hypoxia (PI = 1.5), which make it a not very successful light-activated drug in such conditions. Such low PI values must hence be reinterpreted as a consequence of a comparatively low EC_50,D_, and hence high toxicity for **Ru-STF31** in hypoxic dark conditions. Indeed, the EC_50,D_ values of **STF31**, **Ru-Py**, and **Ru-STF31** were 130, 44.9, and 35.0 μM, respectively, which highlighted that the “prodrug” **Ru-STF31** was, in the dark, much more cytotoxic than the free inhibitor **STF31**, and a bit more toxic than the nonactivated ruthenium cage **Ru-Py**. In normoxia, the photo index of **Ru-STF31** was significantly higher (2.9) than in hypoxia despite the absence of clear-cut synergies between **Ru-OH**_**2**_ and **STF31** after light activation, simply because the EC_50,D_ values of free **STF31**, **Ru-Py**, and **Ru-STF31** were 30.2, 124, and 27.8 μM, respectively. In other words, under normoxia, and in the dark, **Ru-STF31** was not much more cytotoxic than **STF31**, which contributed to a higher PI value. The existence of synergies after light irradiation should hence not be overinterpreted in terms of molecular design, nor should it be ignored that light-activated compounds should in principle maximize their photo index to offer low toxicity in the dark and high toxicity after activation.

Overall, targeting NAMPT inhibition for anticancer therapy showed a stunning lack of clinical success considering the link between NAMPT expression and tumor outcome. In other words, it has been difficult to find a good therapeutic window for such compounds. In our hands, the addition of NAD^+^ to hypoxic U87MG cells treated with free **STF31** demonstrated a reduction in the toxicity of the NAMPT inhibitor, supporting the idea that external NAD^+^ could moderate the adverse effects of NAMPT inhibition. These results suggested that the presence of extracellular NAD^+^ in the tumor microenvironment may rescue NAMPT inhibition and intracellular NAD^+^ depletion in the tumor cancer cells.^48 ,56,57^ In glioblastoma, extracellular NAD^+^ can be transported inside cells by membrane transporters named SLC (solute carrier family) or by gap junction with neighboring cells. Also, proteins such as connexin 43 hemichannels or CD38 may act as extracellular shuttles to transport NAD^+^.^54^ Reduced efficacy of NAMPT inhibitors against tumors requires higher concentrations of the drug, which harms the patient. An unexpected outcome of our work emerged when combining red light-activated **Ru-STF31** with extracellular NAD^+^. Unlike the anticipated rescue effect, a toxicity increase was observed, as evidenced by a decrease in the EC_50_ value of light-activated **Ru-STF31**. This unexpected interaction challenges the simplistic view of PACT compounds as simple light-activatable protein inhibitors and underscores the complex nature of cellular responses to treatments involving ruthenium photocages. More studies are needed to understand the reason(s) behind such interactions, and their consequence on antitumor efficacy of ruthenium-based PACT compounds *in vivo*, also as drug transporters, *i.e.*, in the absence of light activation. Overall, these observations highlight the complex interaction between oxygen concentration, NAD^+^ metabolism, and the cytotoxic mechanisms of **Ru-Py**, **Ru-STF31**, and free **STF31**. While oxygen concentration appears to significantly influence the cytotoxicity of **Ru-STF31** and free **STF31**, the role of NAMPT inhibition varied for the different compounds and remained significant mostly under hypoxic conditions.

## Conclusion

In conclusion, our study on the efficacy of the photoactivated NAMPT inhibitors **Ru-STF31** using U87MG cells as an *in vitro* tumor model has provided valuable insights into the potential of ruthenium-based PACT treatment of high-grade gliomas. The correlation observed between NAMPT expression and glioma grading aligns with previous studies, highlighting the view that NAMPT plays a crucial role in glioma aggressiveness. Under hypoxic conditions, the upregulation of genes associated with the HIF1-α pathway in U87MG cells emphasizes their shared hypoxic features with real GBM tumors, confirming their appropriateness as an *in vitro* experimental platform. The impact of **Ru-STF31** on high NAMPT-expressing U87MG cells, coupled with its unexpected interaction with NAD^+^, suggested new possibilities for using ruthenium compounds as hypoxia-targeted cancer therapies.

On the other hand, the drug- and O_2_-concentration dependence of the biological interactions between the activated ruthenium photocage **Ru-OH**_**2**_ and the inhibitor **STF31** released by red light activation of **Ru-STF31** underscores the potentially important biological effects of photoreleased ruthenium-based caging groups in Ru-based PACT. Our study demonstrates that it is possible to observe opposing performance indicators for PACT compounds: clear-cut synergistic effects between the photoreleased inhibitor and cage, but a disappointingly low photoindex due to the high dark toxicity of the prodrug. It also highlights the importance of considering the tumor microenvironment and the O_2_ status when studying combination therapies, at least for the treatment of glioblastoma. Additionally, the probable existence of off-targets suggests that ruthenium-based PACT complexes such as **Ru-STF31** might behave in a more complex fashion than as simple light-activatable protein inhibitors targeted “by design” to NAMPT. For example, the increased toxicity when combining NAD^+^ and **Ru-STF31** in the dark highlighted the complex dynamics involved in the cellular responses to treatment with ruthenium complexes, which might also explain the low photo index of this PACT compound, in particular, under hypoxia. Overall, despite the mechanistic challenges revealed by this work, our findings provide valuable insights for refining NAMPT-targeted therapeutic strategies against glioblastoma. They urge for the development of new PACT compounds characterized by lower dark toxicity of the prodrug and synergistic action of the ruthenium-containing cage and the organic inhibitor after light activation, and this both under normoxia and hypoxia.

## Experimental Section

### Solvents and Materials

Solvents used in the metabolomics study including acetonitrile and methanol in LC-MS grade and chloroform in HPLC grade were purchased from Biosolve BV (The Netherlands). Milli-Q ultrapure water was obtained from a Merck Millipore A10 purification system (Raleigh, USA). Ammonium formate was purchased from Sigma-Aldrich (St. Louis, USA). The ^13^C- and ^15^N-labeled amino acids and organic acids (U–^13^C_4_, U-D_3_, 9-^15^N-aspartate, U–^13^C_5_-glutamine, 2,3,3-D_3_-leucine, 1-^13^C, ^15^N-isoleucine, U–^15^N_2_-UMP, ^13^C_3_-pyruvate, 2,2,3,3-D_4_-succinate, 2,2-D_2_-glycine, 2,3-D_2_-fumarate, U–^13^C_11_, U–^15^N_2_-tryptophan, U–^13^C_4_, U–^15^N_2_-asparagine, U–^13^C_5_, U-D_5_, ^15^N-glutamate, U–^13^C_5_-valine, U–^13^C_6_-lysine, ^13^C_3_-lactate, and 2,2,3,3-D_4_-alanine) were purchased from Sigma-Aldrich and Cambridge Isotope Laboratories (Tewksbury, MA, USA).

All cells were cultured in Dulbecco’s Modified Eagle Medium (DMEM) with 10% fetal calf serum (FCS), 2 mM l-glutamine, and 0.2% Pen-Strep (100 mg/mL penicillin + 100 mg/mL streptomycin). The internal standard mix solution was prepared by mixing each standard stock solution (10 mM in Milli-Q water) and stored at −80 °C.

Cells were cultured in 25 or 75 cm^2^ flasks under humidified conditions, at 37 °C, 5% CO_2_, and 21% or 1% O_2_ for normoxia or hypoxia, respectively. Upon reaching 70–80% confluence, the cultures were split and cultured in a new flask.

Unless otherwise noted, the reactions were carried under air at room temperature (r.t.) using common laboratory glass equipment. All reagents and solvents were purchased from commercial suppliers (BLDPharm, AmBeed, Fluorochem, Sigma-Aldrich, Acros, Alfa Aesar, VWR, and TCI) and used as obtained. The standard Schenk technique was used to carry reactions under nitrogen atmosphere when needed. Thin layer chromatography (TLC) was performed using Supelco analytical silica gel on aluminum foil with a fluorescent indicator of 254 nm. Column chromatography was carried out using either silica gel (40–63 μm) or Sephadex LH-20 for size exclusion (SEC) and driven by pressurized air. The columns were packed using a slurry method, and the compounds were introduced in the form of either a solution or a mixture with silica gel. Nuclear magnetic resonance (NMR) spectra were recorded on Bruker Avance 400 or 500 MHz and processed with MestReNova software. The chemical shifts are given relative to the residual signal of the solvent (DMSO-*d*_6_: δ(^1^H) = 2.50; δ(^13^C) = 39.52; CD_3_OD: δ(^1^H) = 3.31; δ(^13^C) = 49.00).^38^ The regular mass spectra (ESI-MS) were recorded on Shimadzu LCMS-2020 (ESI-Q). The elemental analysis was done by Mikroanalytisches Laboratorium Kolbe (MIKROLAB). **STF31**, [Ru(tpy)(biq)(**STF31**)]Cl_2_ (**Ru-STF31**), and [Ru(tpy)(biq)(**Py**)]Cl_2_ (**Ru-Py**) were synthesized by procedures modified from the literature.^20^ The purity of these three titled compounds was above 95% as analyzed by elemental analysis and HPLC.

### Synthesis and Characterization

#### Synthesis of 4-((((Benzyloxy)carbonyl)amino)methyl)benzoic Acid (1)

Benzyl chloroformate (5.77 mL, *d* = 1.20 g/mL, 39.60 mmol, 1.23 equiv) was added dropwise to an ice-bath-cooled mixture of 4-(aminomethyl)benzoic acid (5.00 g, 32.10 mmol, 1 equiv) and NaHCO_3_ (2.70 g, 32.10 mmol, 1 equiv) in 350 mL of H_2_O/dioxane = 6/1. Upon addition, the reaction was stirred for 18 h at r.t. The resulting suspension was filtered, and the filter cake was washed with water and then Et_2_O. Drying *in vacuo* afforded compound **1** as a white powder (7.71 g, 27.00 mmol, 84%). ^1^H NMR (400 MHz, DMSO-*d*_6_) δ 12.90 (s, 1H), 7.96–7.86 (m, 3H), 7.41–7.27 (m, 7H), 5.05 (s, 2H), 4.27 (d, *J* = 6.2 Hz, 2H). ^13^C NMR (101 MHz, DMSO-*d*_6_) δ 167.22, 156.47, 144.94, 137.12, 129.43, 129.34, 128.42, 127.88, 127.82, 127.02, 65.52, 43.64.

#### Synthesis of Benzyl (4-(Pyridin-3-yl-carbamoyl)benzyl)carbamate (2)

Compound **1** (7.71g, 27.00 mmol, 1 equiv) was dissolved in 108 mL of dried THF at r.t., and a few drops of DMF were added as a catalyst. Then, the (COCl)_2_ (3.85 mL, *d* = 1.50 g/mL, 1.65 equiv) was added dropwise, and the mixture was stirred until the end of bubbling. The obtained solution was stirred for 3 h at 50 °C until reaction completion was verified by NMR in CDCl_3_. The resulting mixture was concentrated *in vacuo*, and the residue was dissolved in 60 mL of dried pyridine at r.t. followed by the addition of 3-aminopyridine (2.70 g, 28.40 mmol, 1.06 equiv) in portions. The resulting light orange suspension was stirred for 16 h at r.t., upon which the reaction was quenched with water. The obtained precipitate was filtered off, washed with water and Et_2_O, and then dried *in vacuo* to afford compound **2** as a white powder (8.69 g, 24.05 mmol, 89%). ^1^H NMR (500 MHz, DMSO-*d*_6_) δ 10.41 (s, 1H), 8.93 (d, *J* = 2.5 Hz, 1H), 8.31 (dd, *J* = 4.7, 1.5 Hz, 1H), 8.19 (ddd, *J* = 8.4, 2.6, 1.5 Hz, 1H), 8.03–7.84 (m, 3H), 7.47–7.17 (m, 8H), 5.06 (s, 2H), 4.30 (d, *J* = 6.2 Hz, 2H). ^13^C NMR (101 MHz, DMSO-*d*_6_) δ 165.73, 156.45, 144.54, 143.89, 141.96, 137.14, 135.86, 132.88, 128.42, 127.88, 127.86, 127.81, 127.31, 126.92, 123.56, 65.50, 43.60.

#### Synthesis of 4-(Aminomethyl)-*N*-(pyridin-3-yl)benzamide (3)

Compound **2** (8.69 g, 24.05 mmol, 1 equiv) was mixed with 33% HBr/AcOH (130 mL, *d* = 1.35 g/mL, 0.72 mol) and stirred for 2.5 h at r.t. Upon reaction completion, the Et_2_O was added to a mixture and the resulting precipitate was filtered off. The filter cake was washed with Et_2_O and dried *in vacuo* yielding hydrobromide of the target product as a white solid. ^1^H NMR (500 MHz, DMSO-*d*_6_) δ 11.15 (s, 1H), 9.46–9.37 (m, 1H), 8.76 (d, *J* = 8.2 Hz, 1H), 8.71–8.64 (m, 1H), 8.34 (s, 3H), 8.11 (dd, *J* = 8.4, 2.2 Hz, 2H), 8.07–8.00 (m, 1H), 7.68 (dd, *J* = 8.5, 2.0 Hz, 2H), 4.17 (q, *J* = 5.9 Hz, 2H). This powder was then dissolved in ice-bath-cooled water followed by the addition of solid NaOH in portions. Once basic pH was reached, the addition was stopped, and the resulting precipitate was filtered and washed with water. Drying *in vacuo* afforded compound **3** as an off-white powder (4.32 g, 19.01 mmol, 79%). ^1^H NMR (500 MHz, DMSO-*d*_6_) δ 10.38 (s, 1H), 8.93 (dd, *J* = 2.5, 0.7 Hz, 1H), 8.30 (dd, *J* = 4.7, 1.5 Hz, 1H), 8.20 (ddd, *J* = 8.3, 2.6, 1.5 Hz, 1H), 7.96–7.89 (m, 2H), 7.53–7.46 (m, 2H), 7.39 (ddd, *J* = 8.3, 4.7, 0.8 Hz, 1H), 3.80 (s, 2H), 1.88 (s, 2H). ^13^C NMR (126 MHz, DMSO-*d*_6_) δ 165.83, 148.61, 144.50, 142.00, 135.93, 132.14, 127.65, 127.30, 126.95, 123.55, 45.37.

#### Synthesis of STF31

Compound **3** (1.00 g, 4.40 mmol, 1 equiv) was mixed with DMAP (5.43 mg, 0.04 mmol, 0.01 equiv) and TEA (0.68 mL, *d* = 0.73 g/mL, 4.84 mmol, 1.1 equiv) in 22 mL of dried ACN at r.t. The resulting suspension was stirred for 5 min followed by the addition of 4-*tert*-butylbenzenesulfonyl chloride (1.10 g, 4.62 mmol, 1.05 equiv) in portions. Upon addition, the mixture became a transparent orange solution for a couple of minutes and then precipitation occurred. The resulting suspension was stirred for 16 h at r.t. and then concentrated *in vacuo*. The aqueous NaHCO_3_ saturated solution was added to obtain a crude solid, and once basic pH was reached, the precipitate was filtered and washed with water and Et_2_O. Drying *in vacuo* afforded the target compound as a beige powder (1.55 g, 3.66 mmol, 83%). Purity was >95%. ^1^H NMR (500 MHz, DMSO-*d*_6_) δ 10.38 (s, 1H), 8.96–8.87 (m, 1H), 8.31 (dd, *J* = 4.7, 1.5 Hz, 1H), 8.22 (t, *J* = 6.4 Hz, 1H), 8.17 (ddd, *J* = 8.3, 2.6, 1.5 Hz, 1H), 7.89–7.83 (m, 2H), 7.72–7.66 (m, 2H), 7.57–7.52 (m, 2H), 7.42–7.35 (m, 3H), 4.09 (d, *J* = 6.4 Hz, 2H), 1.28 (s, 9H). ^1^H NMR (500 MHz, CD_3_OD) δ 8.87 (dd, *J* = 2.5, 0.8 Hz, 1H), 8.31 (dd, *J* = 4.8, 1.5 Hz, 1H), 8.24 (ddd, *J* = 8.4, 2.6, 1.5 Hz, 1H), 7.86–7.81 (m, 2H), 7.75–7.69 (m, 2H), 7.56–7.51 (m, 2H), 7.45 (ddd, *J* = 8.4, 4.8, 0.8 Hz, 1H), 7.40–7.34 (m, 2H), 4.17 (s, 2H), 1.33 (s, 9H). ^13^C NMR (126 MHz, DMSO-*d*_6_) δ 165.52, 155.32, 144.58, 142.01, 141.81, 137.86, 135.81, 132.92, 127.65, 127.49, 127.34, 126.37, 125.97, 123.55, 45.79, 34.81, 30.79. ESI-MS: exact *m*/*z* calculated for [C_23_H_25_N_3_O_3_S+H]^+^: 424.2; found: 424.3. Elemental analysis calculated for C_23_H_25_N_3_O_3_S (%): C, 65.23; H, 5.95; N, 9.92; found: C, 65.03; H, 5.97; N, 9.88.

#### Synthesis of [Ru(tpy)Cl_2_]_2_ (4)

[(*p*-cymene)RuCl_2_]_2_ (200 mg, 0.33 mmol, 1 equiv) was dissolved in 4 mL of oxygen-free DCM under an N_2_ atmosphere followed by the addition of 2,6-di(2-pyridyl)pyridine (153 mg, 0.66 mmol, 2 equiv) in 16 mL of oxygen-free DCM over 1 h at r.t. After 30 min of stirring, the obtained dark purple precipitate was filtered and washed with DCM (3 × 6 mL), acetone (6 mL), and Et_2_O (6 mL). The solid was then transferred to a flask and dried *in vacuo* to afford compound **4** as a violet powder (208 mg, 0.26 mmol, 79%). ^1^H NMR (500 MHz, DMSO-*d*_6_) δ 9.36 (ddd, *J* = 5.6, 1.5, 0.7 Hz, 4H), 8.67 (d, *J* = 8.1 Hz, 4H), 8.59 (ddd, *J* = 8.1, 1.5, 0.7 Hz, 4H), 8.19 (t, *J* = 8.0 Hz, 2H), 7.99 (td, *J* = 7.8, 1.5 Hz, 4H), 7.53 (ddd, *J* = 7.3, 5.6, 1.4 Hz, 4H). ^13^C NMR (126 MHz, DMSO-*d*_6_) δ 158.79, 156.99, 155.76, 136.88, 136.19, 126.42, 123.11, 121.45.

#### Synthesis of [Ru(tpy)(biq)Cl]Cl (5)

Compound **4** (150 mg, 0.19 mmol, 1 equiv) was mixed with 2,2′-biquinoline (95 mg, 0.37 mmol, 2 equiv) and 2.3 mL of ethylene glycol under an N_2_ atmosphere followed by stirring at 180 °C over 1 h. The resulting mixture was then concentrated *in vacuo*, and the residue was recrystallized from MeOH/Et_2_O to afford compound **5** as a violet powder (282 mg, 0.34 mmol, 80% pure, 92%). ^1^H NMR (500 MHz, CD_3_OD) δ 9.41–9.31 (m, 1H), 8.69 (d, *J* = 8.9 Hz, 1H), 8.62 (dd, *J* = 8.9, 0.8 Hz, 1H), 8.38 (d, *J* = 8.8 Hz, 1H), 8.34 (d, *J* = 8.1 Hz, 2H), 8.18 (ddd, *J* = 8.1, 1.4, 0.8 Hz, 2H), 8.01 (dd, *J* = 8.0, 1.7 Hz, 1H), 7.97–7.93 (m, 1H), 7.88 (t, *J* = 8.1 Hz, 1H), 7.65–7.60 (m, 2H), 7.59 (ddd, *J* = 8.1, 6.8, 1.3 Hz, 1H), 7.57–7.52 (m, 3H), 7.50 (dd, *J* = 8.0, 1.5 Hz, 1H), 7.15 (ddd, *J* = 8.0, 6.9, 1.1 Hz, 1H), 7.02 (ddd, *J* = 7.6, 5.6, 1.3 Hz, 2H), 6.91 (ddd, *J* = 8.7, 6.9, 1.5 Hz, 1H), 6.50 (dq, *J* = 8.8, 0.8 Hz, 1H). ^13^C NMR (126 MHz, CD_3_OD) δ 163.18, 160.68, 160.34, 159.96, 153.86, 153.16, 152.49, 139.75, 138.84, 137.72, 136.82, 132.06, 131.83, 131.78, 130.75, 130.44, 129.96, 129.73, 129.64, 128.36, 124.93, 124.85, 123.95, 121.78, 121.71. ESI-MS: exact *m*/*z* calculated for [C_33_H_23_Cl_2_N_5_Ru–Cl]^+^: 626.1; found: 626.1.

#### Synthesis of [Ru(tpy)(biq)(STF31)]Cl_2_

The synthesis was done in the dark. Compound **5** (500 mg, 0.61 mmol, 1 eq, 80% pure), **STF31** (269 mg, 0.64 mmol, 1.05 equiv), and AgPF_6_ (320 mg, 1.24 mmol, 2.05 equiv) were mixed with 16 mL of oxygen-free acetone/H_2_O = 1/1. The resulting suspension was stirred at 50 °C overnight. The obtained pink-purple mixture was filtered, and the precipitate was washed with acetone. The filtrate was then concentrated *in vacuo* and redissolved in a minimal quantity of acetone followed by a slow addition of saturated acetone solution of TBAC (1.732 g, 6.05 mmol, 10 equiv). The resulting precipitate was filtered, washed with Et_2_O, and recrystallized from the MeOH/Et_2_O mixture. Purification by SEC in MeOH afforded the target compound as a pink-purple powder (380 mg, 0.35 mmol, 58%). Purity was >95%. ^1^H NMR (400 MHz, CD_3_OD) δ 9.19 (d, *J* = 8.9 Hz, 1H), 9.08 (d, *J* = 8.8 Hz, 1H), 8.99–8.89 (m, 2H), 8.82 (d, *J* = 2.4 Hz, 2H), 8.70 (d, *J* = 8.2 Hz, 1H), 8.52–8.45 (m, 2H), 8.41–8.32 (m, 2H), 8.21–8.11 (m, 2H), 8.02 (t, *J* = 7.9 Hz, 1H), 7.97 (d, *J* = 5.6 Hz, 1H), 7.89 (d, *J* = 6.7 Hz, 1H), 7.78–7.71 (m, 4H), 7.70–7.65 (m, 2H), 7.57–7.42 (m, 6H), 7.39–7.29 (m, 5H), 6.99 (dd, *J* = 8.4, 5.7 Hz, 1H), 6.82 (d, *J* = 8.8 Hz, 1H), 4.12 (s, 2H), 1.33 (s, 9H). ^13^C NMR (126 MHz, CD_3_OD) δ 168.03, 161.79, 160.68, 160.09, 159.98, 159.62, 157.47, 155.04, 153.74, 152.20, 151.25, 148.26, 143.95, 143.15, 141.04, 140.27, 140.02, 139.37, 139.08, 138.34, 133.81, 132.66, 132.44, 131.74, 131.38, 130.79, 130.36, 130.03, 129.85, 129.78, 128.96, 128.80, 127.89, 127.56, 127.18, 126.95, 126.84, 126.04, 125.75, 125.21, 124.68, 122.51, 122.27, 47.30, 35.99, 31.49. ESI-MS: exact *m*/*z* calculated for [C_56_H_48_Cl_2_N_8_O_3_RuS–Cl]^+^: 1049.2; found: 1049.1; exact *m*/*z* calculated for [C_56_H_48_Cl_2_N_8_O_3_RuS–2Cl]^2+^: 507.1; found: 506.8. Elemental analysis calculated for C_56_H_48_Cl_2_N_8_O_3_RuS (%): C, 61.99; H, 4.46; N, 10.33; found: C, 60.74; H, 4.36; N, 10.14.

#### Synthesis of [Ru(tpy)(biq)(Py)]Cl_2_

The synthesis was done in the dark. Compound **5** (615 mg, 0.74 mmol, 1 eq, 80% pure) and pyridine (1.21 mL, *d* = 0.98 g/mL, 14.87 mmol, 20 equiv) were mixed with 4 mL of oxygen-free ethanol/H_2_O = 1/1, and the resulting violet solution was stirred at 100 °C overnight. The obtained pink-purple mixture was then concentrated *in vacuo*, and the residue was recrystallized from the MeOH/Et_2_O mixture. Purification by SEC in MeOH afforded the target compound as a pink-purple powder (490 mg, 0.66 mmol, 89%). Purity was >95%. ^1^H NMR (500 MHz, CD_3_OD) δ 9.19 (d, *J* = 8.9 Hz, 1H), 9.08 (d, *J* = 8.8 Hz, 1H), 8.92 (d, *J* = 8.8 Hz, 1H), 8.81 (d, *J* = 8.1 Hz, 2H), 8.65–8.59 (m, 2H), 8.50–8.43 (m, 1H), 8.41–8.33 (m, 2H), 8.12–8.04 (m, 4H), 7.91–7.86 (m, 1H), 7.79–7.70 (m, 4H), 7.53–7.45 (m, 3H), 7.34–7.28 (m, 3H), 7.13–7.05 (m, 2H), 6.77 (dq, *J* = 8.9, 0.9 Hz, 1H). ^13^C NMR (126 MHz, CD_3_OD) δ 161.78, 160.72, 159.75, 159.73, 154.43, 152.69, 152.15, 151.12, 141.10, 140.27, 140.06, 139.96, 138.39, 132.66, 132.49, 131.75, 131.42, 130.79, 130.38, 130.03, 129.99, 129.86, 127.47, 127.45, 126.23, 125.53, 124.59, 122.58, 122.27. ESI-MS: exact *m*/*z* calculated for [C_38_H_28_Cl_2_N_6_Ru–2Cl]^2+^: 335.1; found: 334.9; exact *m*/*z* calculated for [C_38_H_28_Cl_2_N_6_Ru–2Cl–Py+MeOH]^2+^: 311.6; found: 311.4; exact *m*/*z* calculated for [C_38_H_28_Cl_2_N_6_Ru–2Cl–Py]^2+^: 295.5; found: 295.4. Elemental analysis calculated for C_38_H_28_Cl_2_N_6_Ru (%): C, 61.62; H, 3.81; N, 11.35; found: C, 61.15; H, 3.91; N, 11.25.

### Expression Data Retrieval from Human Protein Atlas

NAMPT expression in glioma patients and brain cancer cell lines assembled from the Human Protein Atlas (version 23.0), Ensemble (version 109).

### Whole Transcriptome Analysis (TempO-Seq)

Targeted whole transcriptome analysis was performed using TempO-Seq (Yeakley *et al.*, 2017). Three biological replications were made for each hypoxic and normoxic cell sample. Parallel samples were included for the Western blotting of the NAMPT protein. All cell lines were grown for 14 days in hypoxic (<1% O_2_) and normoxic (21% O_2_) conditions in three different T75 cell culture flasks (Thermo Fisher Scientific, cat. No: 156472) in complete DMEM . On day 14, cells were then seeded in a 96-well plate (Thermo Fisher Scientific 10 334 513) with different densities of 50 000, 80 000, 40 000, 60 000, 1 00 000 cells/mL for A549, A431, A375, U87MG, and HepG2 (from ATCC) subsequently. Cells were then grown for 5 days under normoxia or hypoxia, and on day 19, the cells were lysed by adding 1X TempO-seq lysis buffer (20 μL from BioSpyder Technologies Inc., Carlsbad, CA, USA) in each well. The cell lysate was stored at −80 °C and sent to the BioSpyder company for TempO-Seq analysis. An R script developed in-house was used for count normalization and differential gene expression analysis. The minimum library size (total number of reads per sample) was set as 1 00 000 reads, and samples below this size were removed. The CPM package^58^ was used for count data normalization and to generate adjusted *p*-value (padj) and log2FoldChange values. Differentially expressed genes (DEGs) were selected by |log2FoldChange| > 1, and padj < 0.01 was used for making graphs using the GraphPad Prism 9.0.0 software.

### Cell Treatment and SRB Cell Viability Assay

At *t* = 0 h, U87MG and HepG2 cells in complete DMEM were seeded into the 60 central wells of 96-well plates (Thermo Fisher Scientific 10 334 513) with a density of 60 000 cells/mL and 10 000 cells/mL, respectively, using a seeding volume of 100 μL. The outer wells were filled with 100 μL PBS to minimize border effects. At *t* = 24 h, the medium was refreshed, and the cells were treated with six different compound concentrations. Cells at *t* = 48 h were irradiated at 630 nm for 42 min in normoxia (8.7 mW/cm^2^) and 51 min in hypoxia (6.8 mW/cm^2^) to achieve a light dose of 21 J/cm^2^, and plates without being treated by light were kept in the dark. At the end-point (*t* = 96 h), cells were fixed by adding cold trichloroacetic acid (10% w/v 100 μL in each well) for performing the SRB assay.^59^ The plates were stored at 4 °C for 2 days, then the TCA medium mixture was removed, and the cells were rinsed with demineralized water three times and dried overnight. Then, each well was stained with 100 μL S34 SRB solution (0.6% w/v SRB in 1% v/v acetic acid, Sigma-Aldrich, CAS number: 3520–42–1) for 30 min, and the SRB solution was removed and washed with acetic acid (1% v/v) for 3–5 times. Once the plates were dried overnight, 200 μL of tris base (tromethamine, 10 mM, Sigma-Aldrich, USA) was pipetted to each well. To determine the cell viability, the absorbance at 510 nm was measured using a M1000 Tecan Reader. The SRB absorbance data per compound per concentration were interred to GraphPad Prism. The EC_50_ value was calculated after the background (absorbance of wells filled with PBS during treatment), and concentrations (C in μM) were transformed to X=log(C). The two-parametric Hill slope equation with a fixed maximum (100%) and minimum (0%) relative cell population values (*Y*) were used to calculate EC_50_ values.^60^
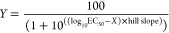
3

### Combination Therapy

The Chou-Talalay method is a mathematical tool used to investigate the nature of drug interactions in combination therapy.^61^ This method introduces the combination index value (CI) for quantifying synergism or antagonism for two drugs, calculated according to eq 2. In combination treatment, **Ru-Py** (activated by RL, 630 nm, 21 J/cm^2^) and **STF31**, normoxic, and hypoxic U87MG cells were seeded in 96 wells with a density of 80 000 cells/mL. At *t* = 24 h, cells in columns C-D-E were treated with **STF31** alone in 6 different concentrations (100–1.06 μM), cells in columns F-G-H-I-J-K were treated with **Ru-Py** alone in 6 different concentrations (100–1.06 μM), and the cells were incubated further in the dark. At *t* = 48 h, cells were irradiated with red light (630 nm, 21 J/cm^2^), corresponding to 42 min in normoxia (8.7 mW/cm^2^) and 51 min in hypoxia (6.8 mW/cm^2^). Immediately after irradiation, all cells were washed twice with a drug-free medium, and **STF31** was added to the cells in columns I-J-K pretreated by **Ru-Py** (RL), with a 1:1 concentration ratio (100–1.06 μM). The plates were incubated further in the dark. At *t* = 96 h, cells were fixed and an SRB assay for evaluating cell viability was performed. The SRB absorbance in each condition was averaged over three identical technical replicates (*n* = 3) using Excel and imported into CompuSyn software, which generated the CompuSyn report including the combination index graphs shown in Figure 6.

For the NAD^+^ combination treatment, all compounds were initially dissolved in DMSO, followed by dilution with PBS to a concentration of 1 mM of the compound with a maximum of 0.5% DMSO. The solution was further diluted with DMEM (Dulbecco’s Modified Eagle Medium) to achieve a 300 μM concentration of the compound. Cells were seeded at *t* = 48 h after cell seeding, and the 96-well plates were irradiated with red light with a wavelength of 630 nm and light dose of 21 J/cm^2^. The irradiated plates were maintained in a plate holder set at 37 °C, with irradiation lasting 51 min in hypoxia and 42 min in normoxia. Immediately post-irradiation, cells were treated with NAD^+^, which was dissolved in PBS to 2 mM and further diluted to working concentrations with the medium. At *t* = 96 h, *i.e.*, 48 h after light activation, cells were fixed and SRB cell viability assay was used to quantify the EC_50_ values.

### Western Blotting

All antibodies were provided by Cell Signaling, The Netherlands. 20 μg protein from the cell lines A375 (skin cancer), A549 (lung cancer), A431 (skin cancer), HepG2 (liver cancer), and U87MG (glioblastoma) cultured in normoxic and hypoxic conditions in complete DMEM for 19 days were loaded on 4–15% polyacrylamide, mini-protein precast gels (Bio-Rad, The Netherlands). The cells were provided by the American Type Culture Collection (ATCC) company. The running buffer consisted of 100 mL of 10X tris-glycine buffer, 10 mL of 10% SDS, 700 mL of MiliQ water, and 200 mL of methanol (MeOH). The gels were run for 5 min at 200 V and 1 h at 120 V. Subsequently, the proteins on the gels were transferred to the PVDF (polyvinylidene difluoride) membrane by Trans-Blot Turbo transfer system required by Bio-Rad company (#1704150), The Netherlands. After the transfer of the proteins, the membranes were blocked with 5 mL of 5% milk in 0.1% TBS-Tween for 1 h at room temperature. Then, the membranes were incubated overnight with NAMPT antibody (3 mL of 5% milk in 0.1% TBS-T (tris-buffered saline and Tween 20) dilution: 1:1000, rabbit, mAb #86634) at 4 °C on the IKEA roller shaker. The membranes were washed 5 times for 3 min in 0.1% TBS-T and incubated with the secondary antibody antirabbit, #7074 (3 mL, 0.1% TBS-T, dilution is 1:1000) for 1 h at 24 °C. After incubation with the secondary antibody, the membranes were washed 5 times for 3 min with 0.1% TBS-T and were imaged by colorimetry and chemiluminescence with a Bio-Rad ChemiDoc Imaging System (#12003263). α-tubulin, 50 kDa, was used as the housekeeping gene. For blotting the housekeeping gene protein, the membranes were incubated with the α-tubulin primary antibody (mouse, 1:1000 in 3 mL of 5% milk in 0.1% TBS-T) overnight at 4 °C on the roller bank. After incubation, the membranes were washed 5 times with 0.1% TBS-T and were incubated with the secondary antibody in 5% milk in 0.1% TBS-T (antirabbit, 1:1000) for 1 h at room temperature. The membranes were washed three times for 3 min in 0.1% TBS-T and were imaged by colorimetry and chemiluminescence with a Bio-Rad Imager.

### Metabolomic Study

U87MG cells cultured in complete DMEM for 14 days in hypoxic or normoxic conditions were seeded in 6-well plates, with a density of 1 50 000 cells/well. In normoxic conditions, cells were treated with a 15 μM concentration for all compounds, which was intermediate between the EC_50,RL_ value of **Ru-Py** (32.9 μM) and that of **Ru-STF31** (9.7 μM). As in hypoxic cells we had observed resistance to our treatment and the EC_50,RL_ values were almost two times higher than that in normoxic cells (22.7 and 9.72 μM for hypoxic and normoxic U87MG, respectively), we treated cells with 30 μM of all compounds in such conditions. 72 h after light irradiation (red light wavelength of 630 nm and light dose of 21 J/cm^2^ for 42 min in normoxia and 51 min in hypoxia), the cells were lyzed and collected to probe the changes in NAD^+^ level using published metabolomics methods. For cell quenching and harvest, the medium was removed from all wells, and cells were washed with PBS and immediately quenched with cold 80% methanol. The content of each well was transferred into an Eppendorf tube and put into liquid nitrogen for fast freezing. All samples were later transferred to a −80 °C freezer for long-term storage before analysis.

For sample preparation, cell samples were lysed with sonication after one freeze-thaw cycle, vortexed, and then centrifuged at 16 000g at 4 °C for 10 min. Cell pellets were collected to measure the protein content using a bicinchoninic acid assay (Cell Signaling, #7780). Supernatants were transferred into clean 1.5 mL Eppendorf tubes and evaporated to dryness in a Labconco SpeedVac (MO, USA). Each sample was reconstituted with 60 μL of ice-cold methanol/water (80%/20%; v/v). 50 μL of the reconstitution volume was collected and transferred into a new Eppendorf tube. The leftover volume was pooled together and aliquoted as quality control (QC) samples. 50 μL of cellular sample and QC samples were spiked with 5 μL of internal standard solution. A double liquid–liquid extraction (LLE) method was applied to treat samples by using mixed solvent chloroform/methanol/water (1/1/1, v/v/v). The upper aqueous phase was collected and evaporated to dryness.^45^ The residue was reconstituted with 50 μL of ice-cold methanol/water (1/1, v/v). Three μL of the final sample solution was used for LC-MS analysis. Metabolites including organic acids, amino acids, sugar phosphates, and nucleotides were measured on a HILIC-MS platform, which consisted of a SHIMAZU LC-30AD system with a SeQuant ZIC-cHILIC HPLC Analytical PEEK Column (Merck) coupled to electrospray ionization on a triple time-of-flight mass spectrometer (AB SCIEX TripleTOF 5600). The mobile phases were composed of (A) 90% acetonitrile in H_2_O with 5 mM ammonium formate and (B) 10% acetonitrile in H_2_O with 5 mM ammonium formate. Chromatographic separation of analytes was carried out with a gradient elution program at a flow rate of 0.5 mL/min.^44,45^ Electrospray ionization MS was operated in the negative ion mode, and analytes were monitored in the time-of-flight (ToF) mode at a full scan range of 50–900 *m*/*z*.^44,45^ MultiQuant software (AB SCIEX, version 3.0.1) was used in the quantitative analysis for LC-MS raw data extraction and peak area integration. Internal standards were employed to correct random errors during sample preparation. Pooled QC samples were used to compensate for shifts in the sensitivity of the mass spectrometer over the batches based on the in-house developed algorithms, mzQuality.^39^ Corrected metabolite abundance was further normalized to the amount of protein in each sample. The GraphPad Prism software, version 9.0.0, was used for further analysis and for making graphs.

## Supporting Information Available

Supporting Information including characterization data: NMR, ESI-MS, single crystal X-ray crystallography for **Ru-STF31**, **Ru-Py**, and **STF31**, and CompuSyn report for **Ru-Py** and **STF31** (PDF)
Supporting Information including molecular formula strings (CSV)

## Abbreviations

5-ALA: 5-aminolevulinic acid
ACN: acetonitrile
ALDOA: aldolase, fructose-bisphosphate A
ANOVA: analysis of variance
ATCC: American Type Culture Collection
CA9: carbonic anhydrase 9
CI: combination index
CNS: Global Cancer Statistics
DMEM: Dulbecco’s Modified Eagle Medium
EC_50_: the concentration of a drug that gives a half-maximal response
ENO1 and 2: enolase 1 and 2
ESI-MS: electrospray ionization mass spectrometry
Et_2_O: diethyl ether
FCS: fetal calf serum
FGR: fluorescence-guided resection
FPKM: fragments per kilobase of exon per million mapped fragments
GAPDH: glyceraldehyde-3-phosphate dehydrogenase
GLUT1: glucose transporter 1
HBF4: tetrafluoroboric acid
HIF1-α: hypoxia-inducible factor 1-alpha
HPLC: high-performance liquid chromatography
*In vivo*: in the body
*In vitro*: outside the body or in the laboratory
MCTS: multicellular tumor spheroids
LC-MS,: liquid chromatography–mass spectrometry
NAPRT: nicotinate phosphoribosyltransferase
PACT: photoactivated chemotherapy
PDT: photodynamic therapy
PI: photo index
PVDF: polyvinylidene difluoride
QC: quality control
RNA-seq: RNA sequencing
ROS: reactive oxygen species
SDS: sodium dodecyl sulfate
SLC: solute carrier
SLC29A2,: solute carrier family 29 member 2
SRB: sulforhodamine B
TCGA: The Cancer Genome Atlas Program
TBS-T: tris-buffered saline and Tween 20

## References

[ref1] Ostrom Q. T.; Cioffi G.; Gittleman H.; Patil N.; Waite K.; Kruchko C.; Barnholtz-Sloan J. S. CBTRUS Statistical Report: Primary Brain and Other Central Nervous System Tumors Diagnosed in the United States in 2012–2016. Neuro. Oncol. 2019, 21 (Suppl 5), v1–v100. 10.1093/neuonc/noz150. 31675094 PMC6823730

[ref2] Chinot O. L.; Wick W.; Mason W.; Henriksson R.; Saran F.; Nishikawa R.; Carpentier A. F.; Hoang-Xuan K.; Kavan P.; Cernea D.; Brandes A. A.; Hilton M.; Abrey L.; Cloughesy T. Bevacizumab plus Radiotherapy–Temozolomide for Newly Diagnosed Glioblastoma. N. Engl. J. Med. 2014, 370 (8), 709–722. 10.1056/NEJMoa1308345. 24552318

[ref3] Gilbert M. R.; Wang M.; Aldape K. D.; Stupp R.; Hegi M. E.; Jaeckle K. A.; Armstrong T. S.; Wefel J. S.; Won M.; Blumenthal D. T.; Mahajan A.; Schultz C. J.; Erridge S.; Baumert B.; Hopkins K. I.; Tzuk-Shina T.; Brown P. D.; Chakravarti A.; Curran W. J.; Mehta M. P. Dose-Dense Temozolomide for Newly Diagnosed Glioblastoma: A Randomized Phase III Clinical Trial. J. Clin. Oncol. 2013, 31 (32), 4085–4091. 10.1200/JCO.2013.49.6968. 24101040 PMC3816958

[ref4] Gramatzki D.; Dehler S.; Rushing E. J.; Zaugg K.; Hofer S.; Yonekawa Y.; Bertalanffy H.; Valavanis A.; Korol D.; Rohrmann S.; Pless M.; Oberle J.; Roth P.; Ohgaki H.; Weller M. Glioblastoma in the Canton of Zurich, Switzerland Revisited: 2005 to 2009. Cancer 2016, 122 (14), 2206–2215. 10.1002/cncr.30023. 27088883

[ref5] Fernandez-Palomo C.; Chang S.; Prezado Y. Should Peak Dose Be Used to Prescribe Spatially Fractionated Radiation Therapy?—A Review of Preclinical Studies. Cancers 2022, 14 (15), 3625 10.3390/cancers14153625. 35892895 PMC9330631

[ref6] Park J. H.; Lee H. K. Current Understanding of Hypoxia in Glioblastoma Multiforme and Its Response to Immunotherapy. Cancers 2022, 14 (5), 1176 10.3390/cancers14051176. 35267480 PMC8909860

[ref7] Cramer S. W.; Chen C. C. Photodynamic Therapy for the Treatment of Glioblastoma. Front. Surg 2020, 6, 81 10.3389/fsurg.2019.00081. 32039232 PMC6985206

[ref8] Bhowmik A.; Khan R.; Ghosh M. K. Blood Brain Barrier: A Challenge for Effectual Therapy of Brain Tumors. BioMed. Res. Int. 2015, 2015, 1–20. 10.1155/2015/320941. PMC4383356 25866775

[ref9] Aldape K.; Brindle K. M.; Chesler L.; Chopra R.; Gajjar A.; Gilbert M. R.; Gottardo N.; Gutmann D. H.; Hargrave D.; Holland E. C.; Jones D. T. W.; Joyce J. A.; Kearns P.; Kieran M. W.; Mellinghoff I. K.; Merchant M.; Pfister S. M.; Pollard S. M.; Ramaswamy V.; Rich J. N.; Robinson G. W.; Rowitch D. H.; Sampson J. H.; Taylor M. D.; Workman P.; Gilbertson R. J. Challenges to Curing Primary Brain Tumours. Nat. Rev. Clin. Oncol 2019, 16 (8), 509–520. 10.1038/s41571-019-0177-5. 30733593 PMC6650350

[ref10] Dupont C.; Mordon S. R.; Deleporte P.; Reyns N.; Vermandel M. A Novel Device for Intraoperative Photodynamic Therapy Dedicated to Glioblastoma Treatment. Future Oncol. 2017, 13, 2441–2454. 10.2217/fon-2017-0261. 28942677

[ref11] Monro S.; Colón K. L.; Yin H.; Roque J. I.; Konda P.; Gujar S.; Thummel R. P.; Lilge L.; Cameron C. G.; McFarland S. A. Transition Metal Complexes and Photodynamic Therapy from a Tumor-Centered Approach: Challenges, Opportunities, and Highlights from the Development of TLD1433. Chem. Rev. 2019, 119 (2), 797–828. 10.1021/acs.chemrev.8b00211. 30295467 PMC6453754

[ref12] Foglar M.; Aumiller M.; Bochmann K.; Buchner A.; El Fahim M.; Quach S.; Sroka R.; Stepp H.; Thon N.; Forbrig R.; Rühm A. Interstitial Photodynamic Therapy of Glioblastomas: A Long-Term Follow-up Analysis of Survival and Volumetric MRI Data. Cancers 2023, 15 (9), 2603 10.3390/cancers15092603. 37174068 PMC10177153

[ref13] Huang Z.; Hsu Y.-C.; Li L.-B.; Wang L.-W.; Song X.-D.; Yow C. M. N.; Lei X.; Musani A. I.; Luo R.-C.; Day B. J. Photodynamic Therapy of Cancer — Challenges of Multidrug Resistance. J. Innov. Opt. Health Sci. 2015, 08 (1), 1530002 10.1142/S1793545815300025.

[ref14] Broekgaarden M.; Weijer R.; van Gulik T. M.; Hamblin M. R.; Heger M. Tumor Cell Survival Pathways Activated by Photodynamic Therapy: A Molecular Basis for Pharmacological Inhibition Strategies. Cancer Metastasis Rev. 2015, 34 (4), 643–690. 10.1007/s10555-015-9588-7. 26516076 PMC4661210

[ref15] Shahmoradi Ghahe S.; Kosicki K.; Wojewódzka M.; Majchrzak B. A.; Fogtman A.; Iwanicka-Nowicka R.; Ciuba A.; Koblowska M.; Kruszewski M.; Tudek B.; Speina E. Increased DNA Repair Capacity Augments Resistance of Glioblastoma Cells to Photodynamic Therapy. DNA Repair 2021, 104, 103136 10.1016/j.dnarep.2021.103136. 34044336

[ref16] Chen Y.; Bai L.; Zhang P.; Zhao H.; Zhou Q. The Development of Ru(II)-Based Photoactivated Chemotherapy Agents. Molecules 2021, 26, 5679 10.3390/molecules26185679. 34577150 PMC8465985

[ref17] Havrylyuk D.; Hachey A.; Glazer E. Light-Activated Drugs for Photodynamic and Photoactivated Therapy. In Targeted Metallo-Drugs; CRC Press, 2023; pp. 39 65.

[ref18] Truong V. X.; Barner-Kowollik C. Photodynamic Covalent Bonds Regulated by Visible Light for Soft Matter Materials. Trends Chem. 2022, 4 (4), 291–304. 10.1016/j.trechm.2022.01.011.

[ref19] Cordones A. A.; Lee J. H.; Hong K.; Cho H.; Garg K.; Boggio-Pasqua M.; Rack J. J.; Huse N.; Schoenlein R. W.; Kim T. K. Transient Metal-Centered States Mediate Isomerization of a Photochromic Ruthenium-Sulfoxide Complex. Nat. Commun. 2018, 9 (1), 1989 10.1038/s41467-018-04351-0. 29777157 PMC5959936

[ref20] Lameijer L. N.; Ernst D.; Hopkins S. L.; Meijer M. S.; Askes S. H. C.; Le Dévédec S. E.; Bonnet S. A Red-Light-Activated Ruthenium-Caged NAMPT Inhibitor Remains Phototoxic in Hypoxic Cancer Cells. Angew. Chem., Int. Ed. Engl. 2017, 56 (38), 11549–11553. 10.1002/anie.201703890. 28666065 PMC5601216

[ref21] van Rixel V. H. S.; Ramu V.; Auyeung A. B.; Beztsinna N.; Leger D. Y.; Lameijer L. N.; Hilt S. T.; Le Dévédec S. E.; Yildiz T.; Betancourt T.; Gildner M. B.; Hudnall T. W.; Sol V.; Liagre B.; Kornienko A.; Bonnet S. Photo-Uncaging of a Microtubule-Targeted Rigidin Analogue in Hypoxic Cancer Cells and in a Xenograft Mouse Model. J. Am. Chem. Soc. 2019, 141 (46), 18444–18454. 10.1021/jacs.9b07225. 31625740 PMC11774275

[ref22] Potential of organometallic complexes in medicinal chemistry - ScienceDirect. https://www.sciencedirect.com/science/article/pii/S1367593112000051. accessed 11 March 2024.

[ref23] Kumar P.; Mondal I.; Kulshreshtha R.; Patra A. K. Development of Novel Ruthenium(II)–Arene Complexes Displaying Potent Anticancer Effects in Glioblastoma Cells. Dalton Trans. 2020, 49 (38), 13294–13310. 10.1039/D0DT02167A. 32936191

[ref24] Bretin L.; Husiev Y.; Ramu V.; Zhang L.; Hakkennes M.; Abyar S.; Johns A. C.; Le Dévédec S. E.; Betancourt T.; Kornienko A.; Bonnet S. Red-Light Activation of a Microtubule Polymerization Inhibitor via Amide Functionalization of the Ruthenium Photocage. Angew. Chem. 2024, 63, e202316425 10.1002/anie.202316425. 38061013

[ref25] Zhang L.; Wang P.; Zhou X.-Q.; Bretin L.; Zeng X.; Husiev Y.; Polanco E. A.; Zhao G.; Wijaya L. S.; Biver T.; Le Dévédec S. E.; Sun W.; Bonnet S. Cyclic Ruthenium-Peptide Conjugates as Integrin-Targeting Phototherapeutic Prodrugs for the Treatment of Brain Tumors. J. Am. Chem. Soc. 2023, 145 (27), 14963–14980. 10.1021/jacs.3c04855. 37379365 PMC10347550

[ref26] Tan A.; Doig C. L. NAD+ Degrading Enzymes, Evidence for Roles During Infection. Front. Mol. Biosci. 2021, 8, 697359 10.3389/fmolb.2021.697359. 34485381 PMC8415550

[ref27] Amjad S.; Nisar S.; Bhat A. A.; Shah A. R.; Frenneaux M. P.; Fakhro K.; Haris M.; Reddy R.; Patay Z.; Baur J.; Bagga P. Role of NAD+ in Regulating Cellular and Metabolic Signaling Pathways. Mol. Metab. 2021, 49, 101195 10.1016/j.molmet.2021.101195. 33609766 PMC7973386

[ref28] Elhassan Y. S.; Philp A. A.; Lavery G. G. Targeting NAD+ in Metabolic Disease: New Insights Into an Old Molecule. J. Endocr Soc. 2017, 1 (7), 816–835. 10.1210/js.2017-00092. 29264533 PMC5686634

[ref29] Thongon N.; Zucal C.; D’Agostino V. G.; Tebaldi T.; Ravera S.; Zamporlini F.; Piacente F.; Moschoi R.; Raffaelli N.; Quattrone A.; Nencioni A.; Peyron J.-F.; Provenzani A. Cancer Cell Metabolic Plasticity Allows Resistance to NAMPT Inhibition but Invariably Induces Dependence on LDHA. Cancer Metab. 2018, 6 (1), 1 10.1186/s40170-018-0174-7. 29541451 PMC5844108

[ref30] Bordone L.; Motta M. C.; Picard F.; Robinson A.; Jhala U. S.; Apfeld J.; McDonagh T.; Lemieux M.; McBurney M.; Szilvasi A.; Easlon E. J.; Lin S.-J.; Guarente L. Sirt1 Regulates Insulin Secretion by Repressing UCP2 in Pancreatic β Cells. PloS Biol. 2005, 4 (2), e31 10.1371/journal.pbio.0040031. 16366736 PMC1318478

[ref31] van der Horst A.; Tertoolen L. G. J.; de Vries-Smits L. M. M.; Frye R. A.; Medema R. H.; Burgering B. M. T. FOXO4 Is Acetylated upon Peroxide Stress and Deacetylated by the Longevity Protein hSir2(SIRT1). J. Biol. Chem. 2004, 279 (28), 28873–28879. 10.1074/jbc.M401138200. 15126506

[ref32] Jieyu H.; Chao T.; Mengjun L.; Shalong W.; Xiaomei G.; Jianfeng L.; Zhihong L. Nampt/Visfatin/PBEF: A Functionally Multi-Faceted Protein with a Pivotal Role in Malignant Tumors. Curr. Pharm. Des. 2012, 18 (37), 6123–6132. 10.2174/138161212803582531. 22934941

[ref33] Zhao G.; Green C.; Hui Y.-H.; Prieto L.; Shepard R.; Dong S.; Wang T.; Tan B.; Gong X.; Kays L.; Johnson R.; Wu W.; Bhattachar S.; Prado M.; Gillig J.; Fernandez M.-C.; Roth K.; Buchanan S.; Kuo M.-S.; Burkholder T. Discovery of a Highly Selective NAMPT Inhibitor That Demonstrates Robust Efficacy and Improved Retinal Toxicity with Nicotinic Acid Co-Administration. Mol. Cancer Ther. 2017, 16, 2677–2688. 10.1158/1535-7163.MCT-16-0674. 29054982

[ref34] Adams D. J.; Ito D.; Rees M. G.; Seashore-Ludlow B.; Puyang X.; Ramos A. H.; Cheah J. H.; Clemons P. A.; Warmuth M.; Zhu P.; Shamji A. F.; Schreiber S. L. NAMPT Is the Cellular Target of STF-31-like Small-Molecule Probes. ACS Chem. Biol. 2014, 9 (10), 2247–2254. 10.1021/cb500347p. 25058389 PMC4201331

[ref35] Cuello-Garibo J.-A.; Meijer M. S.; Bonnet S. To Cage or to Be Caged? The Cytotoxic Species in Ruthenium-Based Photoactivated Chemotherapy Is Not Always the Metal. Chem. Commun. 2017, 53 (50), 6768–6771. 10.1039/C7CC03469E. PMC5708332 28597879

[ref36] Azar D. F.; Audi H.; Farhat S.; El-Sibai M.; Abi-Habib R. J.; Khnayzer R. S. Phototoxicity of Strained Ru(II) Complexes. Dalton Trans. 2017, 46 (35), 11529–11532. 10.1039/C7DT02255G. 28748239

[ref37] Saha A.; Mondal I.; Kumari A.; Sonkar A. K.; Mishra R.; Kulshreshtha R.; Patra A. K. Hyphenation of Lipophilic Ruthenium(Ii)-Diphosphine Core with 5-Fluorouracil: An Effective Metallodrug against Glioblastoma Brain Cancer Cells. Dalton Trans. 2024, 53 (4), 1551–1567. 10.1039/D3DT02941G. 38164612

[ref38] Fulmer G. R.; Miller A. J. M.; Sherden N. H.; Gottlieb H. E.; Nudelman A.; Stoltz B. M.; Bercaw J. E.; Goldberg K. I. NMR Chemical Shifts of Trace Impurities: Common Laboratory Solvents, Organics, and Gases in Deuterated Solvents Relevant to the Organometallic Chemist. Organometallics 2010, 29 (9), 2176–2179. 10.1021/om100106e.

[ref39] Lameijer L. N.; Ernst D.; Hopkins S. L.; Meijer M. S.; Askes S. H. C.; Bonnet S. A Red-Light-Activated Ruthenium-Caged NAMPT Inhibitor Remains Phototoxic in Hypoxic Cancer Cells. Angew. Chem., Int. Ed. 2017, 56 (38), 11549–11553. 10.1002/anie.201703890. PMC5601216 28666065

[ref40] Wei Y.; Xiang H.; Zhang W. Review of Various NAMPT Inhibitors for the Treatment of Cancer. Front. Pharmacol 2022, 13, 970553 10.3389/fphar.2022.970553. 36160449 PMC9490061

[ref200] Uhlen M.; Fagerberg L.; Hallstrom B. M.; Lindskog C.; Oksvold P.; Mardinoglu A.; Sivertsson A.; Kampf C.; Sjostedt E.; Asplund A.; Olsson I.; Edlund K.; Lundberg E.; Navani S.; Szigyarto C. A.-K.; Odeberg J.; Djureinovic D.; Takanen J. O.; Hober S.; Alm T.; Edqvist P.-H.; Berling H.; Tegel H.; Mulder J.; Rockberg J.; Nilsson P.; Schwenk J. M.; Hamsten M.; von Feilitzen K.; Forsberg M.; Persson L.; Johansson F.; Zwahlen M.; von Heijne G.; Nielsen J.; Ponten F. Proteomics. Tissue-based map of the human proteome. PubMed 2015, 347, 1260419 10.1126/science.1260419. 25613900

[ref41] Rana N. K.; Singh P.; Koch B. CoCl2 Simulated Hypoxia Induce Cell Proliferation and Alter the Expression Pattern of Hypoxia Associated Genes Involved in Angiogenesis and Apoptosis. Biol. Res. 2019, 52, 12 10.1186/s40659-019-0221-z. 30876462 PMC6419504

[ref42] Chou T.-C. Derivation and Properties of Michaelis-Menten Type and Hill Type Equations for Reference Ligands. J. Theor. Biol. 1976, 59 (2), 253–276. 10.1016/0022-5193(76)90169-7. 957690

[ref43] Chou T.-C. Theoretical Basis, Experimental Design, and Computerized Simulation of Synergism and Antagonism in Drug Combination Studies. Pharmacol. Rev. 2006, 58 (3), 621–681. 10.1124/pr.58.3.10. 16968952

[ref44] Huang L.; Drouin N.; Causon J.; Wegrzyn A.; Castro-Perez J.; Fleming R.; Harms A.; Hankemeier T. Reconstruction of Glutathione Metabolism in the Neuronal Model of Rotenone-Induced Neurodegeneration Using Mass Isotopologue Analysis with Hydrophilic Interaction Liquid Chromatography-Zeno High-Resolution Multiple Reaction Monitoring. Anal. Chem. 2023, 95 (6), 3255–3266. 10.1021/acs.analchem.2c04231. 36735349 PMC9933045

[ref45] Hosseinkhani F.; Huang L.; Dubbelman A.-C.; Guled F.; Harms A. C.; Hankemeier T. Systematic Evaluation of HILIC Stationary Phases for Global Metabolomics of Human Plasma. Metabolites 2022, 12 (2), 165 10.3390/metabo12020165. 35208239 PMC8875576

[ref46] Okabe K.; Yaku K.; Tobe K.; Nakagawa T. Implications of Altered NAD Metabolism in Metabolic Disorders. J. Biomed. Sci. 2019, 26 (1), 34 10.1186/s12929-019-0527-8. 31078136 PMC6511662

[ref47] Vachher M.; Arora K.; Burman A.; Kumar B. NAMPT, GRN, and SERPINE1 Signature as Predictor of Disease Progression and Survival in Gliomas. J. Cell. Biochem. 2020, 121 (4), 3010–3023. 10.1002/jcb.29560. 31710121

[ref48] Lucena-Cacace A.; Otero-Albiol D.; Jiménez-García M. P.; Peinado-Serrano J.; Carnero A. NAMPT Overexpression Induces Cancer Stemness and Defines a Novel Tumor Signature for Glioma Prognosis. Oncotarget 2017, 8 (59), 99514–99530. 10.18632/oncotarget.20577. 29245920 PMC5725111

[ref49] Lopez J. S.; Banerji U. Combine, and Conquer: Challenges for Targeted Therapy Combinations in Early Phase Trials. Nat. Rev. Clin. Oncol. 2017, 14 (1), 57–66. 10.1038/nrclinonc.2016.96. 27377132 PMC6135233

[ref50] Robey I. F.; Lien A. D.; Welsh S. J.; Baggett B. K.; Gillies R. J. Hypoxia-Inducible Factor-1α and the Glycolytic Phenotype in Tumors. Neoplasia 2005, 7 (4), 324–330. 10.1593/neo.04430. 15967109 PMC1501147

[ref51] Lewis J. E.; Singh N.; Holmila R. J.; Sumer B. D.; Williams N. S.; Furdui C. M.; Kemp M. L.; Boothman D. A. Targeting NAD+ Metabolism to Enhance Radiation Therapy Responses. Semin Radiat Oncol. 2019, 29 (1), 6–15. 10.1016/j.semradonc.2018.10.009. 30573185 PMC6310039

[ref52] Smith M. J.; Yang F.; Griffiths A.; Morrell A.; Chapple S. J.; Siow R. C. M.; Stewart T.; Maret W.; Mann G. E. Redox and Metal Profiles in Human Coronary Endothelial and Smooth Muscle Cells under Hyperoxia, Physiological Normoxia and Hypoxia: Effects of NRF2 Signaling on Intracellular Zinc. Redox Biol. 2023, 62, 102712 10.1016/j.redox.2023.102712. 37116256 PMC10165141

[ref53] Matsumoto T.; Jimi S.; Migita K.; Takamatsu Y.; Hara S. Inhibition of Glucose Transporter 1 Induces Apoptosis and Sensitizes Multiple Myeloma Cells to Conventional Chemotherapeutic Agents. Leuk. Res. 2016, 41, 103–110. 10.1016/j.leukres.2015.12.008. 26790725

[ref54] Ghanem M. S. 1.; Monacelli F. 2.; Nencioni A. Advances in NAD-Lowering Agents for Cancer Treatment. Nutrients 2021, 13 (5), 1665 10.3390/nu13051665. 34068917 PMC8156468

[ref55] Guda M. R.; Labak C. M.; Omar S. I.; Asuthkar S.; Airala S.; Tuszynski J.; Tsung A. J.; Velpula K. K. GLUT1 and TUBB4 in Glioblastoma Could Be Efficacious Targets. Cancers 2019, 11 (9), 1308 10.3390/cancers11091308. 31491891 PMC6771132

[ref56] Billington R. A.; Genazzani A. A.; Travelli C.; Condorelli F. NAD Depletion by FK866 Induces Autophagy. Autophagy 2008, 4 (3), 385–387. 10.4161/auto.5635. 18227641

[ref57] Lucena-Cacace A.; Otero-Albiol D.; Jiménez-García M. P.; Muñoz-Galvan S.; Carnero A. NAMPT Is a Potent Oncogene in Colon Cancer Progression That Modulates Cancer Stem Cell Properties and Resistance to Therapy through Sirt1 and PARP. Clin. Cancer Res. 2018, 24 (5), 1202–1215. 10.1158/1078-0432.CCR-17-2575. 29203587

[ref58] Ross G. J.; Foulkes A. S. MixMAP: An R Package for Mixed Modeling of Meta-Analysis p Values in Genetic Association Studies. J. Stat. Soft. 2015, 66, 1–20. 10.18637/jss.v066.i03.

[ref59] Vichai V.; Kirtikara K. Sulforhodamine B Colorimetric Assay for Cytotoxicity Screening. Nat. Protoc. 2006, 1 (3), 1112–1116. 10.1038/nprot.2006.179. 17406391

[ref60] Hopkins S. L.; Siewert B.; Askes S. H. C.; Veldhuizen P.; Zwier R.; Heger M.; Bonnet S. An in Vitro Cell Irradiation Protocol for Testing Photopharmaceuticals and the Effect of Blue, Green, and Red Light on Human Cancer Cell Lines. Photochem. Photobiol. Sci. 2016, 15 (5), 644–653. 10.1039/c5pp00424a. 27098927 PMC5044800

[ref61] Zhang N.; Fu J.-N.; Chou T.-C. Synergistic Combination of Microtubule Targeting Anticancer Fludelone with Cytoprotective Panaxytriol Derived from Panax Ginseng against MX-1 Cells in Vitro: Experimental Design and Data Analysis Using the Combination Index Method. Am. J. Cancer Res. 2015, 6 (1), 97–104. 27073727 PMC4759401

