# Supplementary material for: Oxygen-Dependent Interactions between the Ruthenium Cage and the Photoreleased Inhibitor in NAMPT-Targeted Photoactivated Chemotherapy
Source: J Med Chem. 2024 Jun 26;67(13):11086–102. doi: 10.1021/acs.jmedchem.4c00589 (PMC11247496; doi:10.1021/acs.jmedchem.4c00589)
Supplement: Supplementary file 1 — jm4c00589_si_001.pdf [file jm4c00589_si_001.pdf]

Supporting information

of

Oxygen-dependent interactions between the ruthenium cage and the photoreleased inhibitor in NAMPT-targeted photoactivated chemotherapy

Selda Abyar[1,2], Luojiao Huang[2], Yurii Husiev[1], Ludovic Bretin[1], Bobby Chau[1,2], Vadde Ramu[1], Jacob Hendricus Wildeman [2], Kimberley Belfor[2], Lukas S. Wijaya [2], Vera E. vander Noord[2], Amy C. Harms[2], Maxime A. Siegler[3], Sylvia E. Le Dévédec[2,✉], Sylvestre Bonnet[1,✉]

[1] Leiden Institute of Chemistry, Leiden University, Gorlaeus Laboratories, PO Box 9502, 2300 RA Leiden (The Netherlands)
[2] Leiden Academic Centre for Drug Research, Leiden University, Gorlaeus Laboratories, PO Box 9502, 2300 RA Leiden (The Netherlands)
[3] Department of Chemistry, Johns Hopkins University, 3400 N Charles St., Baltimore, MD, 21218 (USA)

Table of Contents

NMR data 2
2. Mass spectrometry data 12
3. Single crystal X-ray crystallography 12
4. CompuSyn Report in Normoxic and Hypoxic U87MG (Figure S21) 15
5. HPLC analysis 20
6. References 21

NMR data

1H NMR: 4-((((benzyloxy)carbonyl)amino)methyl)benzoic acid (1)

^{1}H NMR (400 MHz, DMSO-d6) δ 12.90 (s, 1H), 7.96 – 7.86 (m, 3H), 7.41 – 7.27 (m, 7H), 5.05 (s, 2H), 4.27 (d, *J* = 6.2 Hz, 2H).

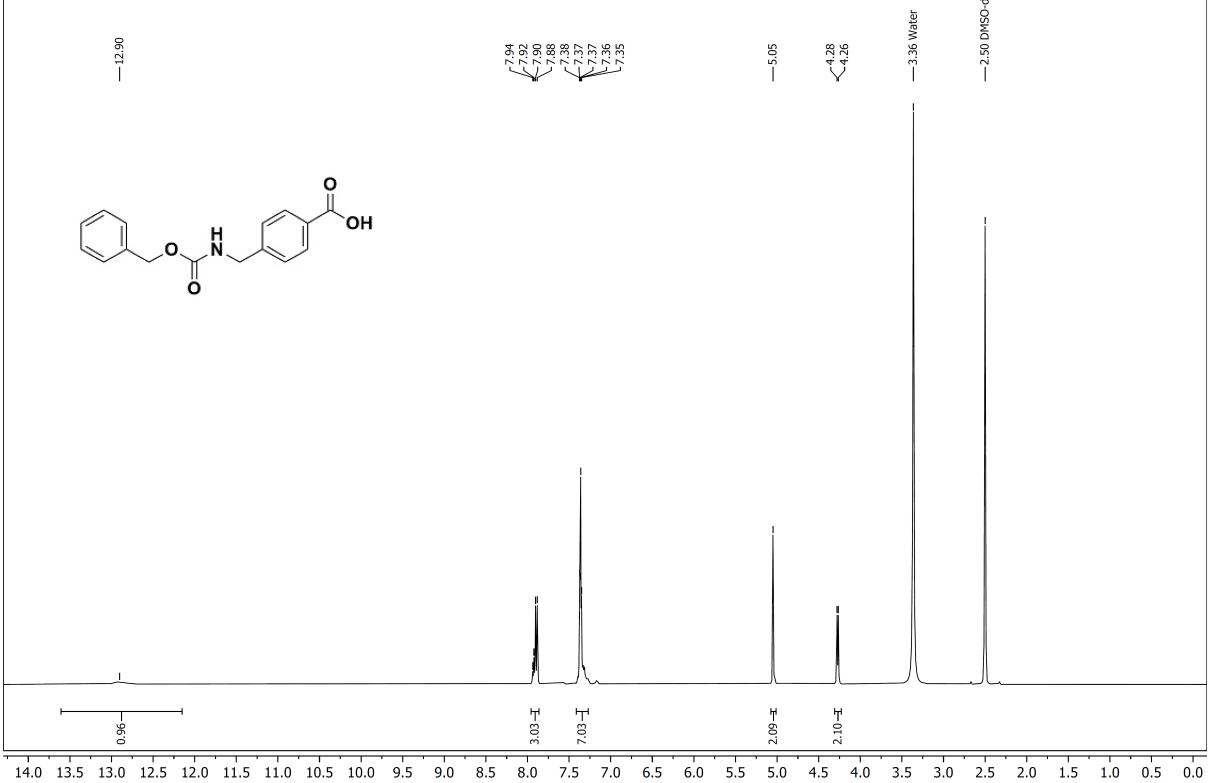

Figure S1. ^{1}H NMR of 4-((((benzyloxy)carbonyl)amino)methyl)benzoic acid (**1**).

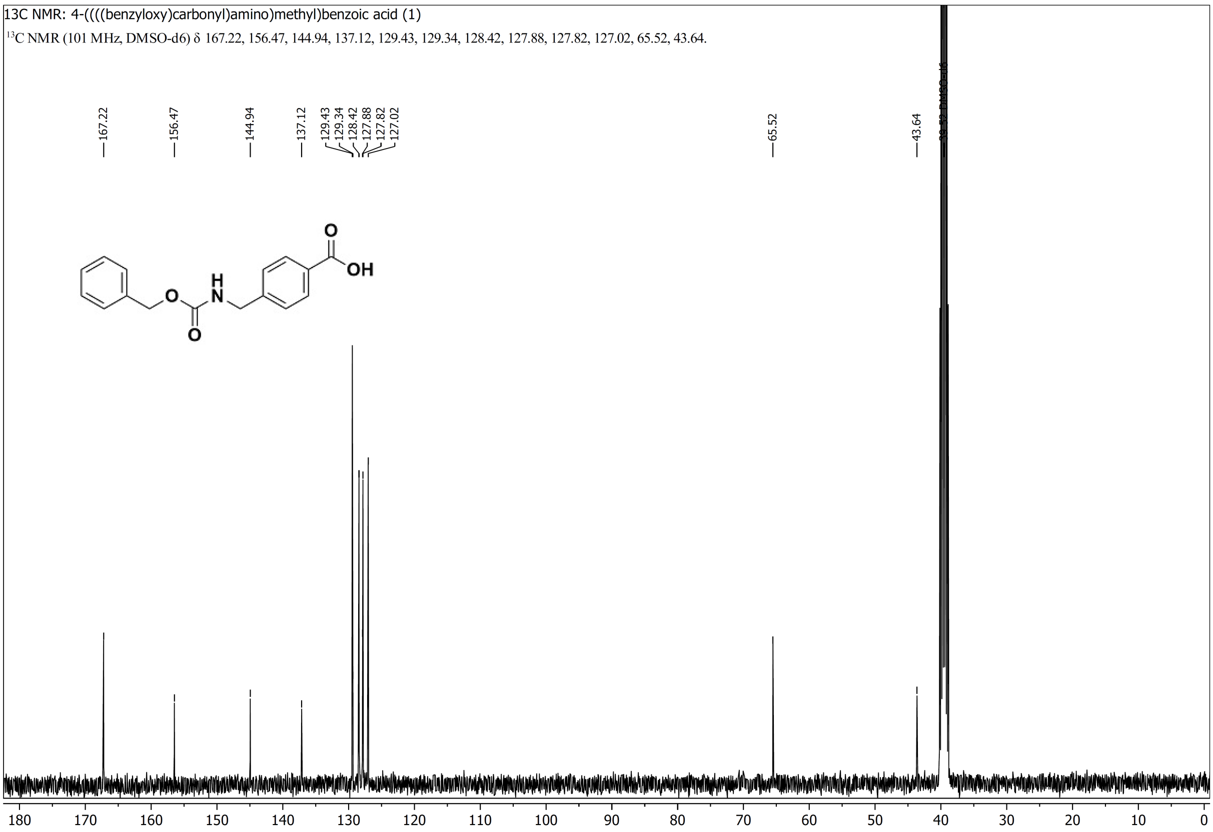

Figure S2. ^{13}C NMR of 4-((((benzyloxy)carbonyl)amino)methyl)benzoic acid (**1**).

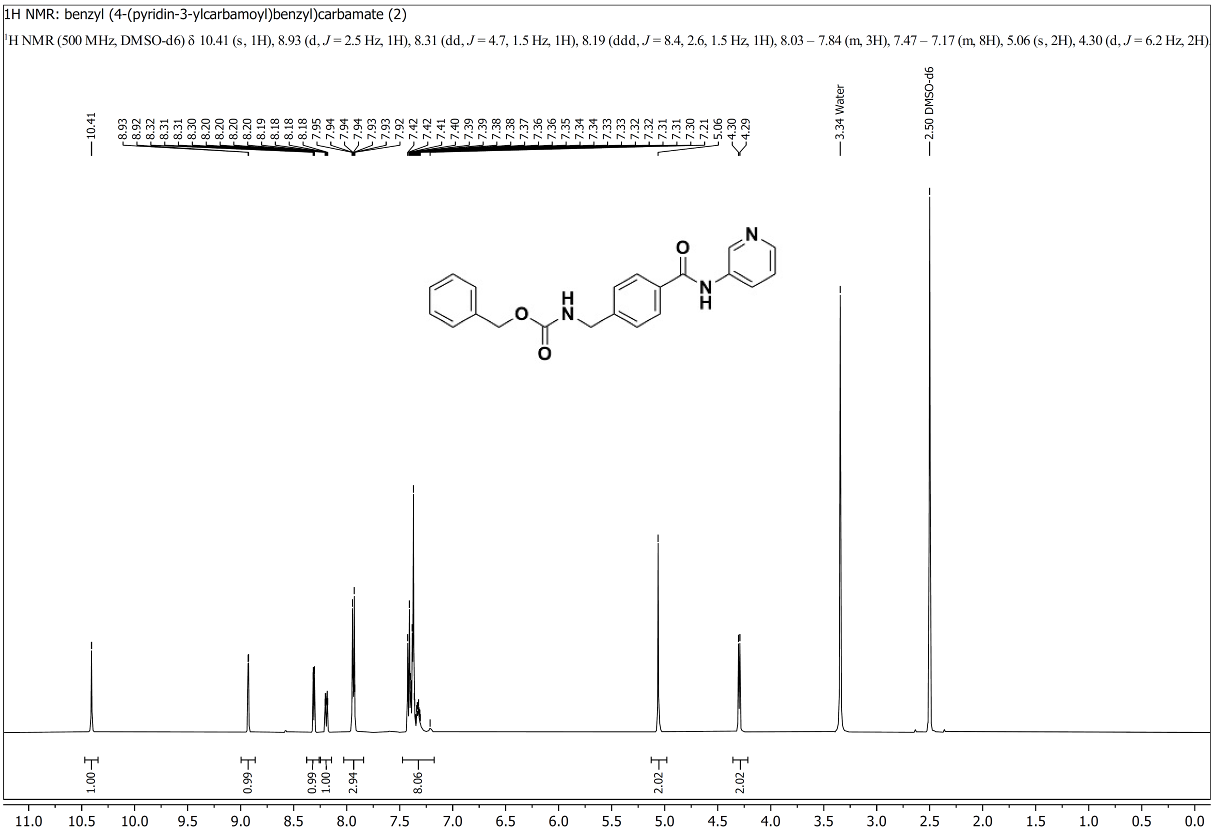

Figure S3. ^{1}H NMR of benzyl (4-(pyridin-3-ylcarbamoyl)benzyl)carbamate (**2**).

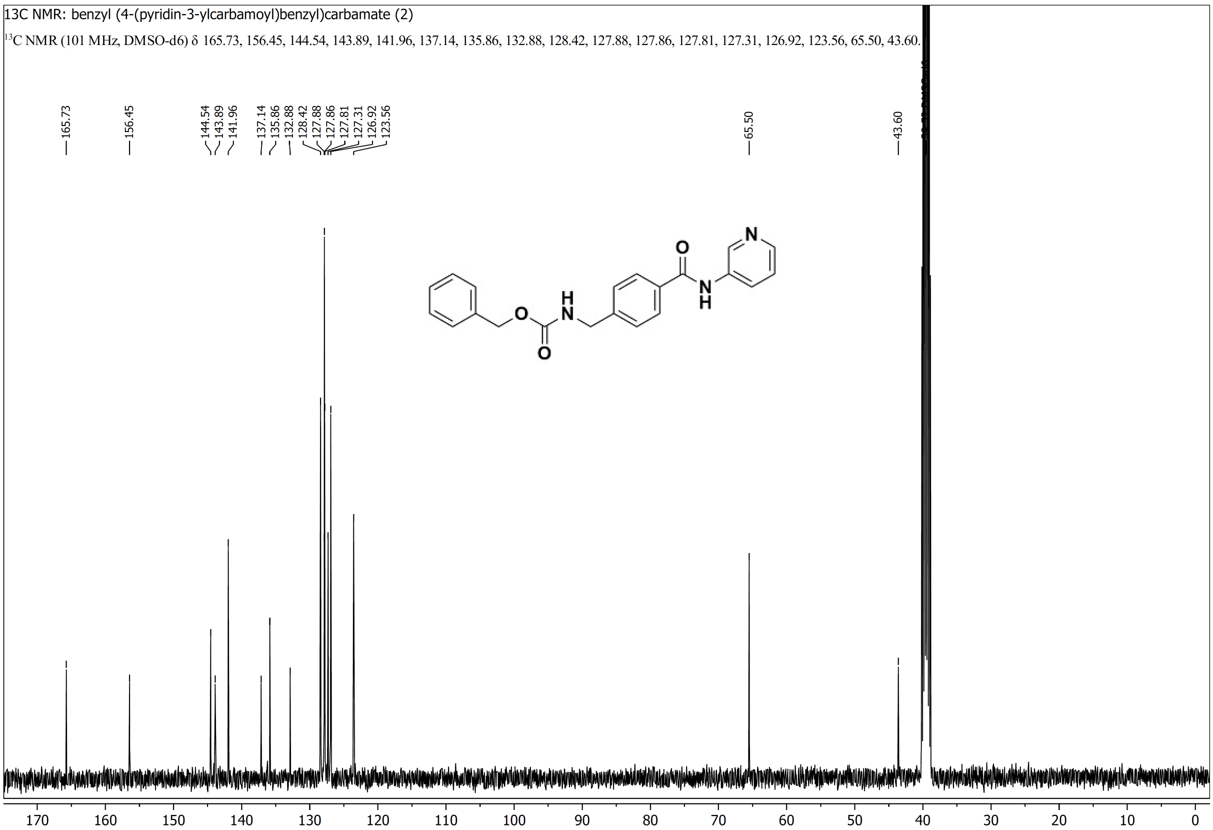

Figure S4. ^{13}C NMR of benzyl (4-(pyridin-3-ylcarbamoyl)benzyl)carbamate (**2**).

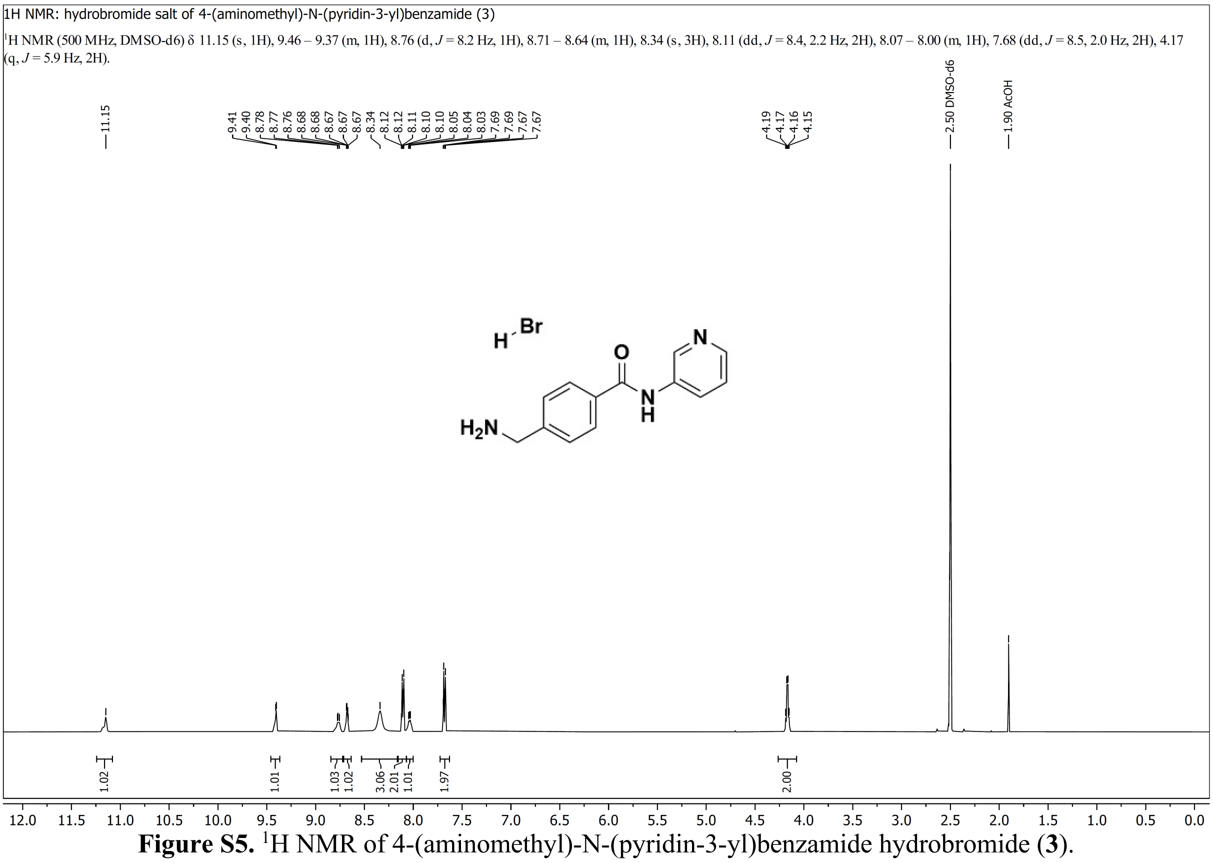

Figure S5. ^{1}H NMR of 4-(aminomethyl)-N-(pyridin-3-yl)benzamide hydrobromide (**3**).

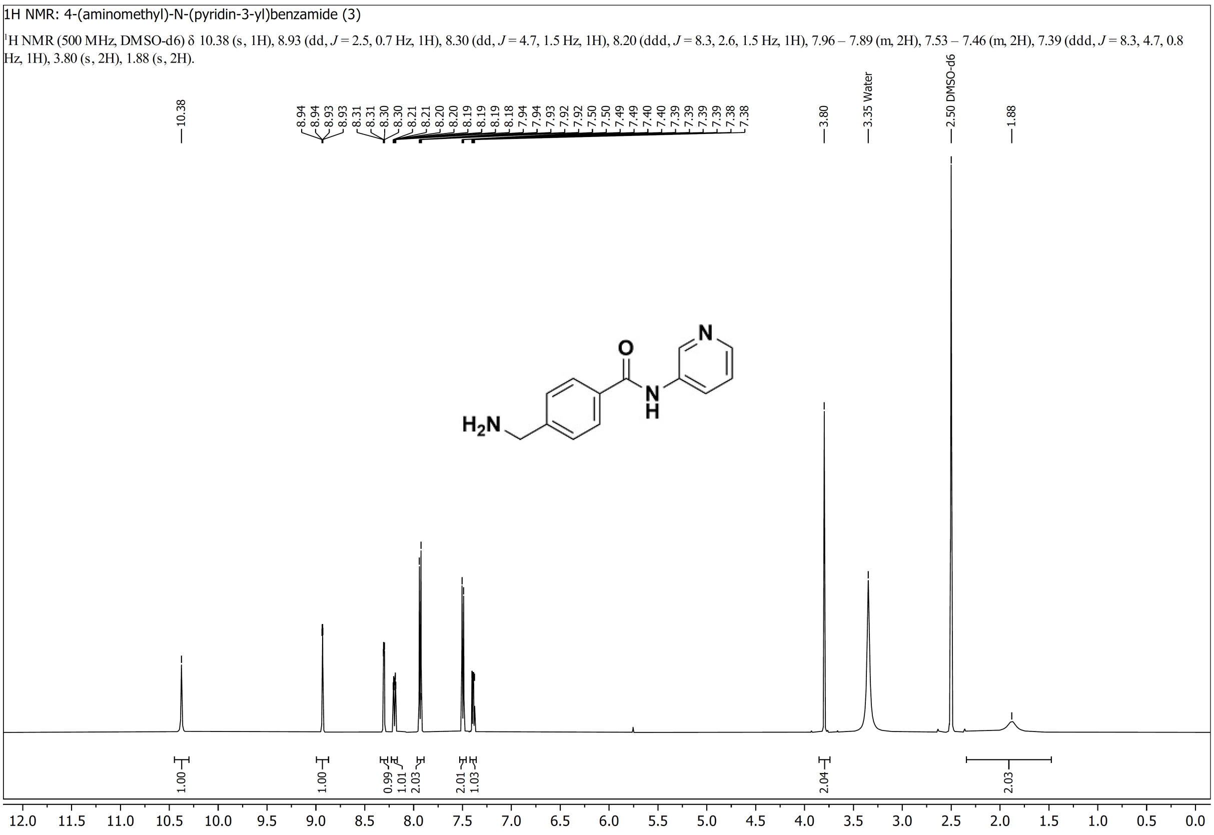

Figure S6. ^{1}H NMR of 4-(aminomethyl)-N-(pyridin-3-yl)benzamide (**3**).

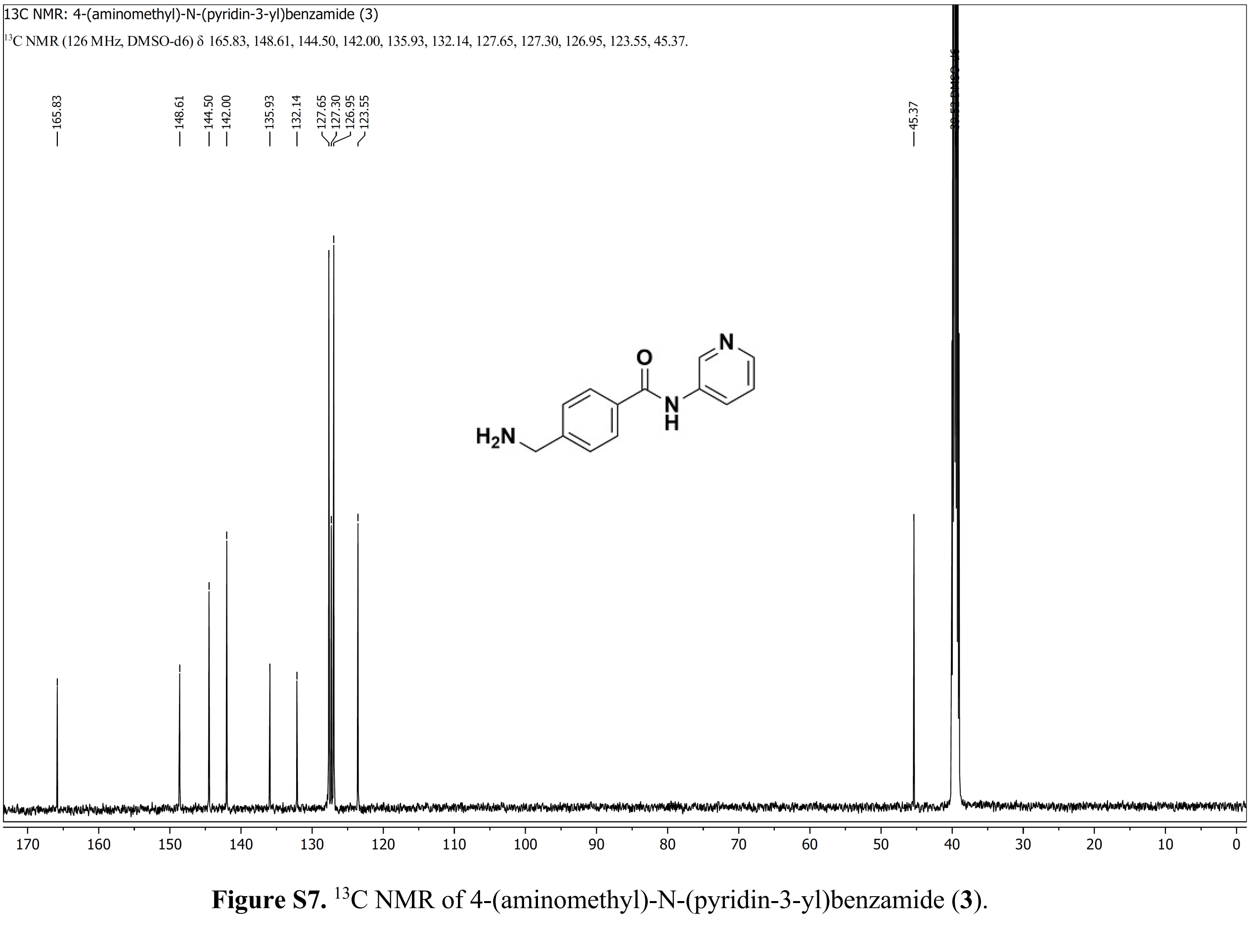

Figure S7. ^{13}C NMR of 4-(aminomethyl)-N-(pyridin-3-yl)benzamide (**3**).

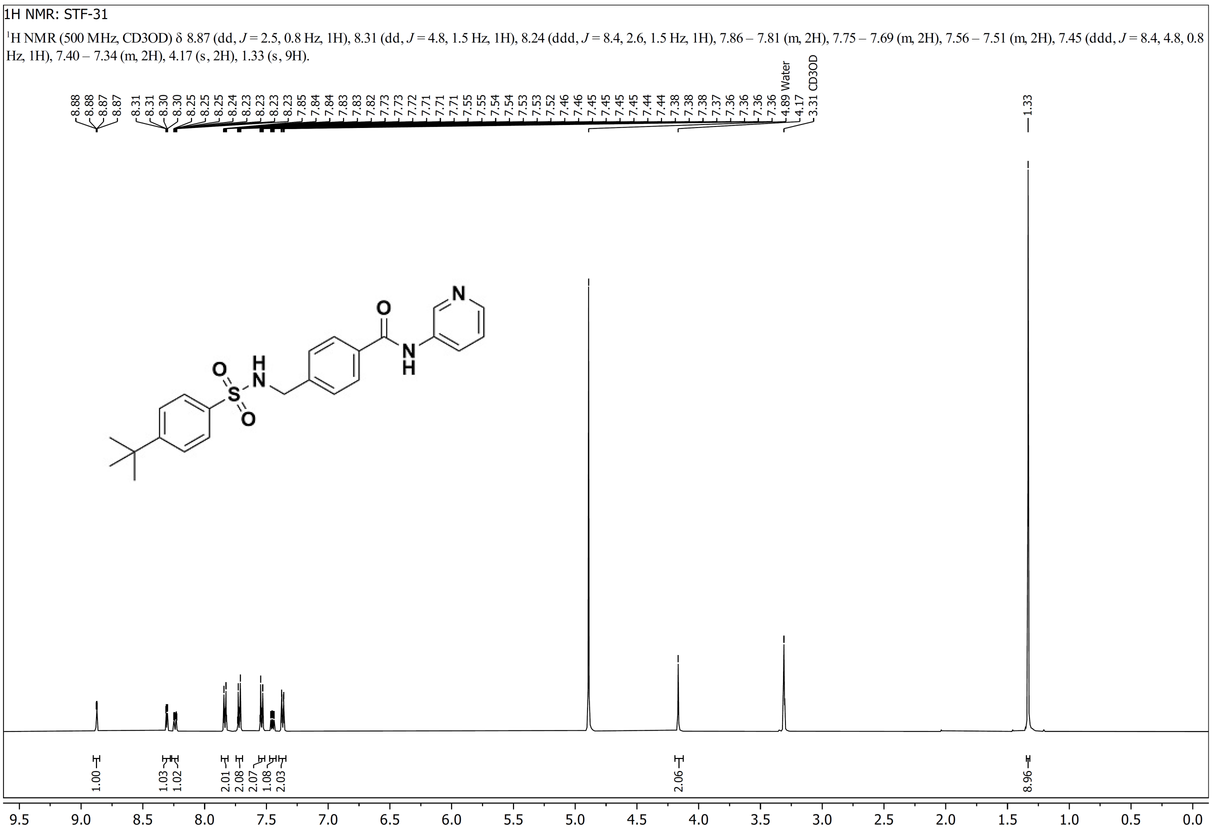

Figure S8. ^{1}H NMR of **STF-31**.

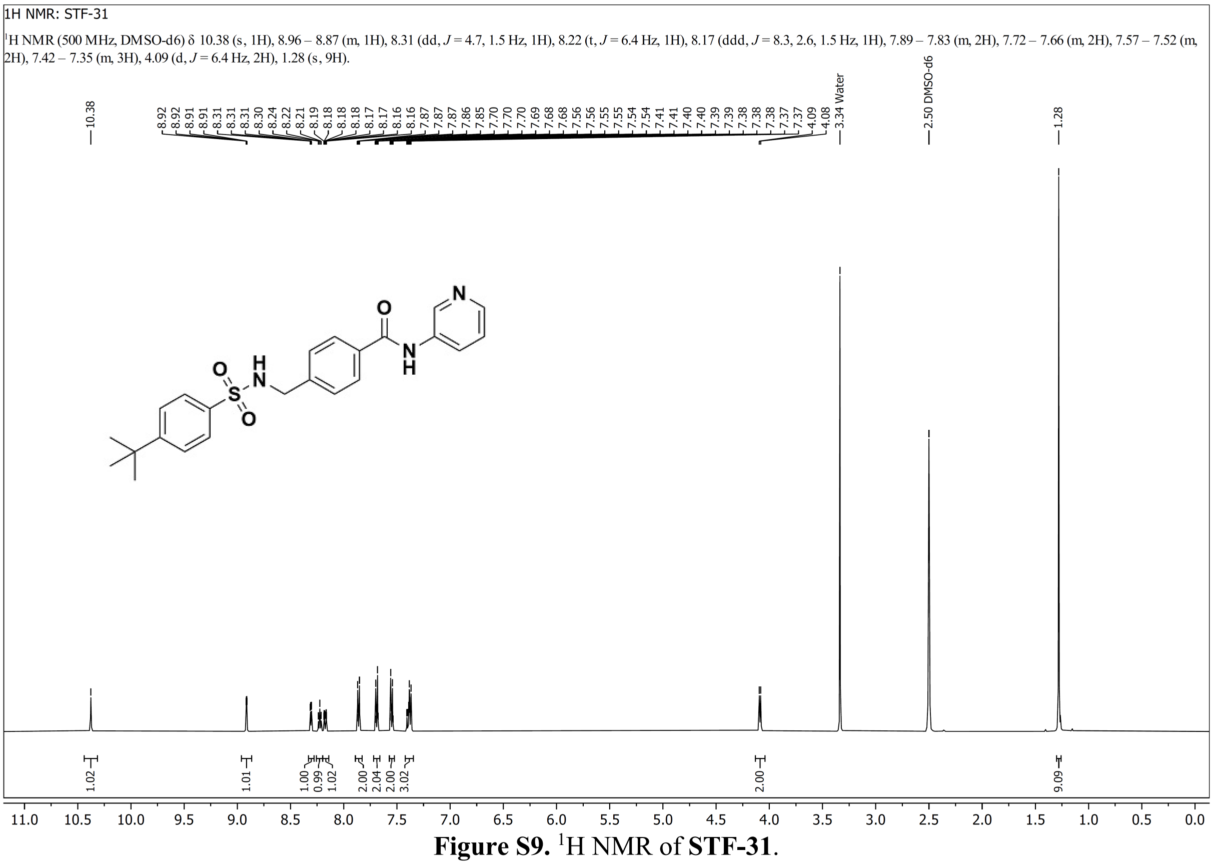

Figure S9. ^{1}H NMR of **STF-31**.

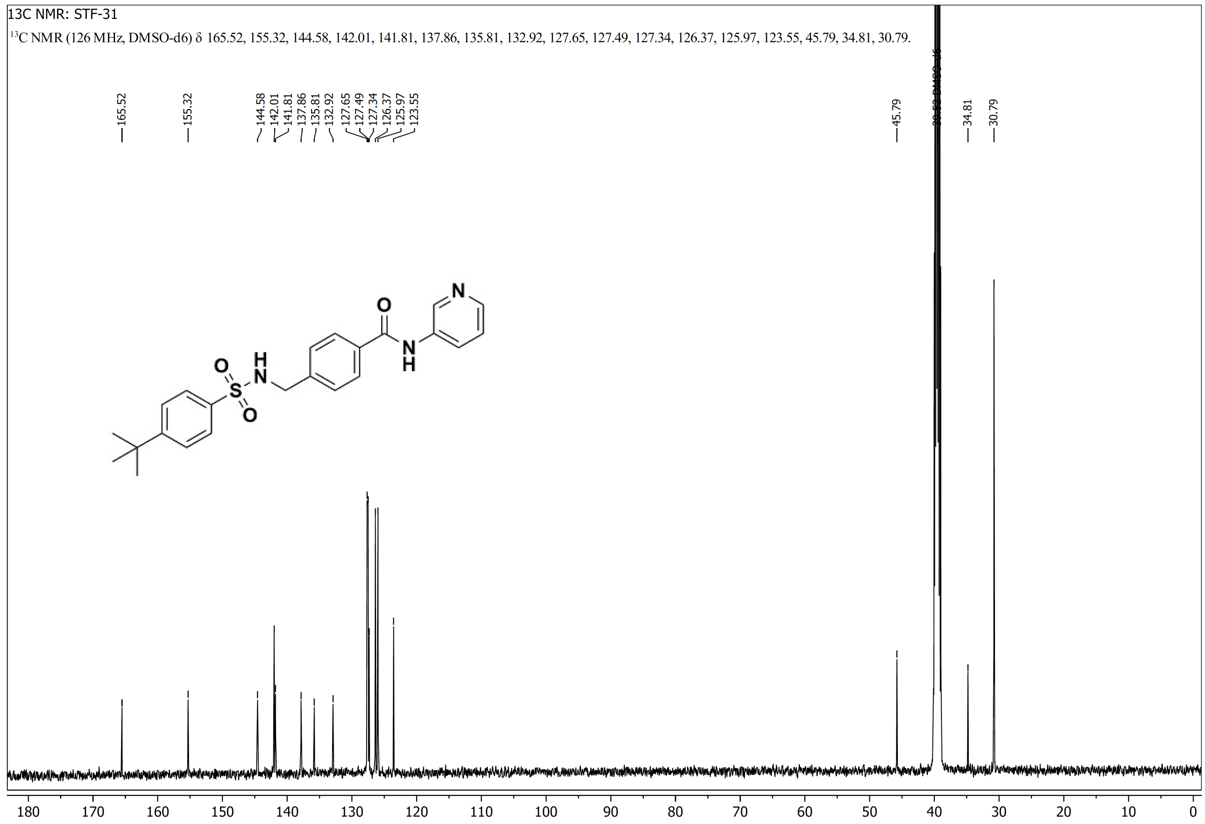

Figure S10. ^{13}C NMR of **STF-31**

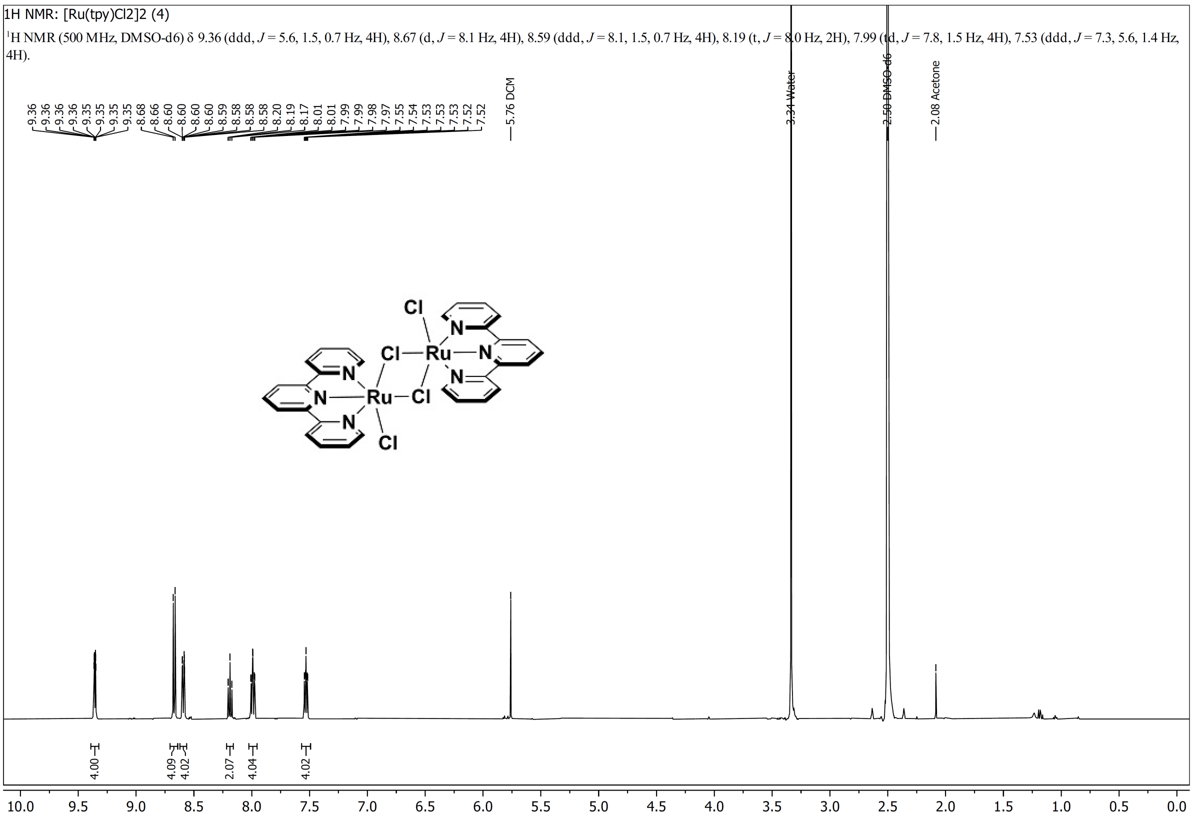

Figure S11. ^{1}H NMR of $[Ru(tpy)Cl_2]_2$ (**4**).

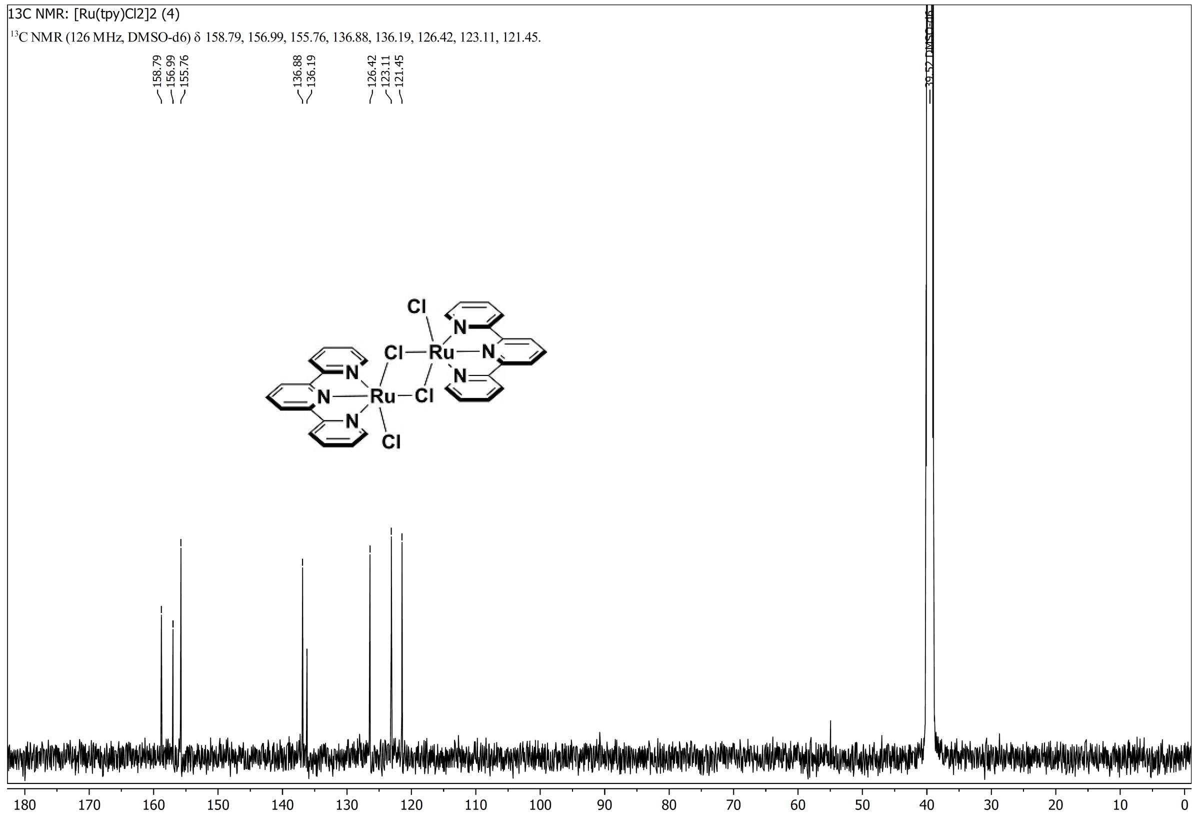

Figure S12. ^{13}C NMR of $[Ru(tpy)Cl_2]_2$ (**4**).

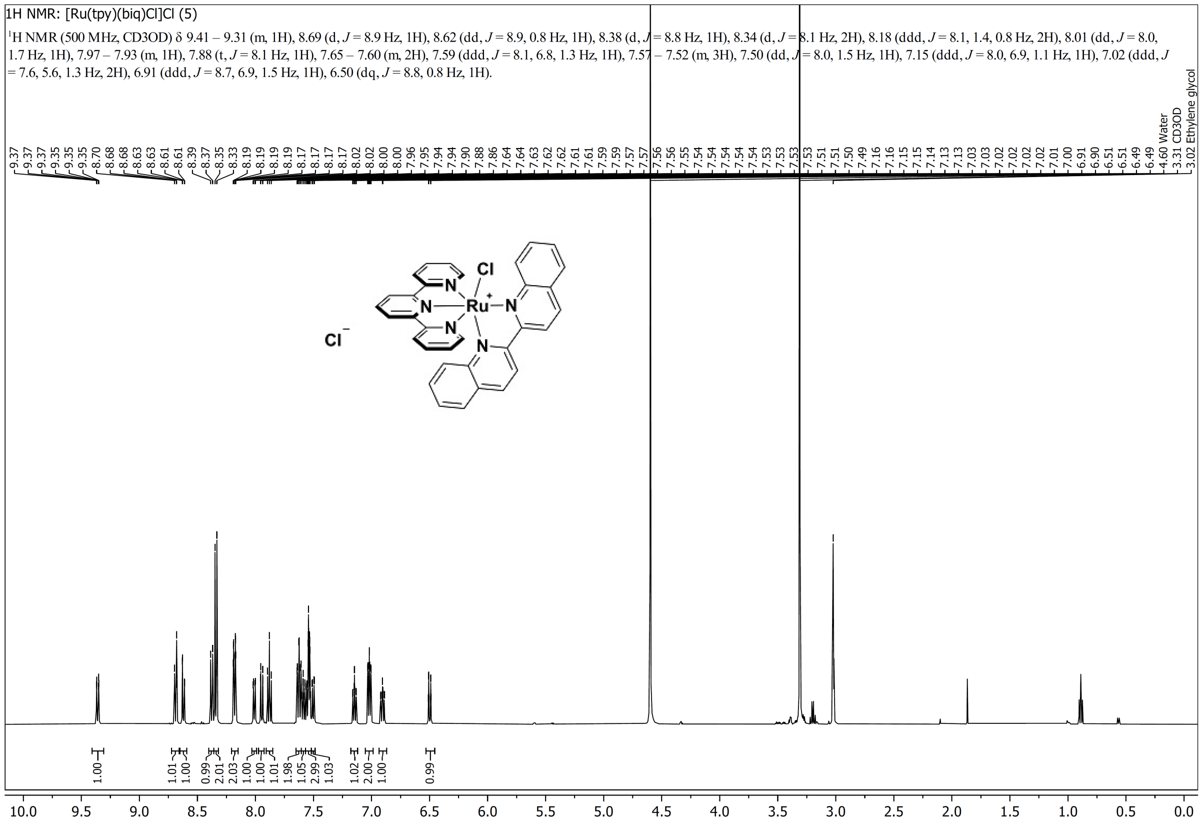

Figure S13. ^{1}H NMR of [Ru(tpy)(biq)Cl]Cl (**5**).

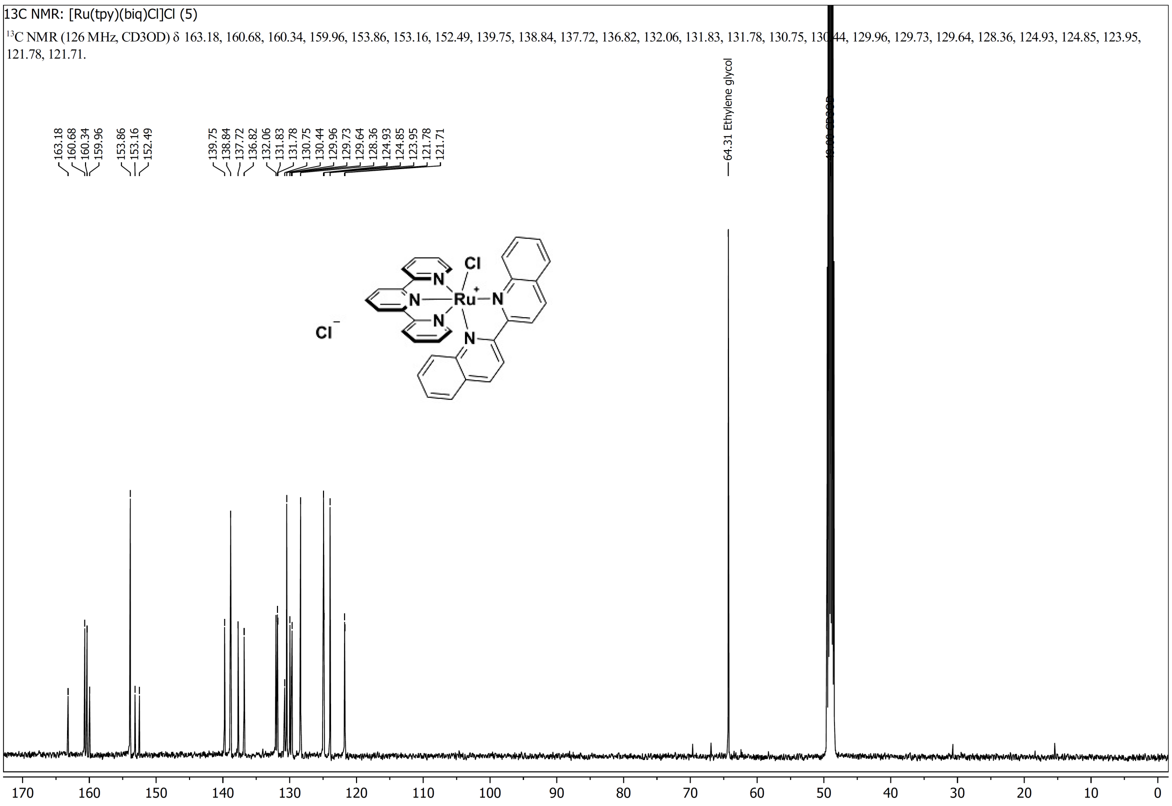

Figure S14. ^{13}C NMR of [Ru(tpy)(biq)Cl]Cl (**5**).

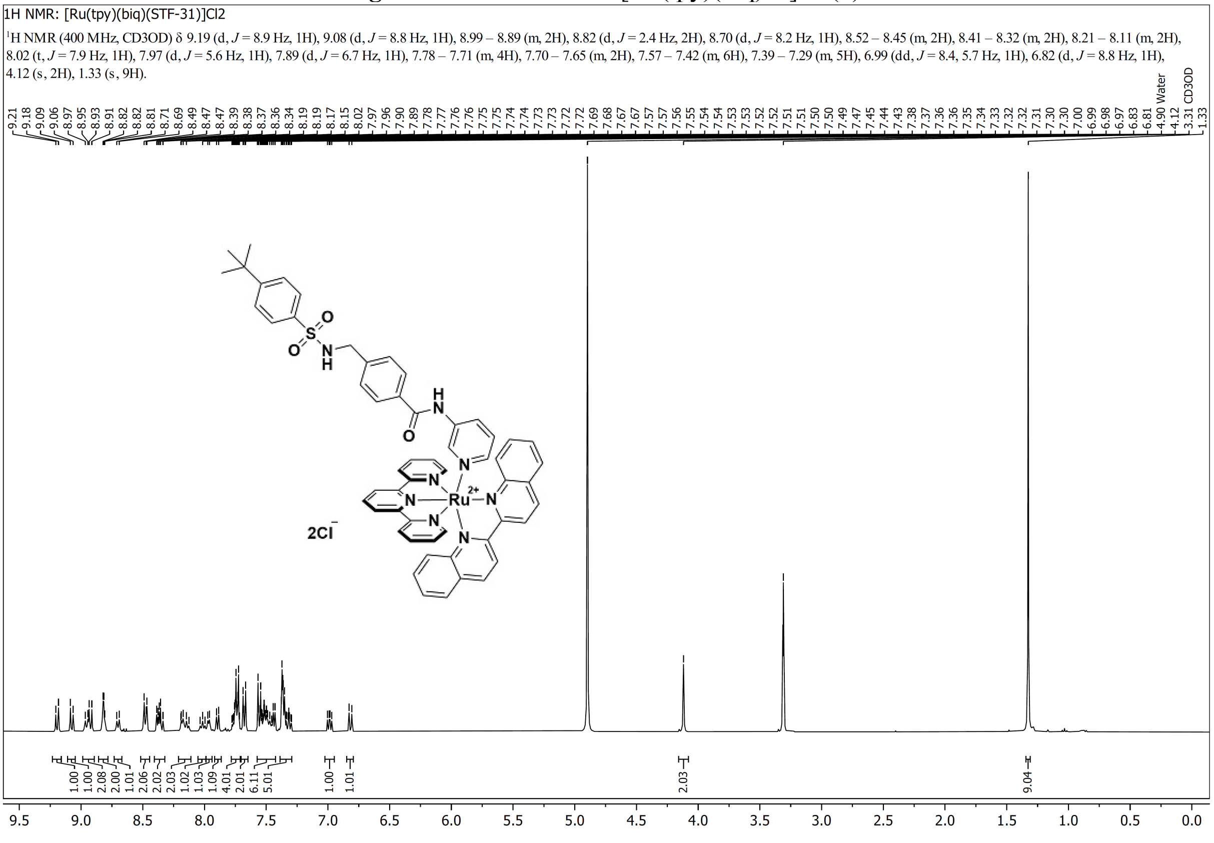

Figure S15. ^{1}H NMR of [Ru(tpy)(biq)(STF-31)]Cl_2 (**Ru-STF-31**).

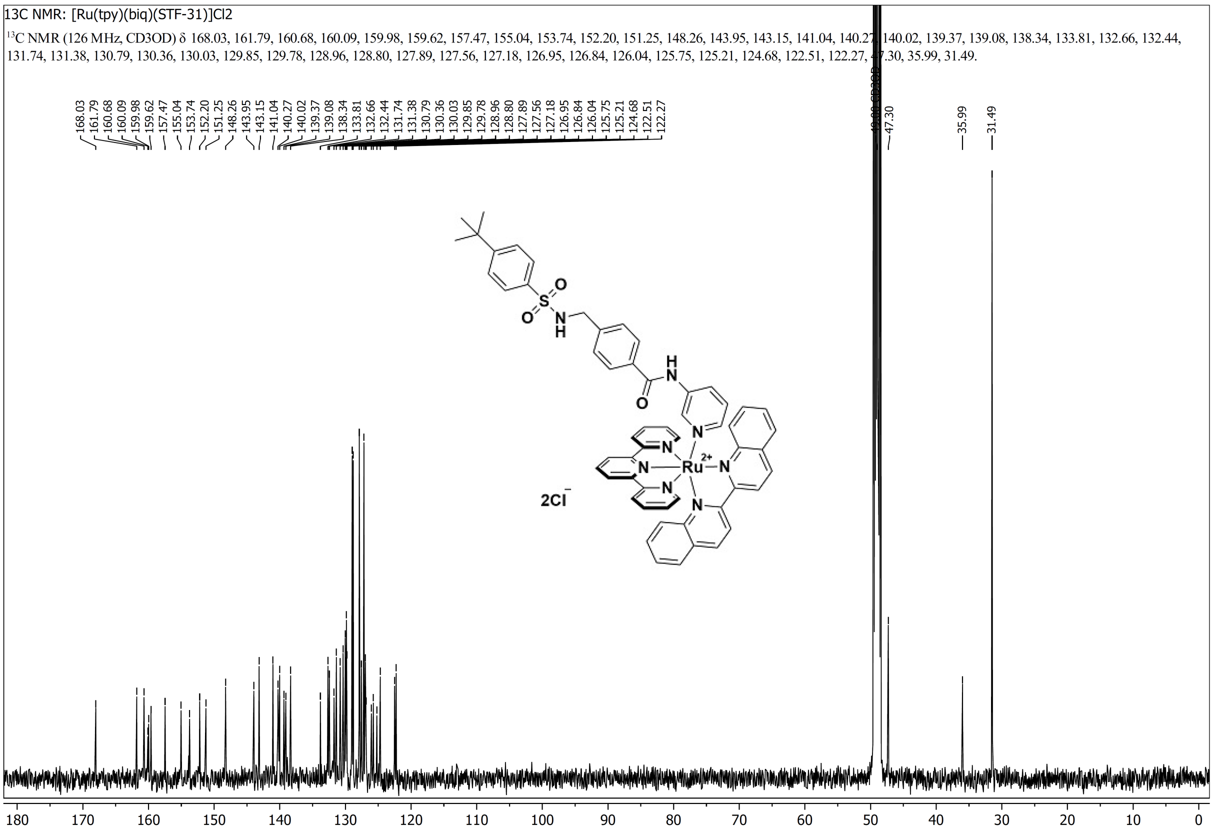

Figure S16. ^{13}C NMR of [Ru(tpy)(biq)(STF-31)]Cl_2 (**Ru-STF-31**).

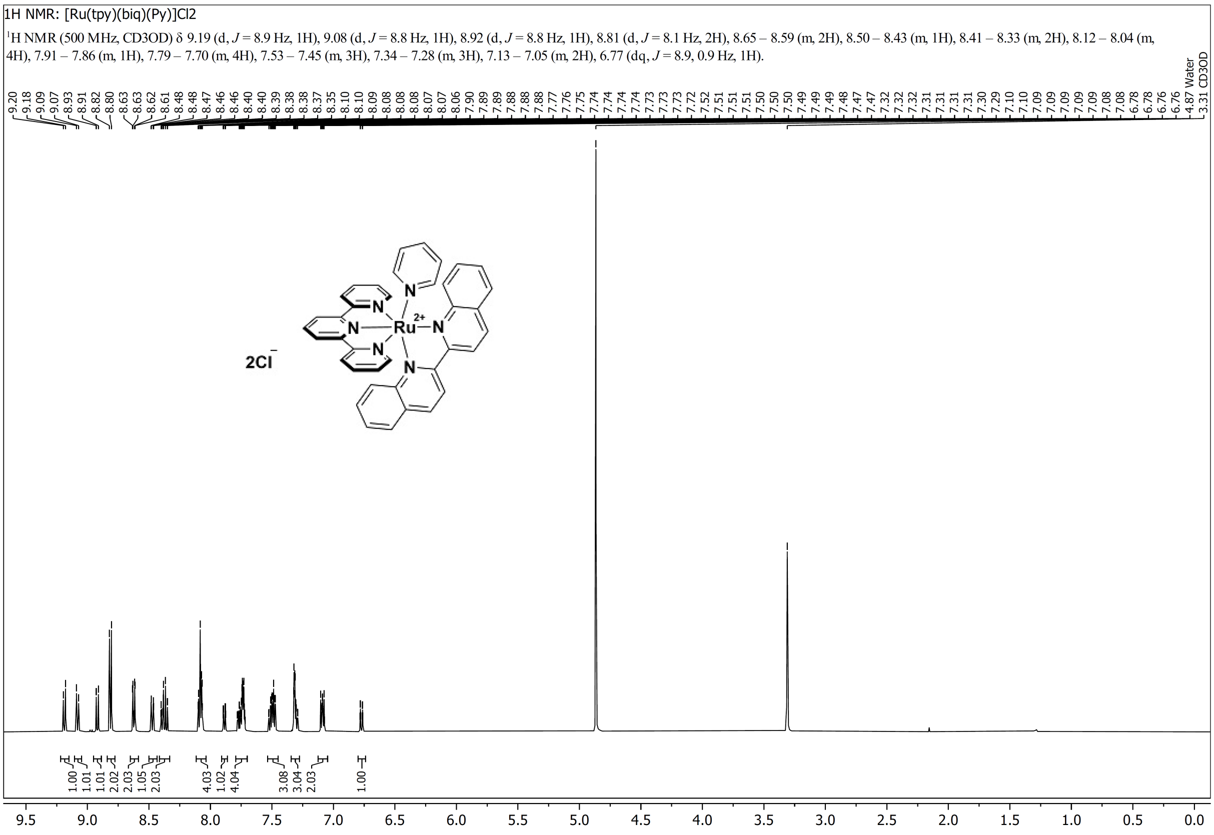

Figure S17. ^{1}H NMR of [Ru(tpy)(biq)(Py)]Cl_2 (**Ru-Py**).

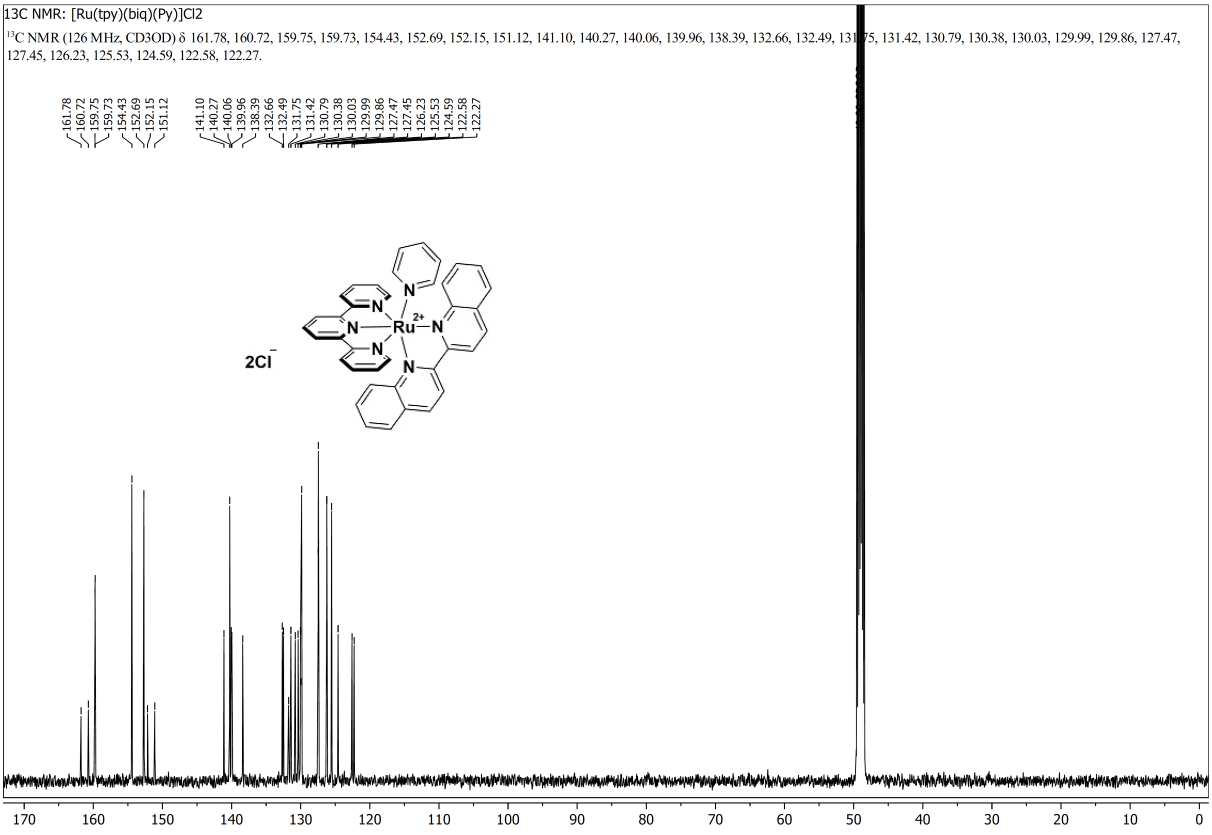

Figure S18. ^{13}C NMR of [Ru(tpy)(biq)(Py)]Cl_2 (**Ru-Py**).

2. Mass spectrometry data

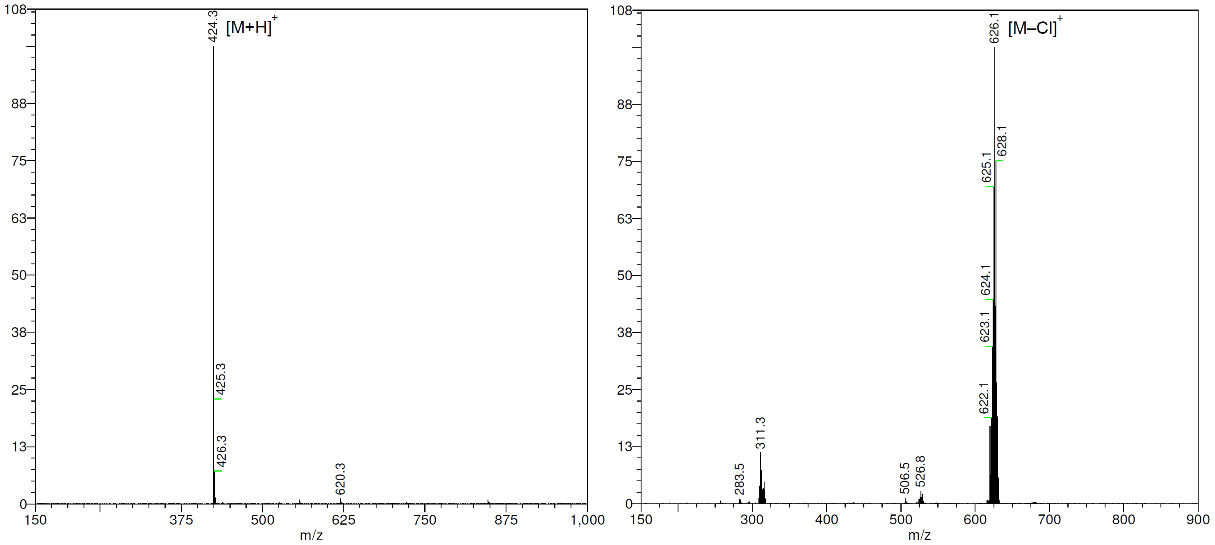

Figure S19. ESI-MS of **STF-31** (left) and [Ru(tpy)(biq)Cl]Cl (**5**) (right).

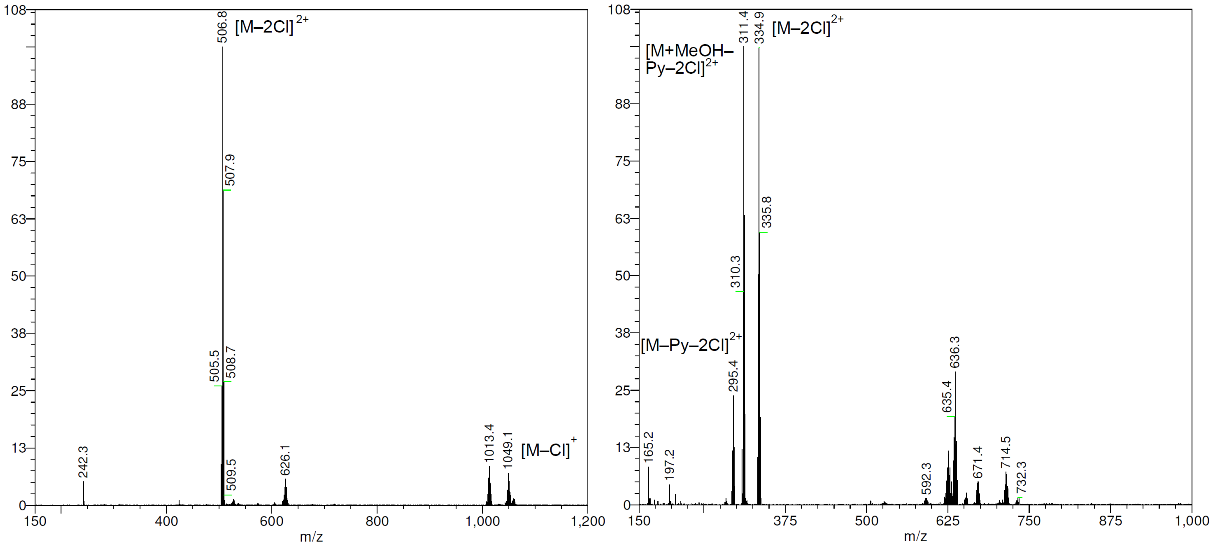

Figure S20. ESI-MS of [Ru(tpy)(biq)(STF-31)]Cl_2 (**Ru-STF-31**) (left) and [Ru(tpy)(biq)(Py)]Cl_2 (**Ru-Py**) (right).

3. Single crystal X-ray crystallography

*[Ru(tpy)(biq)(STF-31)]Cl_2 (**Ru-STF-31**)*

All reflection intensities were measured at 110(2) K using a SuperNova diffractometer (equipped with Atlas detector) with Cu *K*α radiation (λ = 1.54178 Å) under the program CrysAlisPro (Version CrysAlisPro 1.171.42.49, Rigaku OD, 2022). The same program was used to refine the cell dimensions and for data reduction. The structure was solved with the program SHELXS-2018/3 (Sheldrick, 2018) and was refined on F^2 with SHELXL-2018/3 (Sheldrick, 2018). Analytical numeric absorption correction using a multifaceted crystal model was applied using CrysAlisPro. The temperature of the data collection was controlled using the system Cryojet (manufactured by Oxford Instruments). The H atoms were placed at calculated positions (unless otherwise specified) using the instructions AFIX 23, AFIX 43 or AFIX 137

with isotropic displacement parameters having values 1.2 or 1.5 U_{eq} of the attached C or N atoms. The H atom attached to N7 was found from difference Fourier map, and its coordinates were refined pseudo freely using the DFIX instruction in order to keep the N−H bond distance within an acceptable range.

The structure is partly disordered. The moiety C40 > C56 and one of the two BF_4^- counterions are disordered over two orientations and the occupancy factors of the major components are refined to 0.748(3) and 0.799(12), respectively. The other BF_4^- counterion is found to be disordered over four different orientations at two different sites in the asymmetric unit, and the four components are refined to 0.302(3), 0.300(3), 0.092(2) and 0.306(3). The asymmetric unit contains a small amount of very disordered lattice solvent molecule, and that contribution was removed from the final refinement using the SQUEEZE procedure in Platon (Spek, 2009)[3].

Computer programs: *CrysAlis PRO* 1.171.42.49 (Rigaku OD, 2022), *SHELXS2018*/3 (Sheldrick, 2018), *SHELXL2018*/3 (Sheldrick, 2018), *SHELXTL* v6.10 (Sheldrick, 2008) [4]. The X-ray structure has been deposited in the Cambridge Crystallographic Data Center and can be retrieved there under the reference CCDC 2356006.

Table S1. Crystallographic data summary for [Ru(tpy)(biq)(STF-31)]Cl_2 (**Ru-STF-31**).

**Crystal data**	
Chemical formula	$C_{56}H_{48}N_8O_3RuS·2(BF_4)$
M_r	1187.77
Crystal system, space group	Triclinic, *P*-1
Temperature (K)	110
a, *b*, *c* (Å)	13.3211 (4), 14.2695 (4), 15.9077 (4)
α, β, γ (°)	73.389 (2), 89.758 (2), 67.568 (3)
V (Å^3)	2659.54 (14)
Z	2
Radiation type	Cu *K*α
μ (mm^{-1})	3.45
Crystal size (mm)	0.10 × 0.06 × 0.02
**Data collection**	
Diffractometer	SuperNova, Dual, Cu at zero, Atlas
Absorption correction	Analytical<br>*CrysAlis PRO* 1.171.42.49 (Rigaku Oxford Diffraction, 2022)<br>Analytical numeric absorption correction using a multifaceted crystal model based on expressions derived by R.C. Clark & J.S. Reid. (Clark, R. C. & Reid, J. S. (1995). Acta Cryst. A51, 887-897)<br>Empirical absorption correction using spherical harmonics, implemented in SCALE3 ABSPACK scaling algorithm.
T_{min}, T_{max}	0.755, 0.936
No. of measured, independent and	36111, 10403, 9061

observed [$I > 2\sigma(I)$] reflections	
R_{int}	0.039
$(\sin \theta/\lambda)_{max}$ (Å^{-1})	0.616
**Refinement**	
$R[F^2 > 2\sigma(F^2)]$, $wR(F^2)$, S	0.041, 0.114, 1.05
No. of reflections	10403
No. of parameters	1081
No. of restraints	1315
H-atom treatment	H atoms treated by a mixture of independent and constrained refinement
$\Delta\rho_{max}$, $\Delta\rho_{min}$ (e Å^{-3})	0.87, -1.01

Table S2. Selected bond lengths and angles for [Ru(tpy)(biq)(STF-31)]Cl_2 (**Ru-STF-31**).

**Bonds, Å**		**Angles, °**	
Ru1–N1	2.065(2)	N1–Ru1–N2	79.6(1)
Ru1–N2	1.965(2)	N1–Ru1–N4	101.6(1)
Ru1–N3	2.098(3)	N1–Ru1–N5	97.5(1)
Ru1–N4	2.107(2)	N1–Ru1–N6	92.4(1)
Ru1–N5	2.099(3)	N2–Ru1–N3	79.2(1)
Ru1–N6	2.113(3)	N2–Ru1–N5	99.1(1)
		N2–Ru1–N6	87.4(1)
		N3–Ru1–N4	99.5(1)
		N3–Ru1–N5	84.5(1)
		N3–Ru1–N6	88.1(1)
		N4–Ru1–N5	77.8(1)
		N4–Ru1–N6	95.6(1)

4. CompuSyn Report in Normoxic and Hypoxic U87MG (Figure S21)

Experiment Name: **Ru-Py(RL)+STF31** in **normoxic U87MG**

Drug: **Ru-Py (RL)** (μM)
Drug: **STF31**(μM)
Drug Combo: **Ru-Py(RL)+STF31(uM)**

Data for Drug: Ru-Py(RL)(μM), n=3

**Dose**	**Effect**
100.0	0.28
50.0	0.46
25.0	0.48
12.5	0.69
6.25	0.82
3.12	0.84
1.06	0.99

X-int:	1.49426
Y-int:	1.62420 +/- 0.20158
m: : ***The slope of the median -effect (ME) plot or the shape of dose-response curve***	-1.0870 +/- 0.16138
Dm: ***The median effect dose, in this case it is EC50 value***	31.2077
r: ***The linear correlation coefficient of the ME-plot. An indication of how good are the data, when r=1, it is prefer***	-0.9491

Data for Drug: STF31(μM), n=3

Dose	Effect
100.0	0.53
50.0	0.49
25.0	0.69
12.5	0.88
6.25	0.89
3.12	0.89
1.06	0.99

X-int: 1.84140
Y-int: 1.73141 +/- 0.18454
m: -0.9403 +/- 0.14774
Dm: 69.4059

r: -0.9435

Data for Drug Combo: **Ru-Py(RL)+STF31 (μM),n=3**

Dose A	Effect
100.0+	0.17
50.0+	0.45
25.0+	0.57
12.5+	0.74
6.25+	0.75
3.06+	0.85
1.53+	0.99

X-int: 1.75849
Y-int: 2.09388 +/- 0.31074
m: -1.1907 +/- 0.20430
Dm: 57.3446
r: -0.9336

Dose-Effect Curve

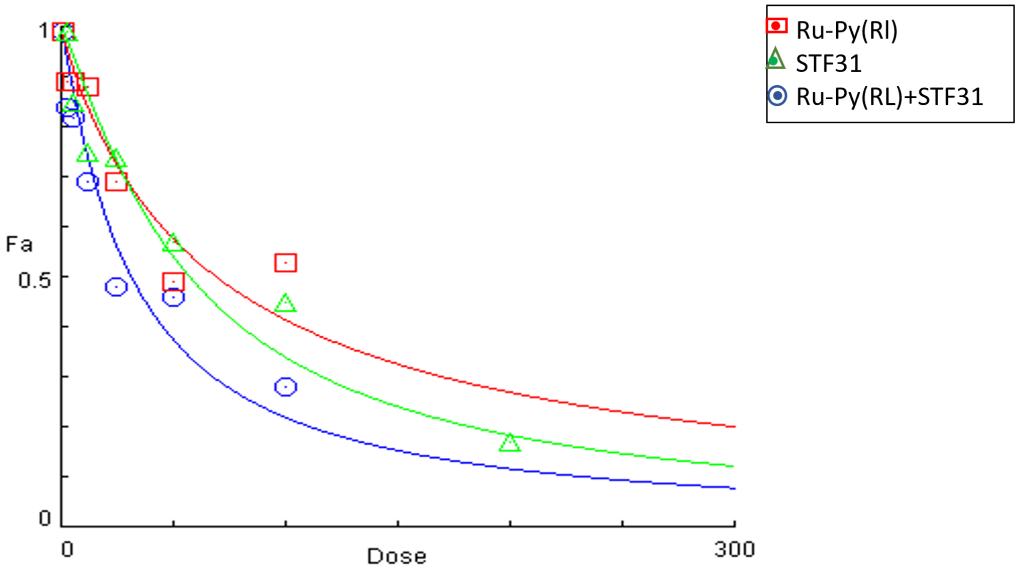

Median-Effect Plot

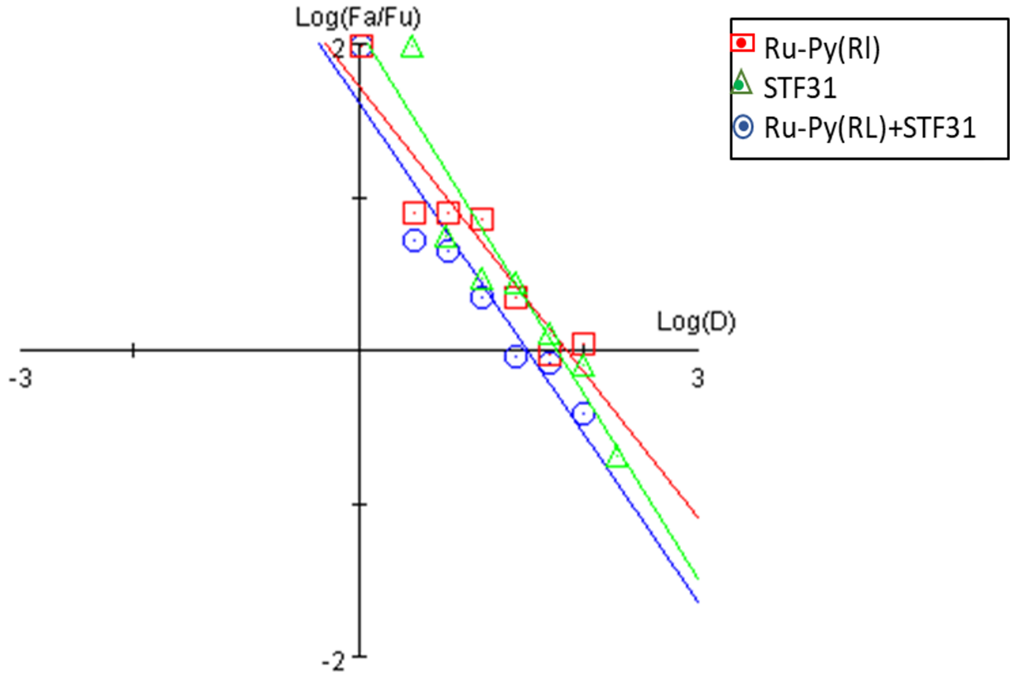

Experiment Name: **Ru-Py(RL)+STF31** in **HypoxicU87MG**

Drug: **Ru-Py (RL)** (µM)
Drug: **STF31**(µM)
Drug Combo: **Ru-Py(RL)+STF31(µM)**

Data for Drug: Ru-Py(RL) (µM),n=3

X-int: 1.895
Y-int: 2.41905 +/- 0.14269
m_1: : -1.2762 +/- 0.11417
Dm_1: 78.06232
r_1: -0.9806

Data for Drug: STF31(µM), n=3

Dose	Effect
100.0	0.6
50.0	0.8
25.0	0.91
12.5	0.92
6.25	0.99

Dose	Effect
3.06	0.99

X-int: 2.15

Y-int: 2.70844 +/- 0.21950

m_2: -1.2596 +/- 0.16275

Dm_2: 141.356

r_2: -0.9682

Data for Drug Combo: **Ru-Py(RL)+STF31 (μM),n=3**

Dose A	Effect
100.0+	0.21
50.0+	044
25.0+	0.63
12.5+	0.82
6.25+	0.93
3.06+	0.93

X-int: 1.89090

Y-int: 2.25052+/- 0.18420

m: -1.1902 +/- 0.11295

Dm: 77.7859

r: -0.9825

Dose-Effect Curve

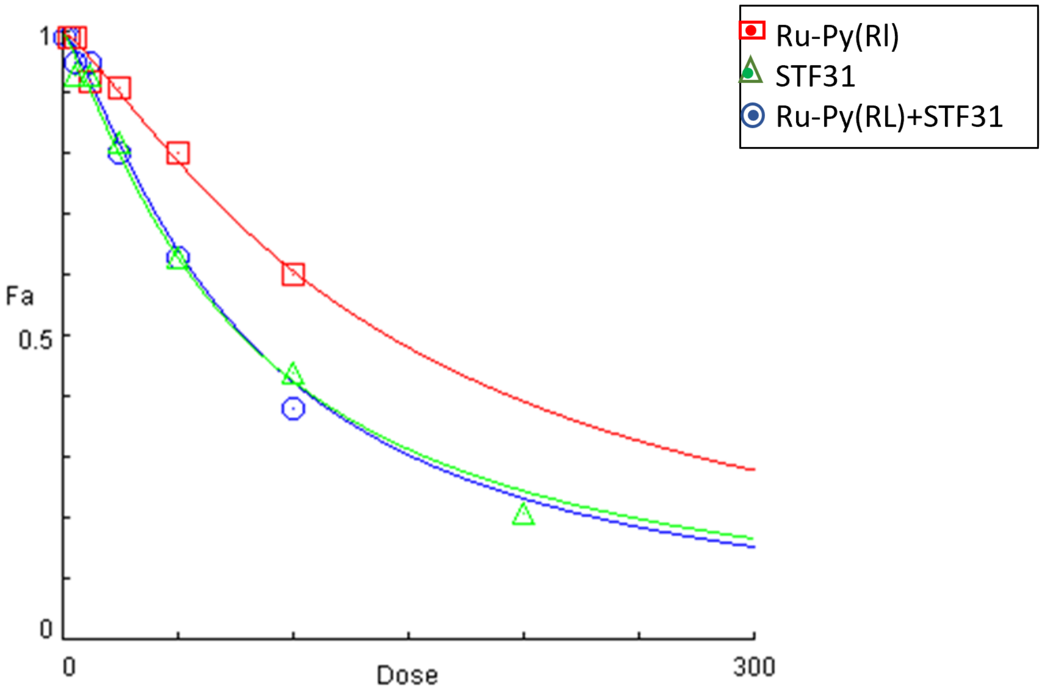

Median-Effect Plot

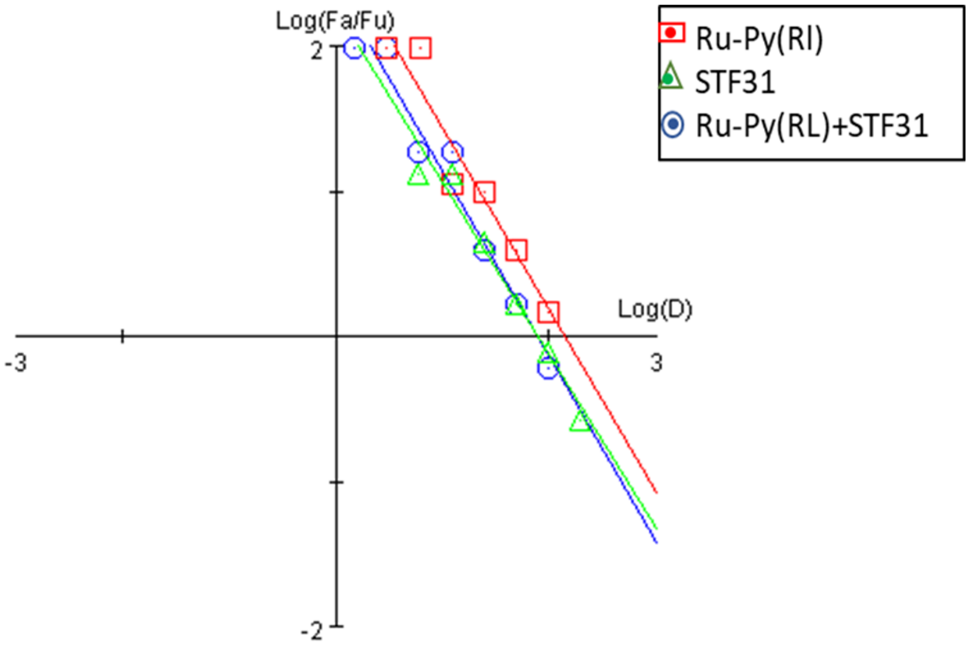

5. HPLC analysis

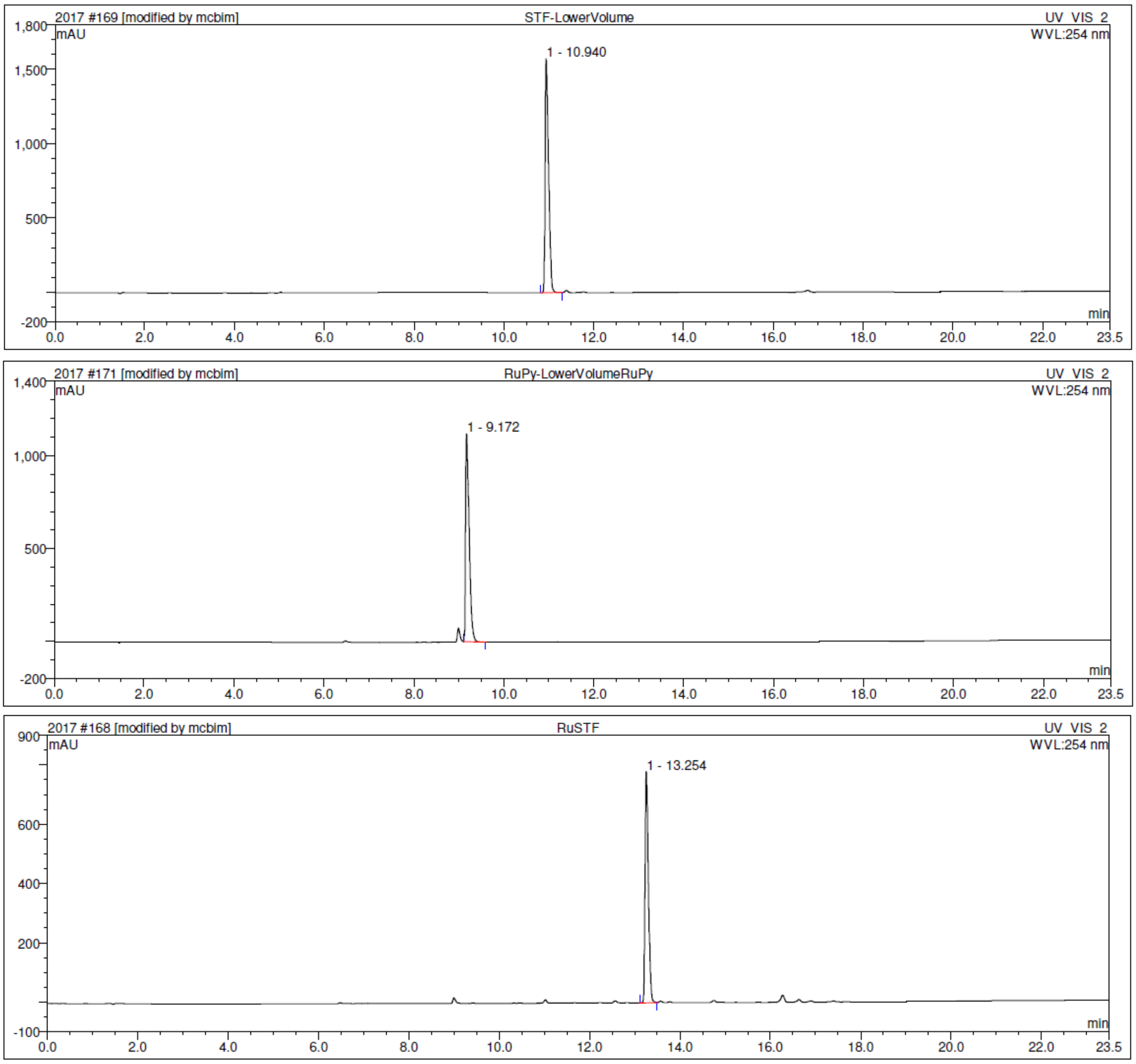

Figure S21. HPLC traces of STF-31 (top, t_R = 10.940 min), [Ru(tpy)(biq)(Py)]Cl_2 (middle, t_R = 9.172 min) and [Ru(tpy)(biq)(STF-31)]Cl_2 (bottom, t_R = 13.254 min); purity is $\geq$ 95%. Gradient elution: 10 to 90% ACN in H_2O (+0.1% v/v formic acid); UV detector: 254 nm; run time: 23.5 min.

6. References

[1] "NMR Chemical Shifts of Trace Impurities: Common Laboratory Solvents, Organics, and Gases in Deuterated Solvents Relevant to the Organometallic Chemist," can be found under https://authors.library.caltech.edu/records/q3m5b-fec90, **n.d.**
[2] L. N. Lameijer, D. Ernst, S. L. Hopkins, M. S. Meijer, S. H. C. Askes, S. E. Le Dévédec, S. Bonnet, *Angewandte Chemie - International Edition* **2017**, *56*, 11549–11553.
[3] A. L. Spek, *Acta Crystallogr D Biol Crystallogr* **2009**, *65*, 148–155.
[4] G. M. Sheldrick, *Acta Cryst C* **2015**, *71*, 3–8.
